# Supplementary material for: Leveraging Foundation Models for the Characterization of Small-RNA Properties
Source: Comput Struct Biotechnol J. 2026 Sep 22;35(1):0224. doi: 10.34133/csbj.0224 (PMC13594420; doi:10.34133/csbj.0224)
Supplement: Supplementary file 1 [file csbj.0224.f1.pdf]

Supplementary Figures

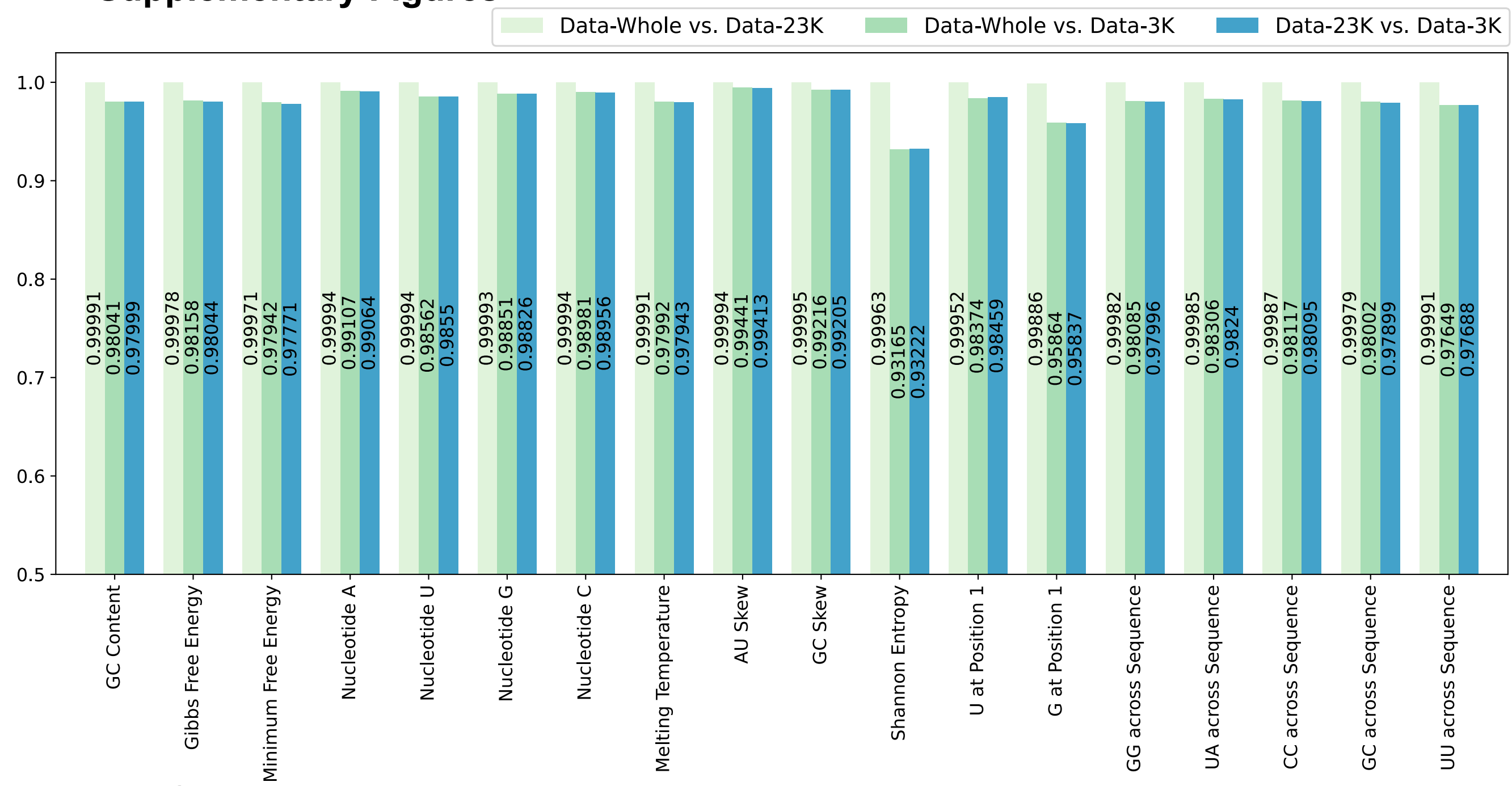

Fig. S1. Agreement among Data-Whole, Data-23K, and Data-3K in the correlations between interpretable features and RNA-FM embedding dimensions, quantified using Pearson correlation. Data-3K is restricted to human miRNA sequences.

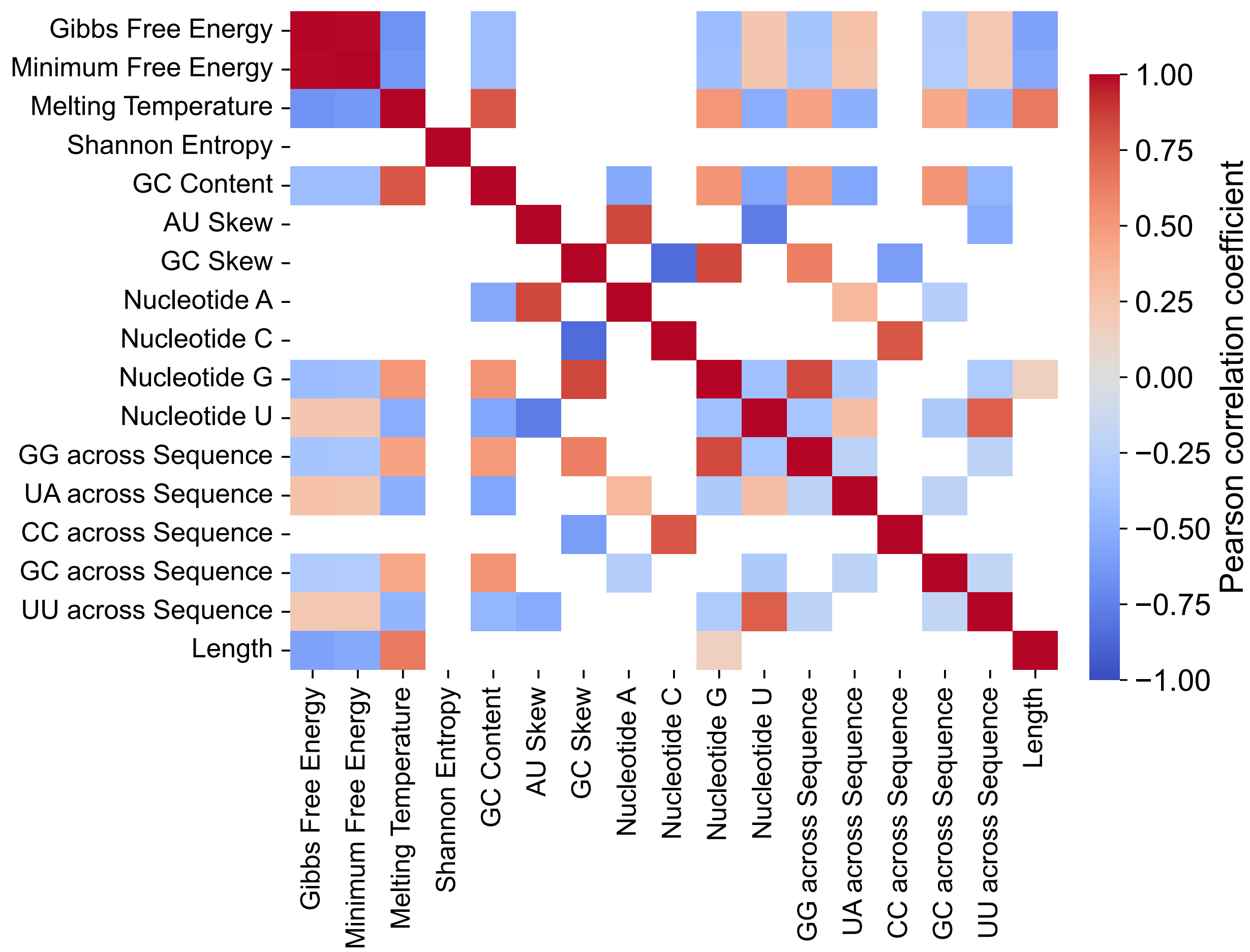

Fig. S2. Correlation heatmap illustrating pairwise Pearson correlation coefficients among extracted interpretable features and sequence length across Data-3K, with non-significant correlations ($p \geq 0.05$) left blank.

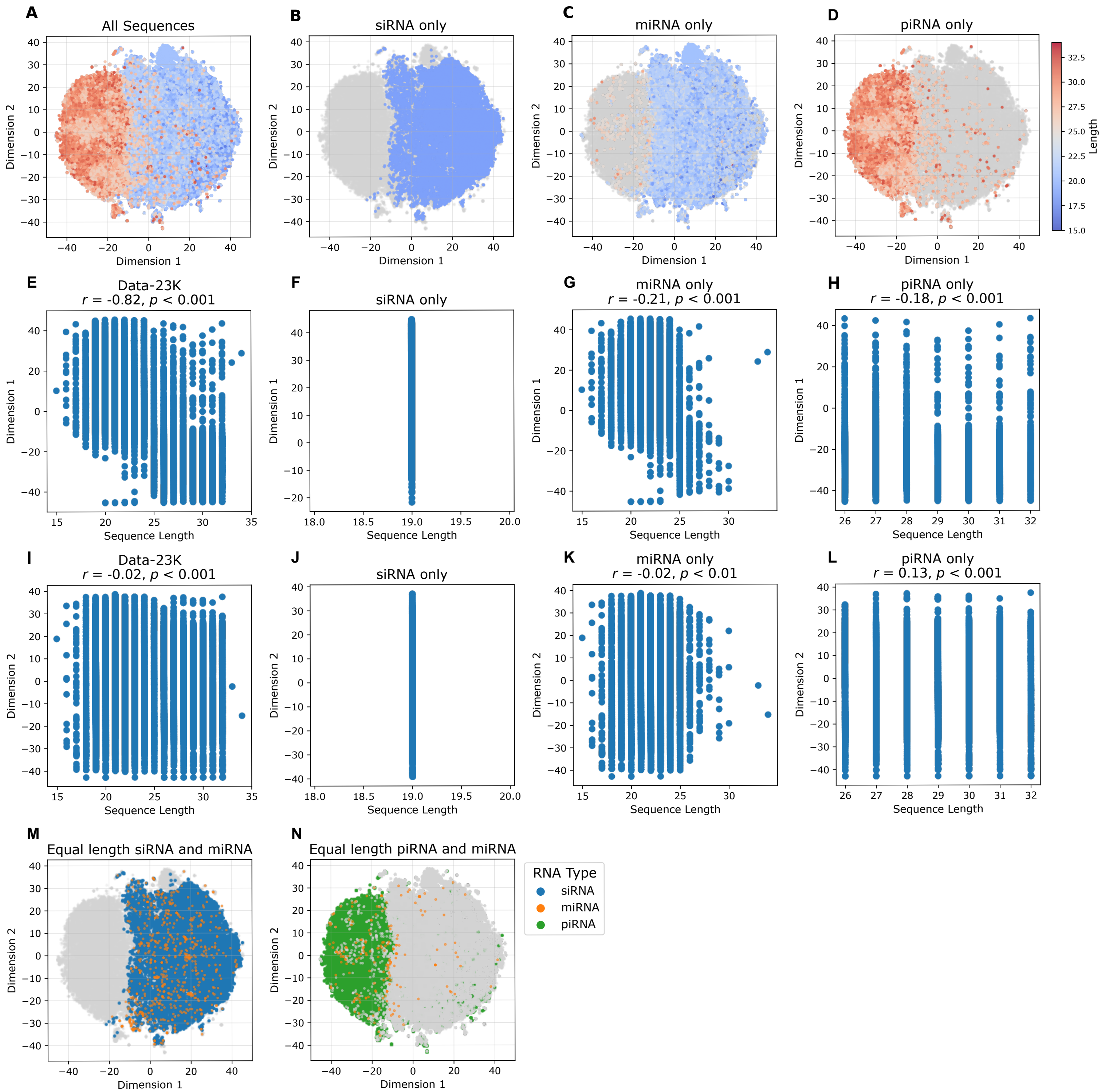

Fig. S3. Analysis of sequence length effect on RNA-FM embeddings. (A to D) t-SNE visualisation of RNA-FM embeddings coloured according to the lengths of (A) all sequences, (B) siRNAs only, (C) miRNAs only, and (D) piRNAs only. (E to L) Scatter plot illustrating t-SNE dimensions versus sequence length for (E,I) all sequences, (F,J) siRNAs only, (G,K) miRNAs only, and (H,L) piRNAs only, with corresponding Pearson correlation coefficient, *r*, and *p*-value shown in the subtitle. (M to N) t-SNE visualisation of RNA-FM embeddings with equal length (M) siRNA (blue) and miRNA (orange), and (N) piRNA (green) and miRNA (orange) annotated.

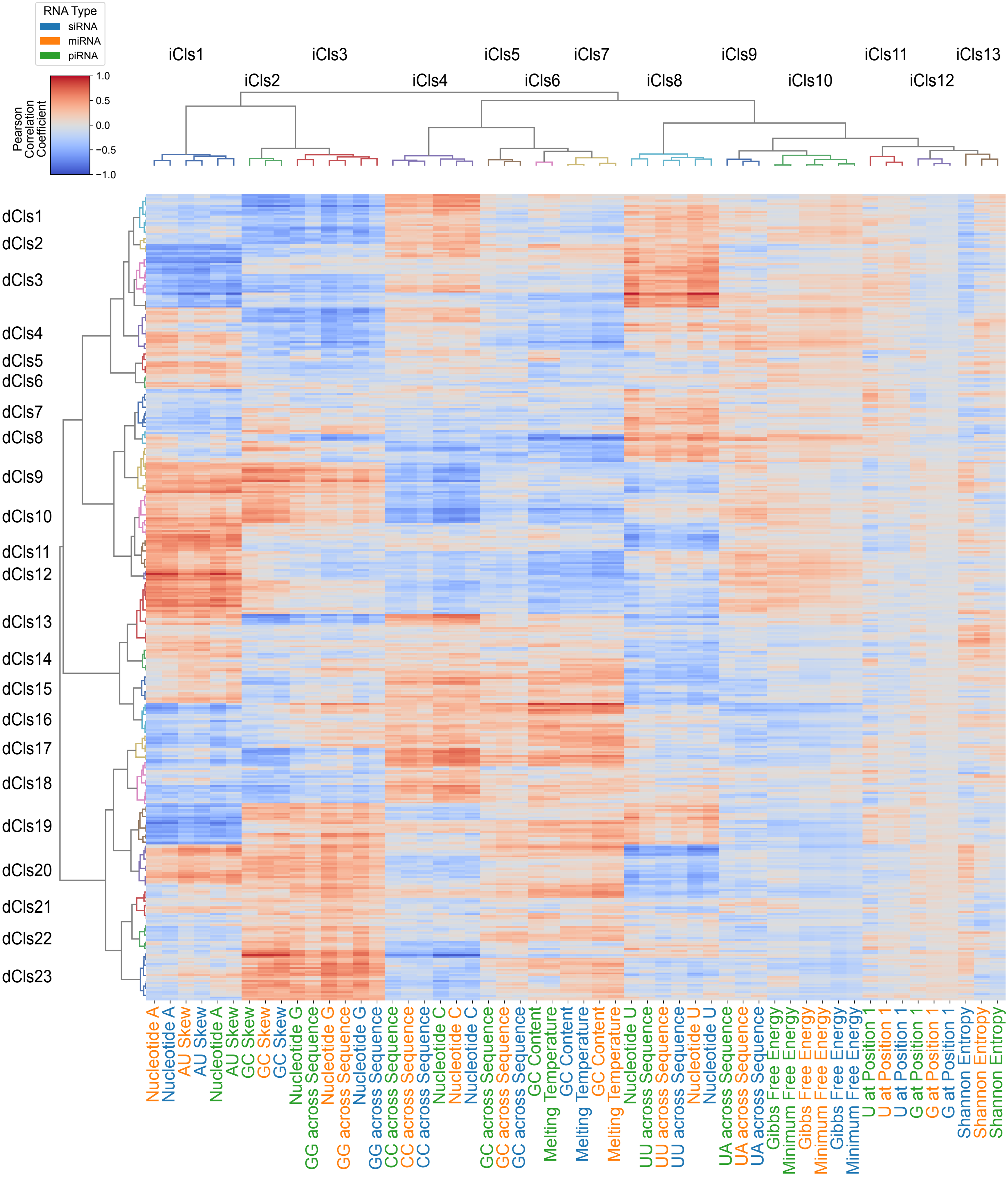

Fig. S4. Heatmap of Pearson correlations between interpretable features and RNA-FM features with hierarchical clustering for each RNA type from Data-23K. Each cluster is coloured based on empirically defined thresholds. Each interpretable feature is coloured according to RNA type: siRNA (blue), miRNA (orange), and piRNA (green). iCls, interpretable features clusters; dCls, deep learning feature clusters.

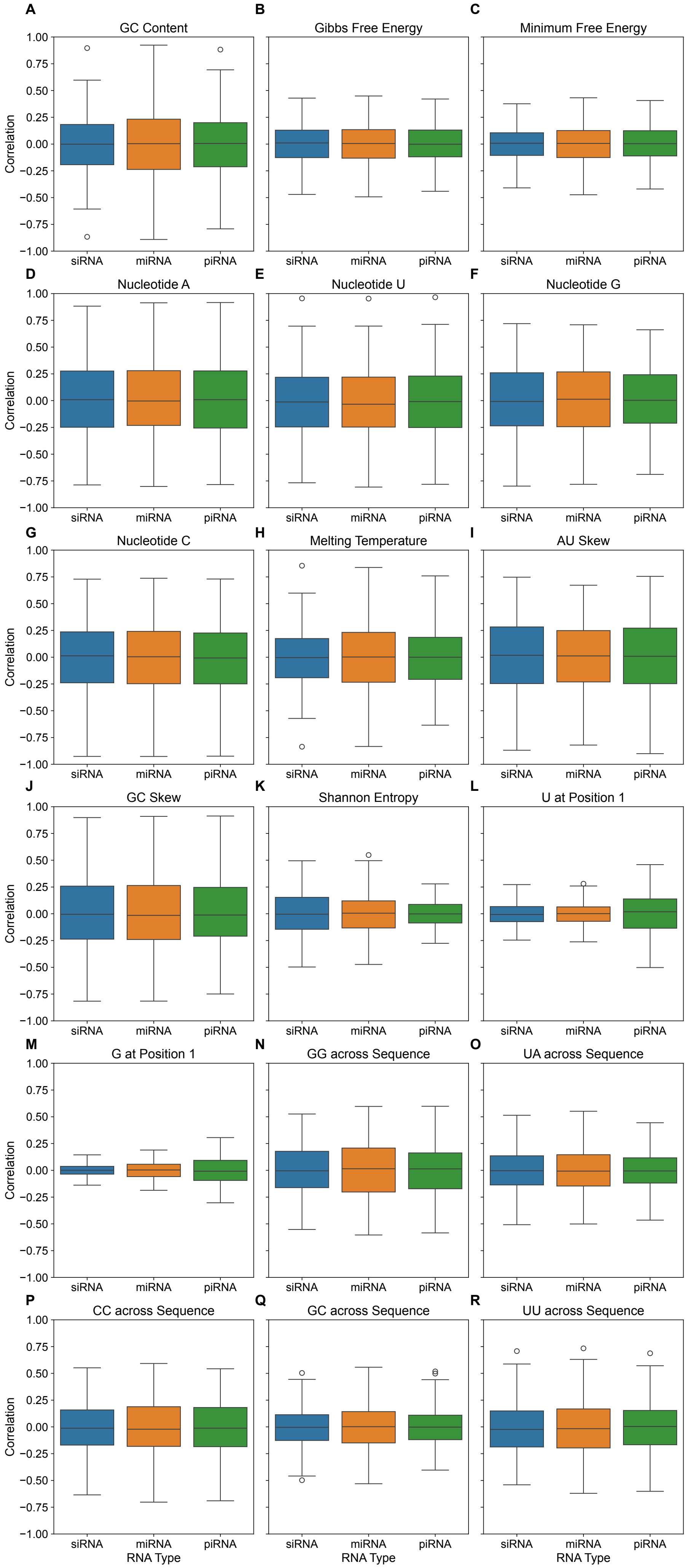

Fig. S5. Box plots for each interpretable feature showcasing the Pearson correlation for each RNA type based on Data-23K. Features include, (A) GC Content, (B) Gibbs free energy, (C) minimum free energy, (D to G) nucleotides A, U, G, C, (H) melting temperature, (I) AU skew, (J) GC skew, (K) Shannon entropy, (L) nucleotide U at position 1, (M) nucleotide G at position 1, (N to R) dinucleotides GG, UA, CC, GC, UU across sequence.

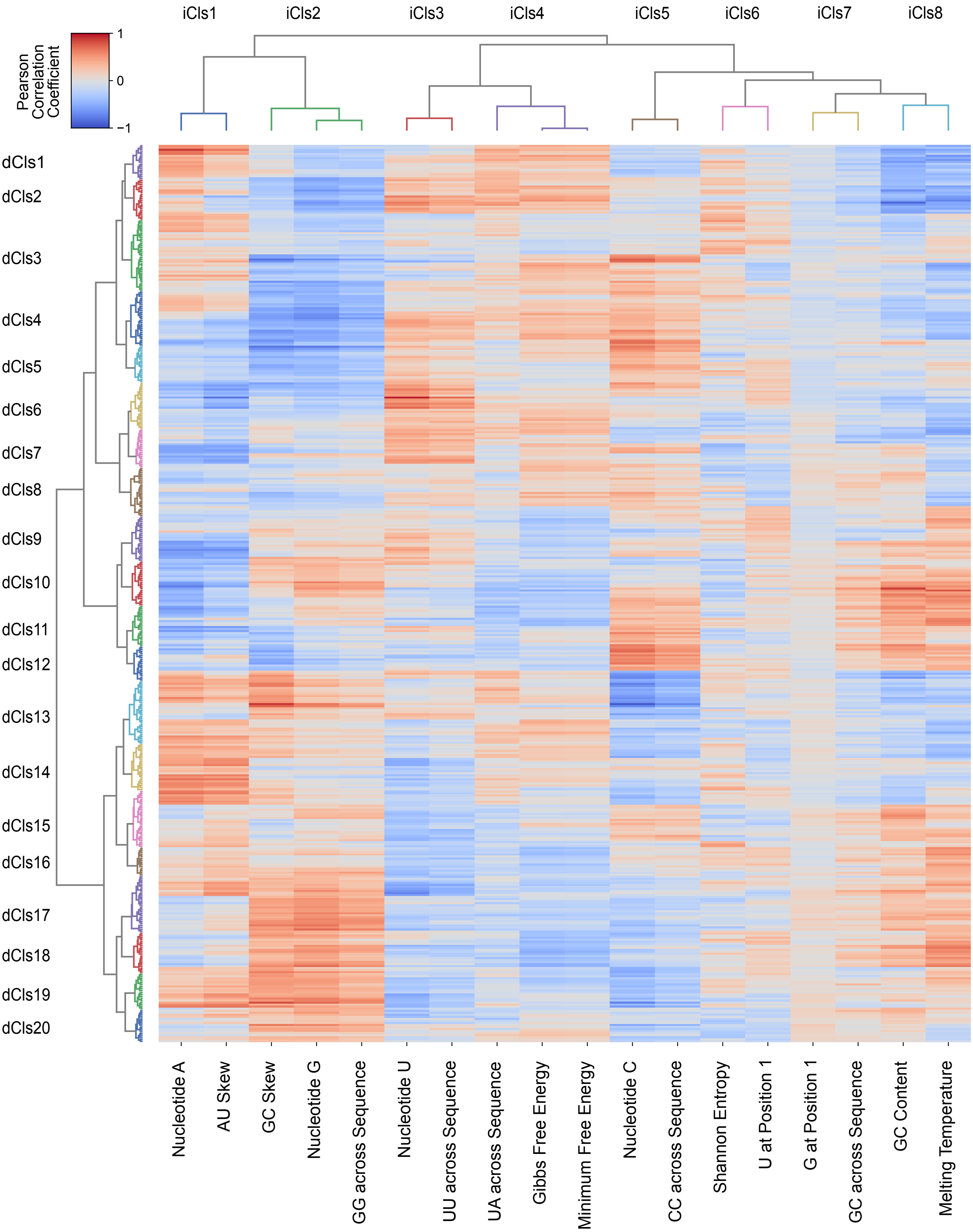

Fig. S6. Heatmap of Pearson correlations between interpretable features and RNA-FM features with hierarchical clustering for Data-3K using the All Types data. Each cluster is coloured based on empirically defined thresholds. iCls, interpretable feature clusters; dCls, deep learning feature clusters.

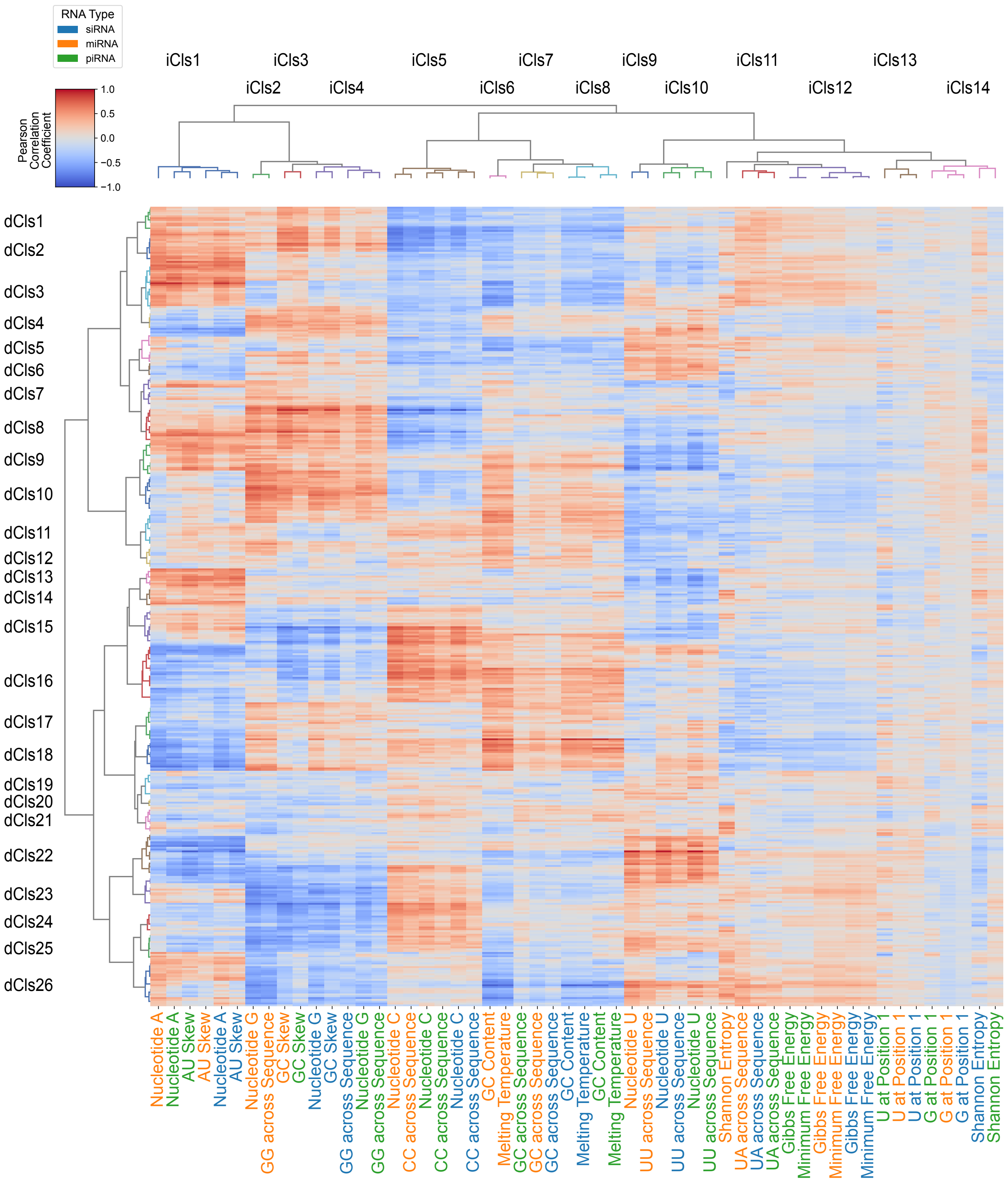

Fig. S7. Heatmap of Pearson correlations between interpretable features and RNA-FM features with hierarchical clustering for each RNA type for Data-3K. Each cluster is coloured based on empirically defined thresholds. Each interpretable feature is coloured according to RNA type: siRNA (blue), miRNA (orange), and piRNA (green). iCls, interpretable feature clusters; dCls, deep learning feature clusters.

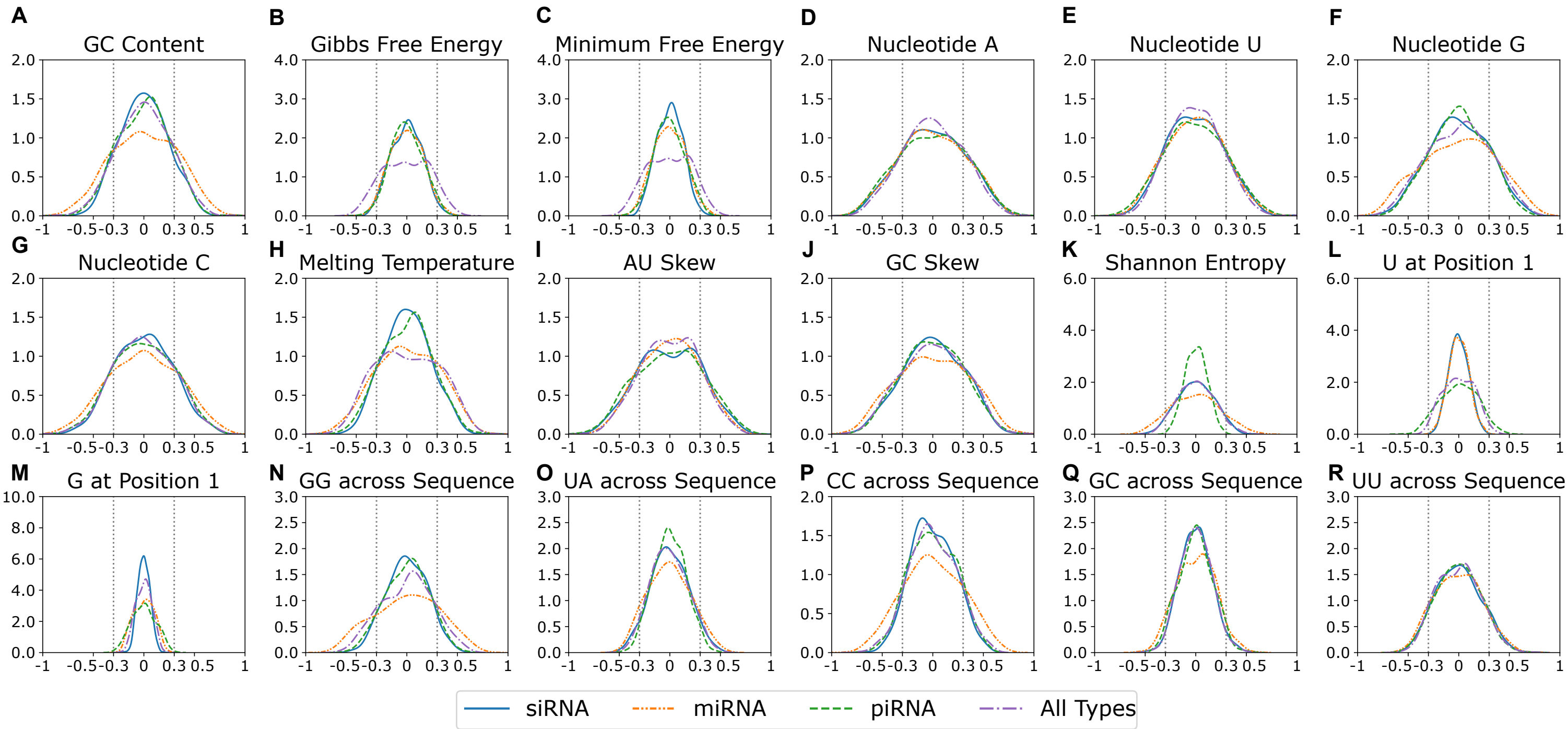

Fig. S8. Kernel density estimation of Pearson correlation values (x-axis) for each interpretable feature for siRNAs (blue), miRNAs (orange), piRNAs (green), and All Types (purple) in Data-3K. Dotted vertical lines indicate correlation values of -0.3 and 0.3.

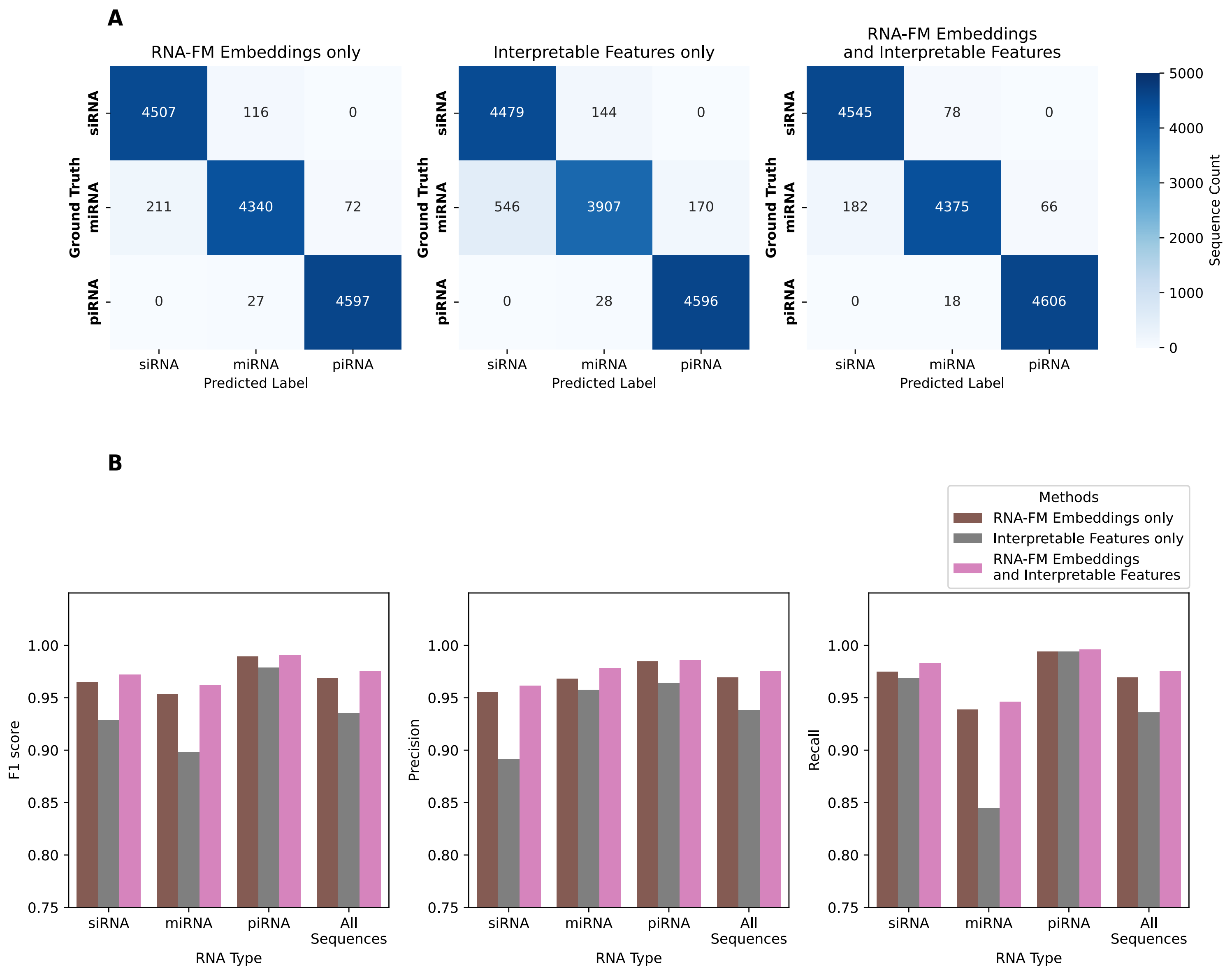

Fig. S9. Small-RNA classification performance across feature sets. (A) Confusion matrix of the ablation study across three feature sets: RNA-FM embeddings only, interpretable features only, and RNA-FM embeddings and interpretable features combined. (B) RNA classification performance evaluated via F1 score, precision and recall from the ablation study for RNA-FM embeddings only (brown), interpretable features only (grey), and RNA-FM embeddings and interpretable features combined (pink).

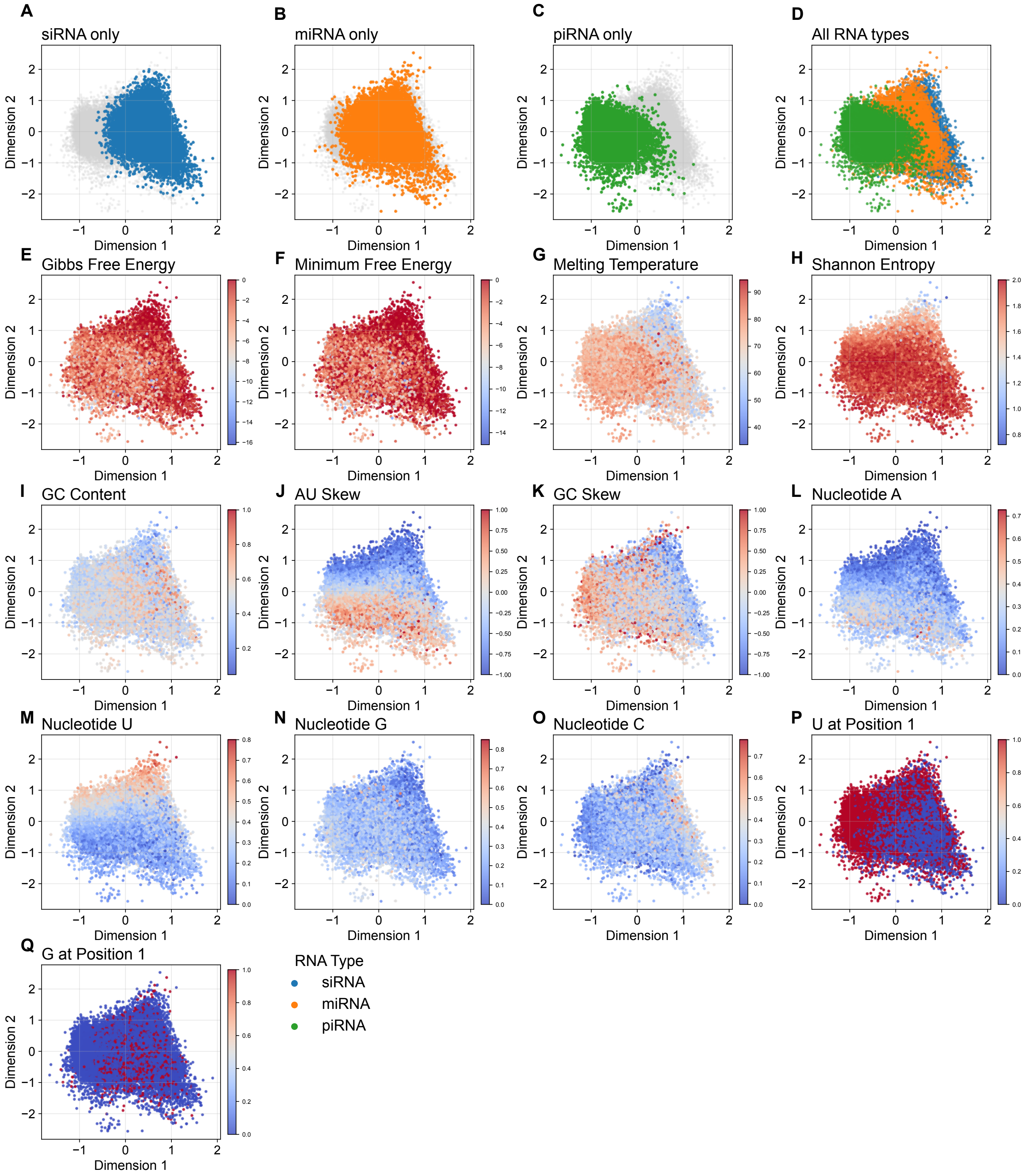

Fig. S10. PCA visualisation of RNA-FM embeddings across all RNA types and feature annotations using the first two components. All panels show the same two-dimensional PCA projection of RNA embeddings. Sequences are highlighted by RNA class in (A) siRNA (blue), (B) miRNA (orange) and (C) piRNA (green), and all three classes are shown together in (D). In (E to Q), sequences are coloured according to the values of each interpretable feature with blue hues indicating lower feature values and red hues indicating higher feature values.

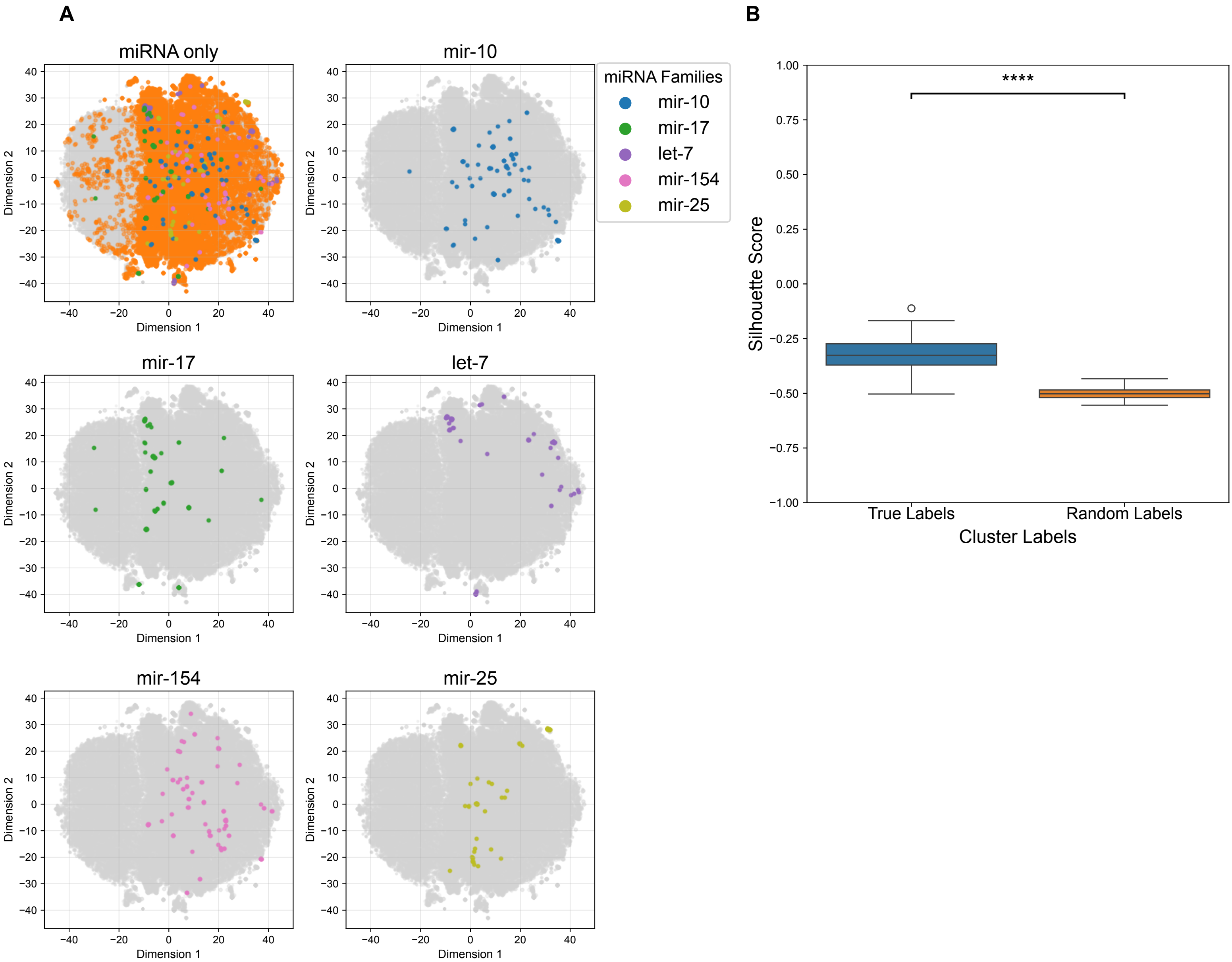

Fig. S11. (A) t-SNE visualisation of RNA-FM embeddings with miRNAs from five most abundant miRNA families annotated accordingly: mir-10 (blue), mir-17 (green), let-7 (purple), mir-154 (pink), and mir-25 (yellow), with remaining miRNAs shown in orange. (B) Boxplot showing the clustering performance of miRNA families with more than 20 sequences is significantly better than randomly assigned embeddings. ****, $p < 0.0001$.

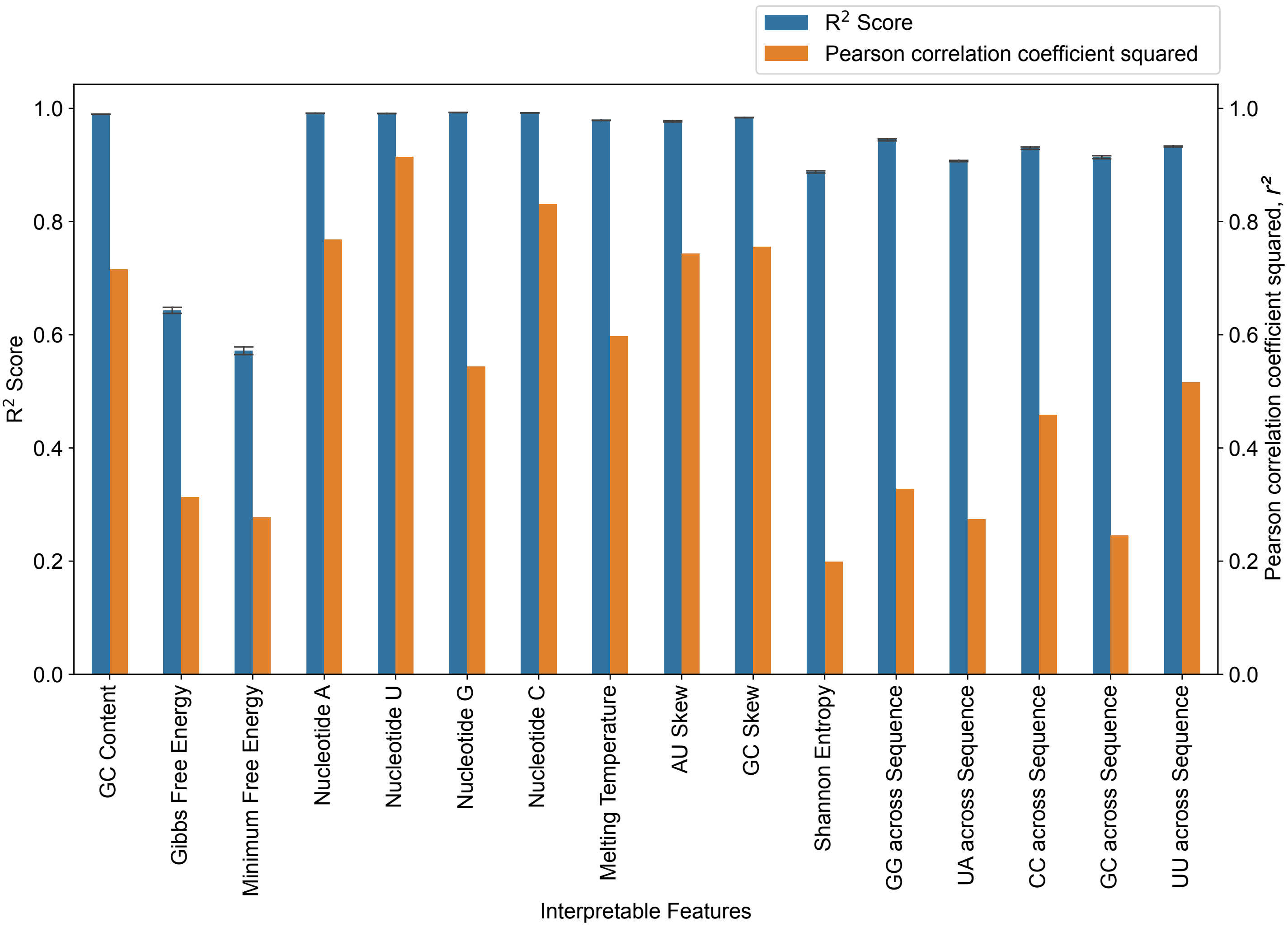

Fig. S12. Bar chart showing R^2 score for a linear regression model (left y-axis) across all interpretable features based on 5-fold cross validation. The right y-axis shows the highest squared absolute Pearson correlation coefficient (r^2) between interpretable feature values and RNA-FM embedding dimensions across all interpretable features. Error bars show standard deviation across 5-fold cross validation.

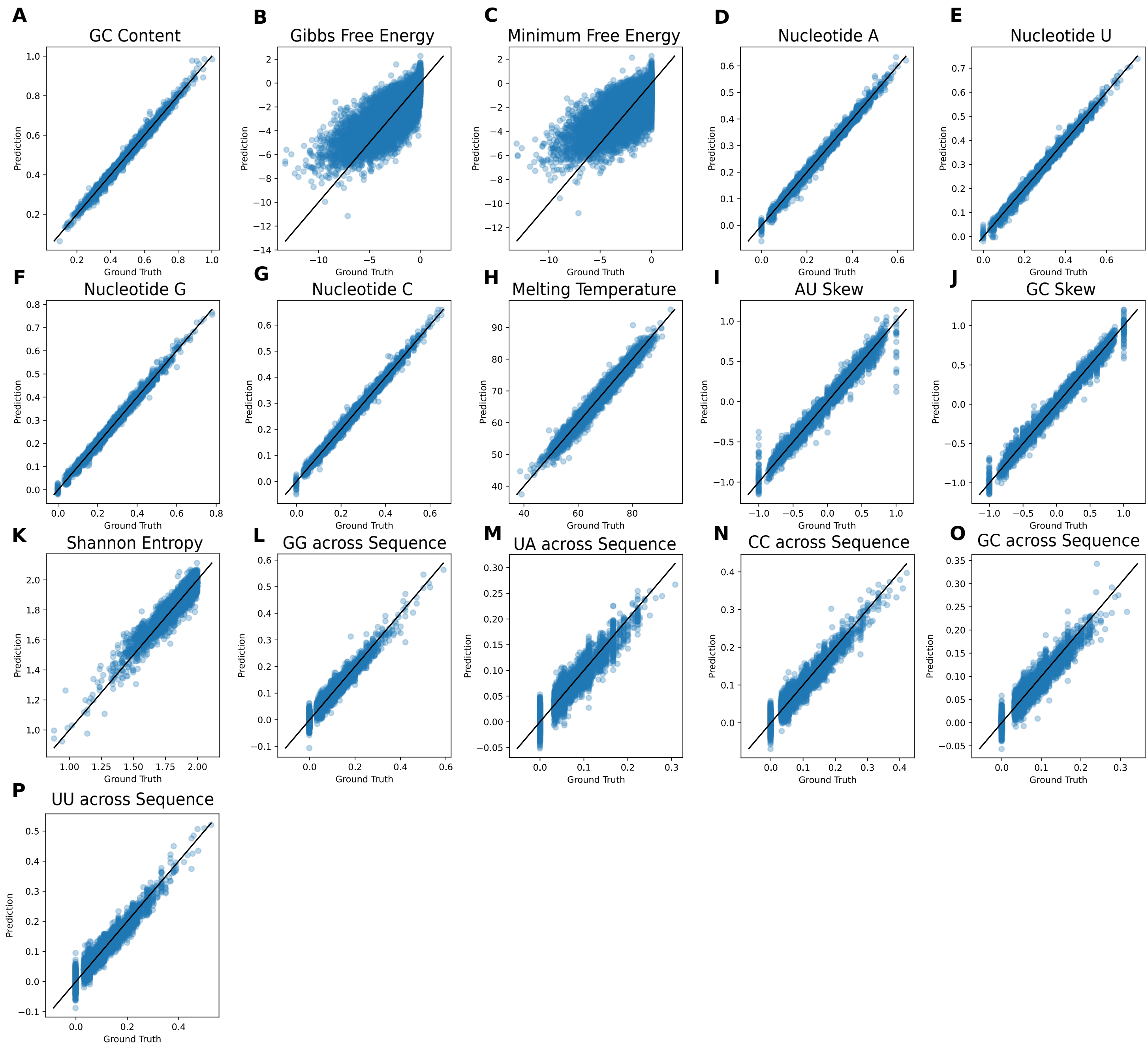

Fig. S13. Scatter plot of ground truth versus prediction using linear regression across (A to P) all interpretable features, except for nucleotides U and G at position 1, on the held-out test set, with black lines representing perfect prediction (y=x).

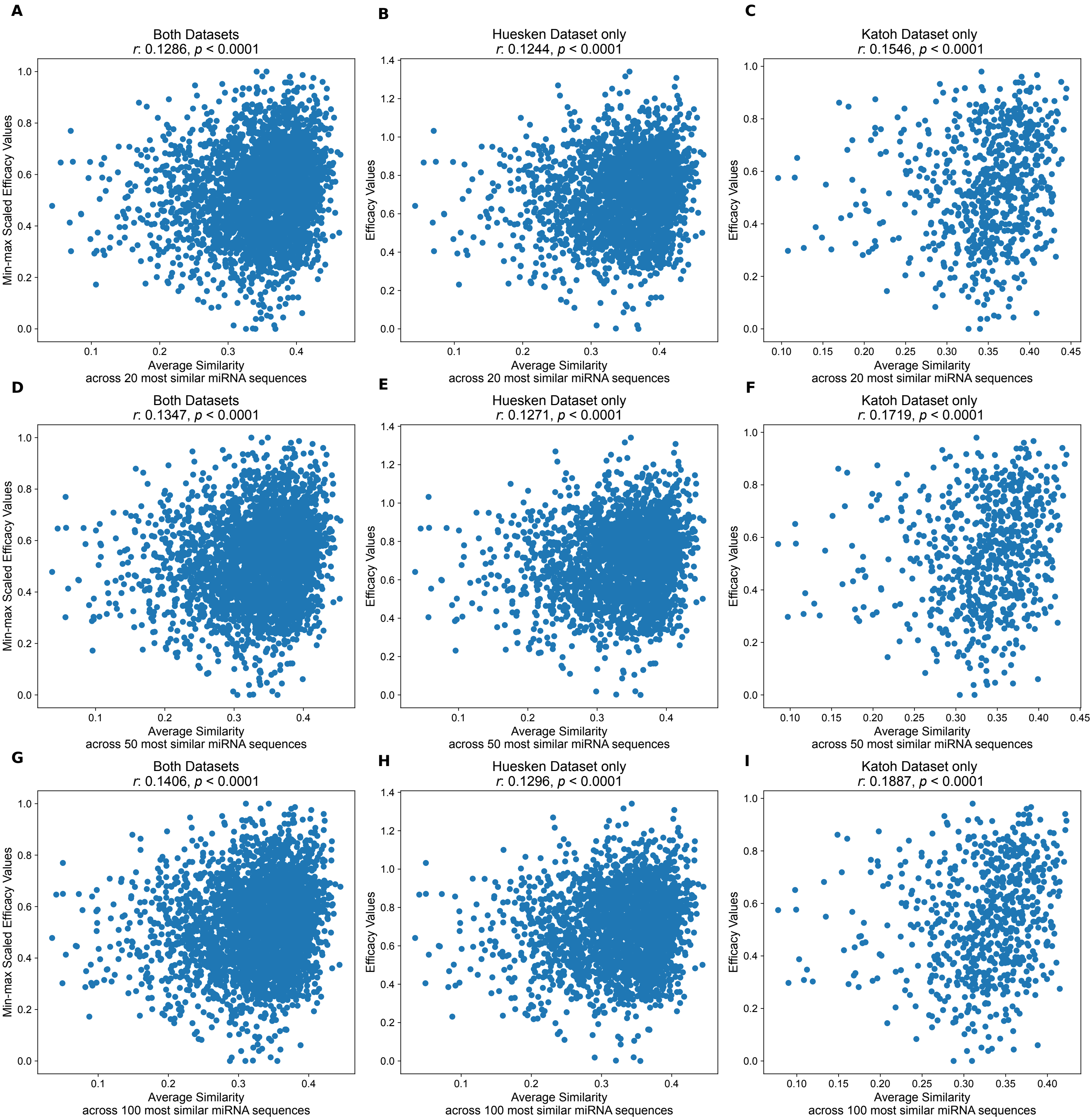

Fig. S14. Scatter plot of average similarity across (A to C) 20, (D to F) 50, (G to I) 100 most similar miRNA sequences versus the targeting efficacy of siRNAs from (A, D, G) both Huesken and Katoh datasets, (B, E, H) Huesken dataset only, and (C, F, I) Katoh dataset only, with Pearson correlation coefficient, *r*, and corresponding p-value, *p*, labelled. Min-max scaled efficacy values are used when both datasets are combined; whereas individual datasets use their original efficacy values.

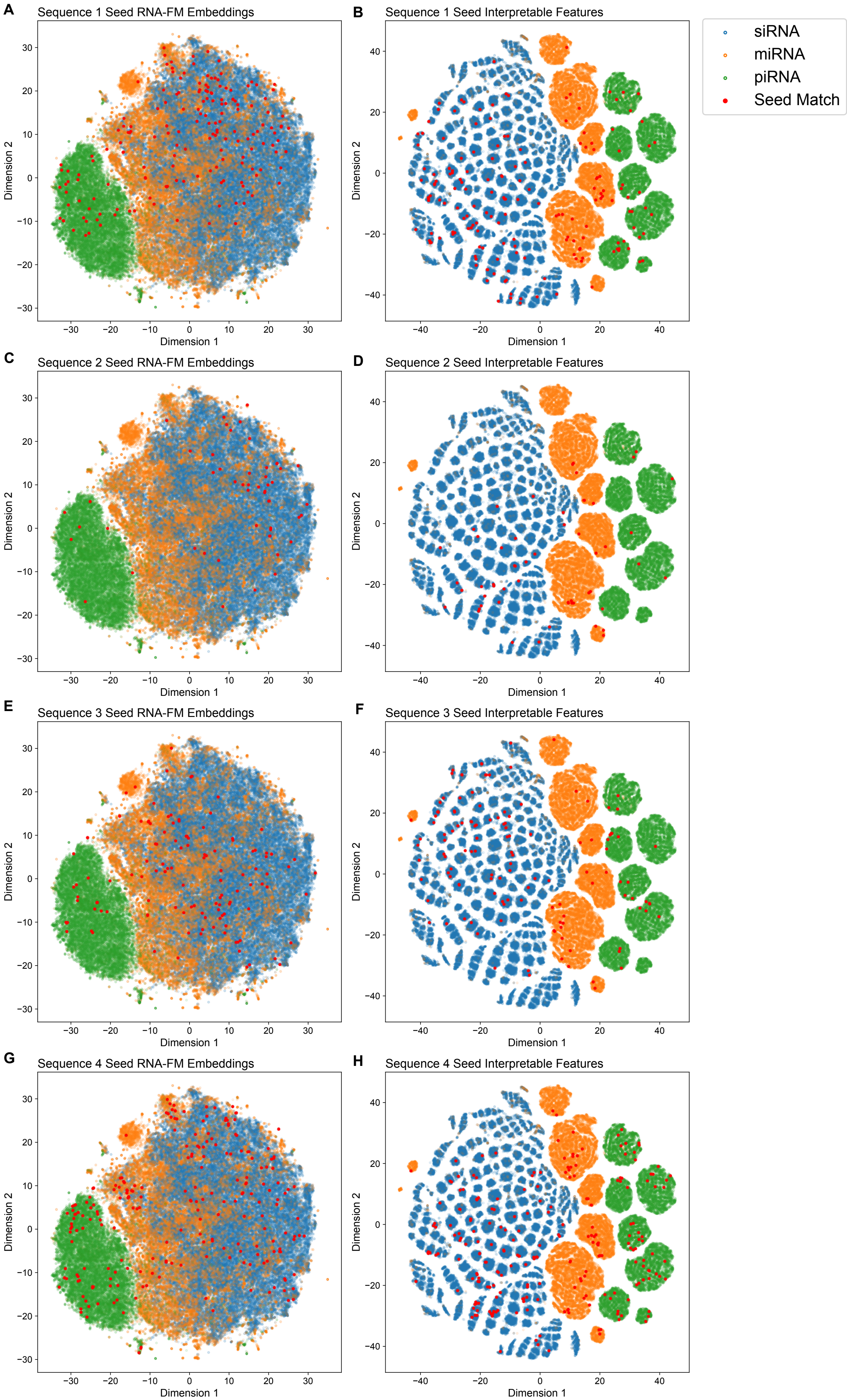

Fig. S15. Example of using RNAExplorer to investigate sequences containing certain seed fragments. t-SNE projections of RNA-FM embeddings for sequences containing seed fragments from Sequences (A, C, E, G) 1–4 along with the (B, D, F, H) corresponding t-SNE projections of interpretable features. All sequences are coloured by RNA type: siRNA (blue), miRNA (orange), and piRNA (green), with the seed-matched sequences highlighted in red.

Supplementary Tables

Table S1. RNA type classification performance of the Support Vector Machine on the held-out test set from the ablation study, reported as F1 score, precision, recall and accuracy.

Data	F1 score	Precision	Recall	Accuracy
Interpretable Features only	0.935	0.938	0.936	0.936
RNA-FM embeddings only	0.969	0.969	0.969	0.969
RNA-FM embeddings and Interpretable Features combined	0.975	0.975	0.975	0.975

Table S2. Clustering performance of t-SNE reduced RNA-FM embeddings with ground truth RNA type labels versus randomly assigned labels, evaluated by silhouette score. Silhouette scores are reported for both macro-average across all RNA types and for siRNA, miRNA and piRNA, respectively. For silhouette scores, large positive values indicate better separation between labels, whereas values near zero indicate overlap between clusters, and negative values indicate likely misassignment.

	RNA Type	Silhouette Score
True labels	Macro-Average	0.181
	siRNA	0.145
	miRNA	-0.052
	piRNA	0.451
Random labels	Macro-Average	-0.002
	siRNA	-0.002
	miRNA	0.000
	piRNA	-0.004

Table S3. Clustering performance of PCA-reduced RNA-FM embeddings using the first two components with ground truth RNA type labels versus randomly assigned labels, evaluated by silhouette score. Silhouette scores are reported for both macro-average across all RNA types and for siRNA, miRNA and piRNA, respectively. For silhouette scores, large positive values indicate better separation between labels, whereas values near zero indicate overlap between clusters, and negative values indicate likely misassignment.

	RNA Type	Silhouette Score
True labels	Macro-average	0.174
	siRNA	0.100
	miRNA	-0.030
	piRNA	0.451
Random labels	Macro-average	-0.002
	siRNA	-0.001
	miRNA	0.001
	piRNA	-0.005

Table S4. Prediction performance of linear regression models based on 5-fold cross validation (mean ± standard deviation) and held-out test set across all interpretable features with continuous values, reported as R^2 Score.

Interpretable Features	R^2 Score based on 5-fold Cross Validation	R^2 Score based on held-out test set
Nucleotide A	0.991 ± 0.0002	0.992
Nucleotide U	0.991 ± 0.0001	0.991
Nucleotide G	0.993 ± 0.0001	0.993
Nucleotide C	0.992 ± 0.0002	0.992
GC Content	0.989 ± 0.0001	0.989
GC Skew	0.984 ± 0.0002	0.984
Melting Temperature	0.979 ± 0.0003	0.978
AU Skew	0.977 ± 0.0011	0.977
GG across Sequence	0.944 ± 0.0019	0.944
UU across Sequence	0.933 ± 0.0011	0.934
CC across Sequence	0.930 ± 0.0021	0.93
GC across Sequence	0.914 ± 0.0026	0.914
UA across Sequence	0.907 ± 0.0010	0.906
Shannon Entropy	0.888 ± 0.0020	0.888
Gibbs Free Energy	0.643 ± 0.0053	0.649
Minimum Free Energy	0.572 ± 0.0068	0.58

Table S5. Prediction performance of logistic regression models based on 5-fold cross validation (mean ± standard deviation) and held-out test set across all interpretable features with binary values, reported as AUC Score, respectively.

Interpretable Features	AUC Score based on 5-fold Cross Validation	AUC Score based on held-out test set
U at Position 1	0.997 ± 0.0004	0.997
G at Position 1	0.991 ± 0.0011	0.989

Table S6. Top 20 Most Similar RNA Sequences for Each Query Sequence.

	Sequence ID	siRNA Sequence	Rank	Type	Sequence	Similarity	Length	GC %	GC skew	AT/AU skew	MNC A	MNC C	MNC G	MNC U	Species	Link
**0**	Sequence 1	UGUCACUUGCUCUUUGGUC	1	siRNA	UCACUCUUGUGCUUUGUUC	0.469	19	42.11	-0.25	-0.818	0.053	0.263	0.158	0.526	Homo Sapiens	
**1**	Sequence 1	UGUCACUUGCUCUUUGGUC	2	siRNA	UACUUGUUUGUCUCUGGCC	0.463	19	47.37	-0.111	-0.8	0.053	0.263	0.211	0.474	Homo Sapiens	
**2**	Sequence 1	UGUCACUUGCUCUUUGGUC	3	siRNA	GUGCUCGUCACCUUUGUCU	0.458	19	52.63	-0.2	-0.778	0.053	0.316	0.211	0.421	Homo Sapiens	
**3**	Sequence 1	UGUCACUUGCUCUUUGGUC	4	miRNA	UGCUCUCUACCGUUGUCAUG	0.454	20	50	-0.2	-0.6	0.1	0.3	0.2	0.4	Eugenia uniflora	https://mirbase.org/mature/MIMAT0041093
**4**	Sequence 1	UGUCACUUGCUCUUUGGUC	5	siRNA	UUCACGUGGUUCUGUGCUC	0.451	19	52.63	0	-0.778	0.053	0.263	0.263	0.421	Homo Sapiens	
**5**	Sequence 1	UGUCACUUGCUCUUUGGUC	6	siRNA	UUUUGUAUGUUCUCUGGCC	0.449	19	42.11	0	-0.818	0.053	0.211	0.211	0.526	Homo Sapiens	
**6**	Sequence 1	UGUCACUUGCUCUUUGGUC	7	siRNA	UGUCAUUUGCUUCUUGUCC	0.449	19	42.11	-0.25	-0.818	0.053	0.263	0.158	0.526	Homo Sapiens	
**7**	Sequence 1	UGUCACUUGCUCUUUGGUC	8	miRNA	UUCGUGUGCCAUCGUCCUUUGU	0.448	22	50	-0.091	-0.818	0.045	0.273	0.227	0.455	Gallus gallus	https://mirbase.org/mature/MIMAT0049915
**8**	Sequence 1	UGUCACUUGCUCUUUGGUC	9	siRNA	GUUCUCGCCAUUUGUGCUC	0.447	19	52.63	-0.2	-0.778	0.053	0.316	0.211	0.421	Homo Sapiens	
**9**	Sequence 1	UGUCACUUGCUCUUUGGUC	10	siRNA	UUCUCCGUGACUCUUGUAU	0.446	19	42.11	-0.25	-0.636	0.105	0.263	0.158	0.474	Homo Sapiens	
**10**	Sequence 1	UGUCACUUGCUCUUUGGUC	11	siRNA	UUGUUGUCAGUGUCCUUCC	0.445	19	47.37	-0.111	-0.8	0.053	0.263	0.211	0.474	Homo Sapiens	
**11**	Sequence 1	UGUCACUUGCUCUUUGGUC	12	siRNA	UGUCUCAUGUCUAGUUGCC	0.444	19	47.37	-0.111	-0.6	0.105	0.263	0.211	0.421	Homo Sapiens	
**12**	Sequence 1	UGUCACUUGCUCUUUGGUC	13	siRNA	UCUCUCAAUGUCUUGUUUG	0.442	19	36.84	-0.143	-0.667	0.105	0.211	0.158	0.526	Homo Sapiens	
**13**	Sequence 1	UGUCACUUGCUCUUUGGUC	14	siRNA	UGUCUCGCUGUUCCAGUUG	0.44	19	52.63	0	-0.778	0.053	0.263	0.263	0.421	Homo Sapiens	
**14**	Sequence 1	UGUCACUUGCUCUUUGGUC	15	siRNA	GUUGCCUCUUACUCUUGAC	0.44	19	47.37	-0.333	-0.6	0.105	0.316	0.158	0.421	Homo Sapiens	
**15**	Sequence 1	UGUCACUUGCUCUUUGGUC	16	siRNA	UCGCUUUGUCCUAUUGUGG	0.439	19	47.37	0.111	-0.8	0.053	0.211	0.263	0.474	Homo Sapiens	
**16**	Sequence 1	UGUCACUUGCUCUUUGGUC	17	miRNA	AGAGUGUUGCUCUCAUCUCU	0.439	20	45	-0.111	-0.455	0.15	0.25	0.2	0.4	Pongo pygmaeus	https://mirbase.org/mature/MIMAT0016050
**17**	Sequence 1	UGUCACUUGCUCUUUGGUC	18	siRNA	GUCUUCAUUGGUGCCUUUC	0.438	19	47.37	-0.111	-0.8	0.053	0.263	0.211	0.474	Homo Sapiens	
**18**	Sequence 1	UGUCACUUGCUCUUUGGUC	19	siRNA	AUGCGUUCUAUGUCUCUGG	0.437	19	47.37	0.111	-0.6	0.105	0.211	0.263	0.421	Homo Sapiens	
**19**	Sequence 1	UGUCACUUGCUCUUUGGUC	20	siRNA	UGUCUCGUUGGCUAUUUGC	0.437	19	47.37	0.111	-0.8	0.053	0.211	0.263	0.474	Homo Sapiens	
**20**	Sequence 2	ACACCCGUCACCACUGCUC	1	siRNA	AUAACCGCCUCCACUCCUG	0.327	19	57.89	-0.636	0	0.211	0.474	0.105	0.211	Homo Sapiens	
**21**	Sequence 2	ACACCCGUCACCACUGCUC	2	siRNA	UAACUCCGCCCACACGUCC	0.309	19	63.16	-0.667	0.143	0.211	0.526	0.105	0.158	Homo Sapiens	
**22**	Sequence 2	ACACCCGUCACCACUGCUC	3	siRNA	CCAGUCAACACAUCGCUCC	0.308	19	57.89	-0.636	0.25	0.263	0.474	0.105	0.158	Homo Sapiens	
**23**	Sequence 2	ACACCCGUCACCACUGCUC	4	siRNA	UCACCCAACACUGCGUCCC	0.306	19	63.16	-0.667	0.143	0.211	0.526	0.105	0.158	Homo Sapiens	
**24**	Sequence 2	ACACCCGUCACCACUGCUC	5	siRNA	AACCGCACCUCACCAUCUC	0.306	19	57.89	-0.818	0.25	0.263	0.526	0.053	0.158	Homo Sapiens	
**25**	Sequence 2	ACACCCGUCACCACUGCUC	6	siRNA	ACACGAUCUCCUCAUGCCC	0.305	19	57.89	-0.636	0	0.211	0.474	0.105	0.211	Homo Sapiens	
**26**	Sequence 2	ACACCCGUCACCACUGCUC	7	siRNA	CUCCGCACCACCUAUGACU	0.303	19	57.89	-0.636	0	0.211	0.474	0.105	0.211	Homo Sapiens	
**27**	Sequence 2	ACACCCGUCACCACUGCUC	8	siRNA	AGUCGCACCACAUCUCCAC	0.302	19	57.89	-0.636	0.25	0.263	0.474	0.105	0.158	Homo Sapiens	
**28**	Sequence 2	ACACCCGUCACCACUGCUC	9	siRNA	GAACCCUCCACAUCUGCUC	0.302	19	57.89	-0.636	0	0.211	0.474	0.105	0.211	Homo Sapiens	
**29**	Sequence 2	ACACCCGUCACCACUGCUC	10	siRNA	AGUCACUGCACCUCACACC	0.301	19	57.89	-0.636	0.25	0.263	0.474	0.105	0.158	Homo Sapiens	
**30**	Sequence 2	ACACCCGUCACCACUGCUC	11	siRNA	UACAUCUGGCCCCAUCACC	0.301	19	57.89	-0.636	0	0.211	0.474	0.105	0.211	Homo Sapiens	
**31**	Sequence 2	ACACCCGUCACCACUGCUC	12	siRNA	UCGCGCACCAACACUCCCA	0.295	19	63.16	-0.667	0.429	0.263	0.526	0.105	0.105	Homo Sapiens	
**32**	Sequence 2	ACACCCGUCACCACUGCUC	13	siRNA	UAACCCUCCACACCACUCG	0.295	19	57.89	-0.818	0.25	0.263	0.526	0.053	0.158	Homo Sapiens	
**33**	Sequence 2	ACACCCGUCACCACUGCUC	14	siRNA	UACCCGACCACUGUCCUCC	0.295	19	63.16	-0.667	-0.143	0.158	0.526	0.105	0.211	Homo Sapiens	
**34**	Sequence 2	ACACCCGUCACCACUGCUC	15	siRNA	AACCGCUCCACCUCUGUCA	0.294	19	57.89	-0.636	0	0.211	0.474	0.105	0.211	Homo Sapiens	
**35**	Sequence 2	ACACCCGUCACCACUGCUC	16	siRNA	CACCCCUGGCCCUCCACUA	0.294	19	68.42	-0.692	0	0.158	0.579	0.105	0.158	Homo Sapiens	
**36**	Sequence 2	ACACCCGUCACCACUGCUC	17	miRNA	GAUUGCACUACCCAUCGCCCAC	0.294	22	59.1	-0.538	0.111	0.227	0.455	0.136	0.182	Echinococcus granulosus;Echinococcus multilocularis	https://mirbase.org/mature/MIMAT0041731;https://mirbase.org/mature/MIMAT0041759
**37**	Sequence 2	ACACCCGUCACCACUGCUC	18	siRNA	AUUCCCAUCAGUCACACCU	0.293	19	47.37	-0.778	0	0.263	0.421	0.053	0.263	Homo Sapiens	
**38**	Sequence 2	ACACCCGUCACCACUGCUC	19	siRNA	CCGCUCACAGCCUCUAAUC	0.292	19	57.89	-0.636	0	0.211	0.474	0.105	0.211	Homo Sapiens	

**39**	Sequence 2	ACACCCGUCACCACUGCUC	20	siRNA	AGCCACAACUCCUCCGACC	0.291	19	63.16	-0.667	0.429	0.263	0.526	0.105	0.105	Homo Sapiens	
**40**	Sequence 3	UUAGGCUUCAGGUUCGUAG	1	siRNA	UUAGGCUUCAGGUCCGUGA	0.502	19	52.63	0.2	-0.333	0.158	0.211	0.316	0.316	Homo Sapiens	
**41**	Sequence 3	UUAGGCUUCAGGUUCGUAG	2	siRNA	UCAAGGUUUAGGGUGCUUC	0.411	19	47.37	0.333	-0.4	0.158	0.158	0.316	0.368	Homo Sapiens	
**42**	Sequence 3	UUAGGCUUCAGGUUCGUAG	3	siRNA	UAGGGUUUAGGACUGCUCC	0.41	19	52.63	0.2	-0.333	0.158	0.211	0.316	0.316	Homo Sapiens	
**43**	Sequence 3	UUAGGCUUCAGGUUCGUAG	4	siRNA	UUGCGUUCUAGGUACAGGU	0.391	19	47.37	0.333	-0.4	0.158	0.158	0.316	0.368	Homo Sapiens	
**44**	Sequence 3	UUAGGCUUCAGGUUCGUAG	5	miRNA	AGCUUUUGGCUCGGGUUAUA	0.391	20	45	0.333	-0.455	0.15	0.15	0.3	0.4	Ophiophagus hannah	https://mirbase.org/mature/MIMAT0036820
**45**	Sequence 3	UUAGGCUUCAGGUUCGUAG	6	siRNA	UUGGCUGUAGGACUCGUUG	0.388	19	52.63	0.4	-0.556	0.105	0.158	0.368	0.368	Homo Sapiens	
**46**	Sequence 3	UUAGGCUUCAGGUUCGUAG	7	siRNA	UCUAGGAGCAUUUAGGUUC	0.388	19	42.11	0.25	-0.273	0.211	0.158	0.263	0.368	Homo Sapiens	
**47**	Sequence 3	UUAGGCUUCAGGUUCGUAG	8	siRNA	UAAGGGUUGGAGUCUUCAC	0.388	19	47.37	0.333	-0.2	0.211	0.158	0.316	0.316	Homo Sapiens	
**48**	Sequence 3	UUAGGCUUCAGGUUCGUAG	9	siRNA	UAGUUGCAGCUCGGAGUUC	0.387	19	52.63	0.2	-0.333	0.158	0.211	0.316	0.316	Homo Sapiens	
**49**	Sequence 3	UUAGGCUUCAGGUUCGUAG	10	siRNA	UUAACGUCGGGUUUGUAGC	0.385	19	47.37	0.333	-0.4	0.158	0.158	0.316	0.368	Homo Sapiens	
**50**	Sequence 3	UUAGGCUUCAGGUUCGUAG	11	siRNA	AUGGCGUCUAGUUCUGGAG	0.381	19	52.63	0.4	-0.333	0.158	0.158	0.368	0.316	Homo Sapiens	
**51**	Sequence 3	UUAGGCUUCAGGUUCGUAG	12	siRNA	UAAGGUUCUUGCUGGGUCG	0.38	19	52.63	0.4	-0.556	0.105	0.158	0.368	0.368	Homo Sapiens	
**52**	Sequence 3	UUAGGCUUCAGGUUCGUAG	13	siRNA	UUUGGCUCCAGGUUUGACU	0.378	19	47.37	0.111	-0.6	0.105	0.211	0.263	0.421	Homo Sapiens	
**53**	Sequence 3	UUAGGCUUCAGGUUCGUAG	14	siRNA	AGAUUCUCAGGUUUAGUCC	0.377	19	42.11	0	-0.273	0.211	0.211	0.211	0.368	Homo Sapiens	
**54**	Sequence 3	UUAGGCUUCAGGUUCGUAG	15	siRNA	UGGAGCUUGGAUUUCGUCC	0.377	19	52.63	0.2	-0.556	0.105	0.211	0.316	0.368	Homo Sapiens	
**55**	Sequence 3	UUAGGCUUCAGGUUCGUAG	16	siRNA	UCGUACUUGAGGUCGUUGG	0.376	19	52.63	0.4	-0.556	0.105	0.158	0.368	0.368	Homo Sapiens	
**56**	Sequence 3	UUAGGCUUCAGGUUCGUAG	17	siRNA	AGGCUCGUGUUCUAUUUGG	0.375	19	47.37	0.333	-0.6	0.105	0.158	0.316	0.421	Homo Sapiens	
**57**	Sequence 3	UUAGGCUUCAGGUUCGUAG	18	siRNA	UCUUGUUAGAGGUUAGUCG	0.374	19	42.11	0.5	-0.455	0.158	0.105	0.316	0.421	Homo Sapiens	
**58**	Sequence 3	UUAGGCUUCAGGUUCGUAG	19	siRNA	UAGUUGGAGCGCUUUGUCA	0.372	19	47.37	0.333	-0.4	0.158	0.158	0.316	0.368	Homo Sapiens	
**59**	Sequence 3	UUAGGCUUCAGGUUCGUAG	20	siRNA	UAGGUCUUGGUCUCAGUCG	0.372	19	52.63	0.2	-0.556	0.105	0.211	0.316	0.368	Homo Sapiens	
**60**	Sequence 4	UUCUCAGCCACUGUUGCCA	1	siRNA	UUAUGCCCAGCUGCUCCAG	0.452	19	57.89	-0.273	-0.25	0.158	0.368	0.211	0.263	Homo Sapiens	
**61**	Sequence 4	UUCUCAGCCACUGUUGCCA	2	siRNA	UUUGUCACCAUAGCCCUGC	0.445	19	52.63	-0.4	-0.333	0.158	0.368	0.158	0.316	Homo Sapiens	
**62**	Sequence 4	UUCUCAGCCACUGUUGCCA	3	siRNA	UCAUUCACCAGUGCUAUGA	0.444	19	42.11	-0.25	-0.091	0.263	0.263	0.158	0.316	Homo Sapiens	
**63**	Sequence 4	UUCUCAGCCACUGUUGCCA	4	siRNA	UACUCUUCAGACGCUGCCA	0.443	19	52.63	-0.4	-0.111	0.211	0.368	0.158	0.263	Homo Sapiens	
**64**	Sequence 4	UUCUCAGCCACUGUUGCCA	5	miRNA	UCCCACUUGAUCACUGCUGCUAC	0.439	23	52.2	-0.5	-0.273	0.174	0.391	0.13	0.304	Petromyzon marinus	https://mirbase.org/mature/MIMAT0019584
**65**	Sequence 4	UUCUCAGCCACUGUUGCCA	6	siRNA	UCCAUAGCUUCCACUGUGC	0.432	19	52.63	-0.4	-0.333	0.158	0.368	0.158	0.316	Homo Sapiens	
**66**	Sequence 4	UUCUCAGCCACUGUUGCCA	7	miRNA	AUUUCACUCUCAGCAGGCUCC	0.43	21	52.4	-0.455	-0.2	0.19	0.381	0.143	0.286	Gallus gallus	https://mirbase.org/mature/MIMAT0049895
**67**	Sequence 4	UUCUCAGCCACUGUUGCCA	8	siRNA	CUCAUCUCCACGUAUGCUG	0.425	19	52.63	-0.4	-0.333	0.158	0.368	0.158	0.316	Homo Sapiens	
**68**	Sequence 4	UUCUCAGCCACUGUUGCCA	9	siRNA	UUUCAUCAGACCCUACUGC	0.421	19	47.37	-0.556	-0.2	0.211	0.368	0.105	0.316	Homo Sapiens	
**69**	Sequence 4	UUCUCAGCCACUGUUGCCA	10	siRNA	UAUUCUUCAGCCCAUGCAC	0.419	19	47.37	-0.556	-0.2	0.211	0.368	0.105	0.316	Homo Sapiens	
**70**	Sequence 4	UUCUCAGCCACUGUUGCCA	11	siRNA	UUAUCUACCACUUGCUCAG	0.418	19	42.11	-0.5	-0.273	0.211	0.316	0.105	0.368	Homo Sapiens	
**71**	Sequence 4	UUCUCAGCCACUGUUGCCA	12	siRNA	UGUUGCUCCACAGCCUUUC	0.418	19	52.63	-0.4	-0.556	0.105	0.368	0.158	0.368	Homo Sapiens	
**72**	Sequence 4	UUCUCAGCCACUGUUGCCA	13	miRNA	UGCAUCACAGCCUUUGGCCCUAG	0.418	23	56.5	-0.231	-0.2	0.174	0.348	0.217	0.261	Homo sapiens	https://mirbase.org/mature/MIMAT0028122
**73**	Sequence 4	UUCUCAGCCACUGUUGCCA	14	siRNA	UUCCUUGCCACCAGUGCUC	0.417	19	57.89	-0.455	-0.5	0.105	0.421	0.158	0.316	Homo Sapiens	
**74**	Sequence 4	UUCUCAGCCACUGUUGCCA	15	siRNA	UCGCUCUCCACUGUGCCAG	0.417	19	63.16	-0.333	-0.429	0.105	0.421	0.211	0.263	Homo Sapiens	
**75**	Sequence 4	UUCUCAGCCACUGUUGCCA	16	miRNA	CCUUAUCAUUUCCGCUGUCCAGA	0.417	23	47.8	-0.455	-0.333	0.174	0.348	0.13	0.348	Tetranychus urticae	https://mirbase.org/mature/MIMAT0023072
**76**	Sequence 4	UUCUCAGCCACUGUUGCCA	17	miRNA	UUAGCCUCACCCAGUUUUCCACG	0.417	23	52.2	-0.5	-0.273	0.174	0.391	0.13	0.304	Caenorhabditis elegans	https://mirbase.org/mature/MIMAT0032828
**77**	Sequence 4	UUCUCAGCCACUGUUGCCA	18	miRNA	UUCCCAGCUUGUGCCACUGAAA	0.417	22	50	-0.273	-0.091	0.227	0.318	0.182	0.273	Symbiodinium microadriaticum	https://mirbase.org/mature/MIMAT0050486
**78**	Sequence 4	UUCUCAGCCACUGUUGCCA	19	siRNA	UACUUCUCAGCCAGUGUGC	0.413	19	52.63	-0.2	-0.333	0.158	0.316	0.211	0.316	Homo Sapiens	
**79**	Sequence 4	UUCUCAGCCACUGUUGCCA	20	siRNA	AUUUCCCAUGCUCUGGUCA	0.413	19	47.37	-0.333	-0.4	0.158	0.316	0.158	0.368	Homo Sapiens	

Table S7. Seed-Based Matching RNA Sequences with Corresponding Seed and Sequence containing seed.

	Sequence ID	siRNA Sequence	Type	Sequence	Length	GC %	GC skew	AT/AU Skew	MNC A	MNC C	MNC G	MNC U	Species	Link
0	Sequence 1	UGUCACUUGCUCUUUGGUC	siRNA	AUGCGCGUCACUUCCUUCU	19	52.63	-0.4	-0.556	0.105	0.368	0.158	0.368	Homo Sapiens	
1	Sequence 1	UGUCACUUGCUCUUUGGUC	siRNA	GUUGGAGUCACUUGAUAUC	19	42.11	0.25	-0.273	0.211	0.158	0.263	0.368	Homo Sapiens	
2	Sequence 1	UGUCACUUGCUCUUUGGUC	siRNA	AGUCUCGUCACUUCUGAAC	19	47.37	-0.333	-0.2	0.211	0.316	0.158	0.316	Homo Sapiens	
3	Sequence 1	UGUCACUUGCUCUUUGGUC	siRNA	UUCUCCGUCACUUCAAUCC	19	47.37	-0.778	-0.4	0.158	0.421	0.053	0.368	Homo Sapiens	
4	Sequence 1	UGUCACUUGCUCUUUGGUC	siRNA	UCACGCUGGGUCACUUUGC	19	57.89	-0.091	-0.5	0.105	0.316	0.263	0.316	Homo Sapiens	
5	Sequence 1	UGUCACUUGCUCUUUGGUC	siRNA	ACAUCCCACAGUCACUUCC	19	52.63	-0.8	0.111	0.263	0.474	0.053	0.211	Homo Sapiens	
6	Sequence 1	UGUCACUUGCUCUUUGGUC	siRNA	GAUAAUGUUGGUCACUUGC	19	42.11	0.25	-0.273	0.211	0.158	0.263	0.368	Homo Sapiens	
7	Sequence 1	UGUCACUUGCUCUUUGGUC	siRNA	UACGUCACUUCUCAGGCCU	19	52.63	-0.4	-0.333	0.158	0.368	0.158	0.316	Homo Sapiens	
8	Sequence 1	UGUCACUUGCUCUUUGGUC	siRNA	ACGGCCGUCACUUUCUUGC	19	57.89	-0.273	-0.5	0.105	0.368	0.211	0.316	Homo Sapiens	
9	Sequence 1	UGUCACUUGCUCUUUGGUC	siRNA	AAUAUGGUCACUUCUUCUC	19	36.84	-0.429	-0.333	0.211	0.263	0.105	0.421	Homo Sapiens	
10	Sequence 1	UGUCACUUGCUCUUUGGUC	siRNA	AUACGGUGUCACUUCUUCG	19	47.37	-0.111	-0.4	0.158	0.263	0.211	0.368	Homo Sapiens	
11	Sequence 1	UGUCACUUGCUCUUUGGUC	siRNA	GAUGGCGUCACUUUCCUGC	19	57.89	-0.091	-0.5	0.105	0.316	0.263	0.316	Homo Sapiens	
12	Sequence 1	UGUCACUUGCUCUUUGGUC	siRNA	GUAUACGUCACUUUCUCCC	19	47.37	-0.556	-0.4	0.158	0.368	0.105	0.368	Homo Sapiens	
13	Sequence 1	UGUCACUUGCUCUUUGGUC	siRNA	UUACGGUGUUGUCACUUGU	19	42.11	0.25	-0.636	0.105	0.158	0.263	0.474	Homo Sapiens	
14	Sequence 1	UGUCACUUGCUCUUUGGUC	siRNA	CACUGGACAGGGUCACUUG	19	57.89	0.091	0	0.211	0.263	0.316	0.211	Homo Sapiens	
15	Sequence 1	UGUCACUUGCUCUUUGGUC	siRNA	UAUACGUAUUGUCACUUGG	19	36.84	0.143	-0.333	0.211	0.158	0.211	0.421	Homo Sapiens	
16	Sequence 1	UGUCACUUGCUCUUUGGUC	siRNA	GCACUGGUCACUUCUUGGG	19	57.89	0.091	-0.5	0.105	0.263	0.316	0.316	Homo Sapiens	
17	Sequence 1	UGUCACUUGCUCUUUGGUC	siRNA	UCAGUCGGGUCACUUGCUC	19	57.89	-0.091	-0.5	0.105	0.316	0.263	0.316	Homo Sapiens	
18	Sequence 1	UGUCACUUGCUCUUUGGUC	siRNA	GUUCGUUGUCACUUUAUUG	19	36.84	0.143	-0.667	0.105	0.158	0.211	0.526	Homo Sapiens	
19	Sequence 1	UGUCACUUGCUCUUUGGUC	siRNA	UUUUGUCAUAGGUCACUUC	19	36.84	-0.143	-0.5	0.158	0.211	0.158	0.474	Homo Sapiens	
20	Sequence 1	UGUCACUUGCUCUUUGGUC	siRNA	AAUCUCUAGGUCACUUUCU	19	36.84	-0.429	-0.333	0.211	0.263	0.105	0.421	Homo Sapiens	
21	Sequence 1	UGUCACUUGCUCUUUGGUC	siRNA	UCAUACACAUUGUCACUUC	19	36.84	-0.714	-0.167	0.263	0.316	0.053	0.368	Homo Sapiens	
22	Sequence 1	UGUCACUUGCUCUUUGGUC	siRNA	UUAAUGCUCUGGUCACUUC	19	42.11	-0.25	-0.455	0.158	0.263	0.158	0.421	Homo Sapiens	
23	Sequence 1	UGUCACUUGCUCUUUGGUC	siRNA	GAAUUGGUCACUUUCUCGC	19	47.37	-0.111	-0.4	0.158	0.263	0.211	0.368	Homo Sapiens	
24	Sequence 1	UGUCACUUGCUCUUUGGUC	siRNA	UCGGUUGUCACUUUCUUUC	19	42.11	-0.25	-0.818	0.053	0.263	0.158	0.526	Homo Sapiens	
25	Sequence 1	UGUCACUUGCUCUUUGGUC	siRNA	UUCGUAGUCACUUGUAUGG	19	42.11	0.25	-0.455	0.158	0.158	0.263	0.421	Homo Sapiens	
26	Sequence 1	UGUCACUUGCUCUUUGGUC	siRNA	UCCGCGGUCACUUCUGCAC	19	63.16	-0.333	-0.429	0.105	0.421	0.211	0.263	Homo Sapiens	
27	Sequence 1	UGUCACUUGCUCUUUGGUC	siRNA	GCACACGUCACUUCUCUCC	19	57.89	-0.636	-0.25	0.158	0.474	0.105	0.263	Homo Sapiens	
28	Sequence 1	UGUCACUUGCUCUUUGGUC	siRNA	UAAUAAUCGUCACUUAACC	19	31.58	-0.667	0.077	0.368	0.263	0.053	0.316	Homo Sapiens	
29	Sequence 1	UGUCACUUGCUCUUUGGUC	siRNA	GACGUCACUUGCACUGUUC	19	52.63	-0.2	-0.333	0.158	0.316	0.211	0.316	Homo Sapiens	
30	Sequence 1	UGUCACUUGCUCUUUGGUC	siRNA	UUGCCCGGUCACUUGGCUG	19	63.16	0	-0.714	0.053	0.316	0.316	0.316	Homo Sapiens	
31	Sequence 1	UGUCACUUGCUCUUUGGUC	siRNA	UAAUACGUCACUUGAUCUU	19	31.58	-0.333	-0.231	0.263	0.211	0.105	0.421	Homo Sapiens	
32	Sequence 1	UGUCACUUGCUCUUUGGUC	siRNA	UCUCACUAUUGUCACUUUA	19	31.58	-0.667	-0.385	0.211	0.263	0.053	0.474	Homo Sapiens	
33	Sequence 1	UGUCACUUGCUCUUUGGUC	siRNA	UUAUCCUAGUCACUUCAUC	19	36.84	-0.714	-0.333	0.211	0.316	0.053	0.421	Homo Sapiens	
34	Sequence 1	UGUCACUUGCUCUUUGGUC	siRNA	UUUCGGUCACUUUAUUACA	19	31.58	-0.333	-0.385	0.211	0.211	0.105	0.474	Homo Sapiens	
35	Sequence 1	UGUCACUUGCUCUUUGGUC	siRNA	AAGGGUAGUCACUUGUUGG	19	47.37	0.556	-0.2	0.211	0.105	0.368	0.316	Homo Sapiens	
36	Sequence 1	UGUCACUUGCUCUUUGGUC	siRNA	UCGACCUUGUCACUUCCUG	19	52.63	-0.4	-0.556	0.105	0.368	0.158	0.368	Homo Sapiens	
37	Sequence 1	UGUCACUUGCUCUUUGGUC	siRNA	UAGACAGUCACUUCGGUCC	19	52.63	-0.2	-0.111	0.211	0.316	0.211	0.263	Homo Sapiens	
38	Sequence 1	UGUCACUUGCUCUUUGGUC	siRNA	UAAUGUUCAUAGUCACUUC	19	31.58	-0.333	-0.231	0.263	0.211	0.105	0.421	Homo Sapiens	
39	Sequence 1	UGUCACUUGCUCUUUGGUC	siRNA	UUGCAGUCAUGGUCACUUC	19	47.37	-0.111	-0.4	0.158	0.263	0.211	0.368	Homo Sapiens	
40	Sequence 1	UGUCACUUGCUCUUUGGUC	siRNA	AAUGCCAGUCACUUAGUAC	19	42.11	-0.25	0.091	0.316	0.263	0.158	0.263	Homo Sapiens	
41	Sequence 1	UGUCACUUGCUCUUUGGUC	siRNA	UUCCGAGUCACUUUGUCGC	19	52.63	-0.2	-0.556	0.105	0.316	0.211	0.368	Homo Sapiens	
42	Sequence 1	UGUCACUUGCUCUUUGGUC	siRNA	AAUGCGUCACUUUCAUCAC	19	42.11	-0.5	-0.091	0.263	0.316	0.105	0.316	Homo Sapiens	
43	Sequence 1	UGUCACUUGCUCUUUGGUC	siRNA	GAUAUGGUCACUUCUGGUG	19	47.37	0.333	-0.4	0.158	0.158	0.316	0.368	Homo Sapiens	
44	Sequence 1	UGUCACUUGCUCUUUGGUC	siRNA	AGUAACUCCAGUCACUUCC	19	47.37	-0.556	0	0.263	0.368	0.105	0.263	Homo Sapiens	
45	Sequence 1	UGUCACUUGCUCUUUGGUC	siRNA	UGAGAGCAGGGUCACUUUA	19	47.37	0.333	0	0.263	0.158	0.316	0.263	Homo Sapiens	
46	Sequence 1	UGUCACUUGCUCUUUGGUC	siRNA	UAGUCGUCACUUCCUUGGC	19	52.63	-0.2	-0.556	0.105	0.316	0.211	0.368	Homo Sapiens	
47	Sequence 1	UGUCACUUGCUCUUUGGUC	siRNA	UCACACUGUCACUUCGUGG	19	52.63	-0.2	-0.333	0.158	0.316	0.211	0.316	Homo Sapiens	
48	Sequence 1	UGUCACUUGCUCUUUGGUC	siRNA	AUUCGAUCAUGUCACUUGC	19	42.11	-0.25	-0.273	0.211	0.263	0.158	0.368	Homo Sapiens	
49	Sequence 1	UGUCACUUGCUCUUUGGUC	siRNA	UACGACUAGUCACUUGUGC	19	47.37	-0.111	-0.2	0.211	0.263	0.211	0.316	Homo Sapiens	
50	Sequence 1	UGUCACUUGCUCUUUGGUC	siRNA	AACUCUGUCACUUCUGUGC	19	47.37	-0.333	-0.4	0.158	0.316	0.158	0.368	Homo Sapiens	
51	Sequence 1	UGUCACUUGCUCUUUGGUC	siRNA	GAUCUGAAGAGUCACUUGG	19	47.37	0.333	0	0.263	0.158	0.316	0.263	Homo Sapiens	
52	Sequence 1	UGUCACUUGCUCUUUGGUC	siRNA	UUAACUUCCUGUCACUUCC	19	42.11	-0.75	-0.455	0.158	0.368	0.053	0.421	Homo Sapiens	
53	Sequence 1	UGUCACUUGCUCUUUGGUC	siRNA	UAAACGUCUGUCACUUUGG	19	42.11	0	-0.273	0.211	0.211	0.211	0.368	Homo Sapiens	
54	Sequence 1	UGUCACUUGCUCUUUGGUC	siRNA	AAGCCCGAGGUCACUUUGG	19	57.89	0.091	0	0.211	0.263	0.316	0.211	Homo Sapiens	
55	Sequence 1	UGUCACUUGCUCUUUGGUC	siRNA	UACCGCAGCGUCACUUAAG	19	52.63	-0.2	0.111	0.263	0.316	0.211	0.211	Homo Sapiens	
56	Sequence 1	UGUCACUUGCUCUUUGGUC	siRNA	UCGAUGGUCACUUCUUCAU	19	42.11	-0.25	-0.455	0.158	0.263	0.158	0.421	Homo Sapiens	
57	Sequence 1	UGUCACUUGCUCUUUGGUC	siRNA	GCGCUUGUCACUUUCGUUC	19	52.63	-0.2	-0.778	0.053	0.316	0.211	0.421	Homo Sapiens	
58	Sequence 1	UGUCACUUGCUCUUUGGUC	siRNA	UCCCGCUGGUCACUUUCGC	19	63.16	-0.333	-0.714	0.053	0.421	0.211	0.316	Homo Sapiens	
59	Sequence 1	UGUCACUUGCUCUUUGGUC	siRNA	AGACACGCAGUCACUUUCC	19	52.63	-0.4	0.111	0.263	0.368	0.158	0.211	Homo Sapiens	
60	Sequence 1	UGUCACUUGCUCUUUGGUC	siRNA	AAGCUUGUCACUUCUGCCG	19	52.63	-0.2	-0.333	0.158	0.316	0.211	0.316	Homo Sapiens	
61	Sequence 1	UGUCACUUGCUCUUUGGUC	siRNA	CCCUGGUCACUUGUUGUCC	19	57.89	-0.273	-0.75	0.053	0.368	0.211	0.368	Homo Sapiens	
62	Sequence 1	UGUCACUUGCUCUUUGGUC	siRNA	AUACGAUGUCACUUAGUCC	19	42.11	-0.25	-0.091	0.263	0.263	0.158	0.316	Homo Sapiens	
63	Sequence 1	UGUCACUUGCUCUUUGGUC	siRNA	UUACGAGUCACUUCAGGCC	19	52.63	-0.2	-0.111	0.211	0.316	0.211	0.263	Homo Sapiens	
64	Sequence 1	UGUCACUUGCUCUUUGGUC	siRNA	AUCGAGGUCACUUCGUCUC	19	52.63	-0.2	-0.333	0.158	0.316	0.211	0.316	Homo Sapiens	
65	Sequence 1	UGUCACUUGCUCUUUGGUC	siRNA	UAAUGUCAGCGUCACUUGG	19	47.37	0.111	-0.2	0.211	0.211	0.263	0.316	Homo Sapiens	
66	Sequence 1	UGUCACUUGCUCUUUGGUC	siRNA	UGAGACUACGGUCACUUGG	19	52.63	0.2	-0.111	0.211	0.211	0.316	0.263	Homo Sapiens	
67	Sequence 1	UGUCACUUGCUCUUUGGUC	siRNA	UUUCCCGUCACUUGUGGAG	19	52.63	0	-0.556	0.105	0.263	0.263	0.368	Homo Sapiens	
68	Sequence 1	UGUCACUUGCUCUUUGGUC	siRNA	AACUGCGUCACUUCCACUC	19	52.63	-0.6	-0.111	0.211	0.421	0.105	0.263	Homo Sapiens	
69	Sequence 1	UGUCACUUGCUCUUUGGUC	siRNA	AUCCGGAACAGGUCACUUG	19	52.63	0	0.111	0.263	0.263	0.263	0.211	Homo Sapiens	
70	Sequence 1	UGUCACUUGCUCUUUGGUC	siRNA	UCAACGAGUCACUUUUAGC	19	42.11	-0.25	-0.091	0.263	0.263	0.158	0.316	Homo Sapiens	
71	Sequence 1	UGUCACUUGCUCUUUGGUC	siRNA	AAUCACGUCACUUUGCCCA	19	47.37	-0.556	0	0.263	0.368	0.105	0.263	Homo Sapiens	
72	Sequence 1	UGUCACUUGCUCUUUGGUC	siRNA	AUUUGGGUCACUUCGGUGG	19	52.63	0.4	-0.556	0.105	0.158	0.368	0.368	Homo Sapiens	
73	Sequence 1	UGUCACUUGCUCUUUGGUC	siRNA	UAGUCACUUGCCUAUCCUC	19	47.37	-0.556	-0.4	0.158	0.368	0.105	0.368	Homo Sapiens	
74	Sequence 1	UGUCACUUGCUCUUUGGUC	siRNA	UAACGCAGAUGUCACUUCU	19	42.11	-0.25	-0.091	0.263	0.263	0.158	0.316	Homo Sapiens	
75	Sequence 1	UGUCACUUGCUCUUUGGUC	siRNA	AAUCACAUCGUCACUUAUG	19	36.84	-0.429	0	0.316	0.263	0.105	0.316	Homo Sapiens	
76	Sequence 1	UGUCACUUGCUCUUUGGUC	siRNA	UGCCGUAUCAGUCACUUUC	19	47.37	-0.333	-0.4	0.158	0.316	0.158	0.368	Homo Sapiens	
77	Sequence 1	UGUCACUUGCUCUUUGGUC	siRNA	AGAAUGUACAUGUCACUUC	19	36.84	-0.143	0	0.316	0.211	0.158	0.316	Homo Sapiens	
78	Sequence 1	UGUCACUUGCUCUUUGGUC	siRNA	AAUUCCGUCACUUCAGAGG	19	47.37	-0.111	0	0.263	0.263	0.211	0.263	Homo Sapiens	
79	Sequence 1	UGUCACUUGCUCUUUGGUC	siRNA	UAUAACGAGGGUCACUUUC	19	42.11	0	-0.091	0.263	0.211	0.211	0.316	Homo Sapiens	
80	Sequence 1	UGUCACUUGCUCUUUGGUC	siRNA	UAUUUCAGCACCGUCACUU	19	42.11	-0.5	-0.273	0.211	0.316	0.105	0.368	Homo Sapiens	
81	Sequence 1	UGUCACUUGCUCUUUGGUC	siRNA	AUAAUCUGAUGGUCACUUG	19	36.84	0.143	-0.167	0.263	0.158	0.211	0.368	Homo Sapiens	
82	Sequence 1	UGUCACUUGCUCUUUGGUC	siRNA	UUUCCGUCACUUUCCCUCU	19	47.37	-0.778	-0.8	0.053	0.421	0.053	0.474	Homo Sapiens	
83	Sequence 1	UGUCACUUGCUCUUUGGUC	siRNA	AUGAACUUUGUCACUUCCG	19	42.11	-0.25	-0.273	0.211	0.263	0.158	0.368	Homo Sapiens	
84	Sequence 1	UGUCACUUGCUCUUUGGUC	siRNA	GAAUUCCUGUGUCACUUGC	19	47.37	-0.111	-0.4	0.158	0.263	0.211	0.368	Homo Sapiens	
85	Sequence 1	UGUCACUUGCUCUUUGGUC	siRNA	UAUACUGUGUCACUUUGUG	19	36.84	0.143	-0.5	0.158	0.158	0.211	0.474	Homo Sapiens	
86	Sequence 1	UGUCACUUGCUCUUUGGUC	siRNA	UAGCCAGUCACUUCCUCCC	19	57.89	-0.636	-0.25	0.158	0.474	0.105	0.263	Homo Sapiens	
87	Sequence 1	UGUCACUUGCUCUUUGGUC	siRNA	UCCACGAGGUCACUUGUCC	19	57.89	-0.273	-0.25	0.158	0.368	0.211	0.263	Homo Sapiens	
88	Sequence 1	UGUCACUUGCUCUUUGGUC	siRNA	AGUCACUUGCCUGUUUCGC	19	52.63	-0.2	-0.556	0.105	0.316	0.211	0.368	Homo Sapiens	
89	Sequence 1	UGUCACUUGCUCUUUGGUC	siRNA	GUUGAUGUCACUUAGGUCC	19	47.37	0.111	-0.4	0.158	0.211	0.263	0.368	Homo Sapiens	
90	Sequence 1	UGUCACUUGCUCUUUGGUC	siRNA	UUAGUCACUUACGAAUUCC	19	36.84	-0.429	-0.167	0.263	0.263	0.105	0.368	Homo Sapiens	
91	Sequence 1	UGUCACUUGCUCUUUGGUC	siRNA	AGCGUCACUUCUUCUUGGC	19	52.63	-0.2	-0.556	0.105	0.316	0.211	0.368	Homo Sapiens	
92	Sequence 1	UGUCACUUGCUCUUUGGUC	siRNA	AGACGGGUCACUUCGUUCC	19	57.89	-0.091	-0.25	0.158	0.316	0.263	0.263	Homo Sapiens	
93	Sequence 1	UGUCACUUGCUCUUUGGUC	siRNA	UCAGUCACUUUGAUGCUCC	19	47.37	-0.333	-0.4	0.158	0.316	0.158	0.368	Homo Sapiens	
94	Sequence 1	UGUCACUUGCUCUUUGGUC	siRNA	AACUUUAUUGGUCACUUCC	19	36.84	-0.429	-0.333	0.211	0.263	0.105	0.421	Homo Sapiens	
95	Sequence 1	UGUCACUUGCUCUUUGGUC	siRNA	AAUUGAACUGGUCACUUGG	19	42.11	0.25	-0.091	0.263	0.158	0.263	0.316	Homo Sapiens	
96	Sequence 1	UGUCACUUGCUCUUUGGUC	siRNA	AUACCGUCACUUUGUAAUG	19	36.84	-0.143	-0.167	0.263	0.211	0.158	0.368	Homo Sapiens	
97	Sequence 1	UGUCACUUGCUCUUUGGUC	siRNA	GAUUACGUCACUUUCUUCC	19	42.11	-0.5	-0.455	0.158	0.316	0.105	0.421	Homo Sapiens	
98	Sequence 1	UGUCACUUGCUCUUUGGUC	siRNA	ACUGUCACUUGCUCUUGCC	19	52.63	-0.4	-0.556	0.105	0.368	0.158	0.368	Homo Sapiens	
99	Sequence 1	UGUCACUUGCUCUUUGGUC	siRNA	AGAACCUGUCACUUGAUCC	19	47.37	-0.333	0	0.263	0.316	0.158	0.263	Homo Sapiens	
100	Sequence 1	UGUCACUUGCUCUUUGGUC	siRNA	AGUCACUUCAGGUCCUUAC	19	47.37	-0.333	-0.2	0.211	0.316	0.158	0.316	Homo Sapiens	
101	Sequence 1	UGUCACUUGCUCUUUGGUC	siRNA	CACUGAAGUGCGGUCACUU	19	52.63	0	-0.111	0.211	0.263	0.263	0.263	Homo Sapiens	
102	Sequence 1	UGUCACUUGCUCUUUGGUC	siRNA	AGUGCGGUCACUUCUCCGG	19	63.16	0	-0.429	0.105	0.316	0.316	0.263	Homo Sapiens	
103	Sequence 1	UGUCACUUGCUCUUUGGUC	siRNA	GGUCACUUCUCCGGCUUCC	19	63.16	-0.333	-0.714	0.053	0.421	0.211	0.316	Homo Sapiens	
104	Sequence 1	UGUCACUUGCUCUUUGGUC	siRNA	ACUGAAGUGCGGUCACUUC	19	52.63	0	-0.111	0.211	0.263	0.263	0.263	Homo Sapiens	
105	Sequence 1	UGUCACUUGCUCUUUGGUC	siRNA	CUGAAGUGCGGUCACUUCU	19	52.63	0	-0.333	0.158	0.263	0.263	0.316	Homo Sapiens	
106	Sequence 1	UGUCACUUGCUCUUUGGUC	miRNA	UGUGUCACUUGACGACCACUGU	22	50	-0.091	-0.273	0.182	0.273	0.227	0.318	Macaca mulatta	https://mirbase.org/mature/MIMAT0006454
107	Sequence 1	UGUCACUUGCUCUUUGGUC	miRNA	AAGUGGUCACUUGAGUUGCAGU	22	45.45	0.4	-0.167	0.227	0.136	0.318	0.318	Ornithorhynchus anatinus	https://mirbase.org/mature/MIMAT0007068
108	Sequence 1	UGUCACUUGCUCUUUGGUC	miRNA	AGGUGGUCACUUGAGUUGCAGU	22	50	0.455	-0.273	0.182	0.136	0.364	0.318	Ornithorhynchus anatinus	https://mirbase.org/mature/MIMAT0007084
109	Sequence 1	UGUCACUUGCUCUUUGGUC	miRNA	CAGGGGUCACUUUUUUUGUUU	20	40	0.25	-0.667	0.1	0.15	0.25	0.5	Gallus gallus	https://mirbase.org/mature/MIMAT0007668
110	Sequence 1	UGUCACUUGCUCUUUGGUC	miRNA	UUGGUCACUUGCUUUAUUCACUC	23	39.13	-0.333	-0.571	0.13	0.261	0.13	0.478	Schmidtea mediterranea	https://mirbase.org/mature/MIMAT0011263
111	Sequence 1	UGUCACUUGCUCUUUGGUC	miRNA	ACGGGAAUGGUCACUUACAACU	22	45.45	0	0.167	0.318	0.227	0.227	0.227	Bombyx mori	https://mirbase.org/mature/MIMAT0013627
112	Sequence 1	UGUCACUUGCUCUUUGGUC	miRNA	GGCUGUGUCACUUCGAGCCAGC	22	63.64	0	-0.25	0.136	0.318	0.318	0.227	Bombyx mori;Manduca sexta	https://mirbase.org/mature/MIMAT0015242;https://mirbase.org/mature/MIMAT0024447
113	Sequence 1	UGUCACUUGCUCUUUGGUC	miRNA	UAGUCACUUGGGAGACGUCCUGG	23	56.52	0.231	-0.2	0.174	0.217	0.348	0.261	Bombyx mori	https://mirbase.org/mature/MIMAT0015427
114	Sequence 1	UGUCACUUGCUCUUUGGUC	miRNA	GCGUCACUUGUGGUGUAGGCGA	22	59.09	0.385	-0.333	0.136	0.182	0.409	0.273	Schistosoma japonicum	https://mirbase.org/mature/MIMAT0016290
115	Sequence 1	UGUCACUUGCUCUUUGGUC	miRNA	GUCACUUCGCUUAAGUUUA	19	36.84	-0.143	-0.333	0.211	0.211	0.158	0.421	Ciona intestinalis	https://mirbase.org/mature/MIMAT0016445
116	Sequence 1	UGUCACUUGCUCUUUGGUC	miRNA	CCGGUUUAGUCACUUUCAC	19	47.37	-0.333	-0.4	0.158	0.316	0.158	0.368	Arabidopsis lyrata	https://mirbase.org/mature/MIMAT0017443
117	Sequence 1	UGUCACUUGCUCUUUGGUC	miRNA	GUCACUUUCACUGCAUUAAUC	21	38.1	-0.5	-0.231	0.238	0.286	0.095	0.381	Arabidopsis lyrata	https://mirbase.org/mature/MIMAT0017444
118	Sequence 1	UGUCACUUGCUCUUUGGUC	miRNA	UCUAAACUGGUCACUUCCACUC	22	45.45	-0.6	-0.167	0.227	0.364	0.091	0.318	Drosophila melanogaster	https://mirbase.org/mature/MIMAT0018056
119	Sequence 1	UGUCACUUGCUCUUUGGUC	miRNA	UGUCUAUACUCUGUCACUUUAC	22	36.36	-0.5	-0.429	0.182	0.273	0.091	0.455	Homo sapiens	https://mirbase.org/mature/MIMAT0019970
120	Sequence 1	UGUCACUUGCUCUUUGGUC	miRNA	GCUUGUCACUUCCAAUACAUGC	22	45.45	-0.4	-0.167	0.227	0.318	0.136	0.318	Branchiostoma floridae;Branchiostoma belcheri	https://mirbase.org/mature/MIMAT0020099;https://mirbase.org/mature/MIMAT0031687
121	Sequence 1	UGUCACUUGCUCUUUGGUC	miRNA	ACUGCUGAAAGUCACUUUAGG	21	42.86	0.111	0	0.286	0.19	0.238	0.286	Branchiostoma floridae	https://mirbase.org/mature/MIMAT0020469
122	Sequence 1	UGUCACUUGCUCUUUGGUC	miRNA	CCUGUCACUUCUAUAGGCCAAU	22	45.45	-0.4	-0.167	0.227	0.318	0.136	0.318	Taeniopygia guttata	https://mirbase.org/mature/MIMAT0027009
123	Sequence 1	UGUCACUUGCUCUUUGGUC	miRNA	ACUGUCACUUCUCUGCCCAUAG	22	50	-0.455	-0.273	0.182	0.364	0.136	0.318	Homo sapiens	https://mirbase.org/mature/MIMAT0027447
124	Sequence 1	UGUCACUUGCUCUUUGGUC	miRNA	GUCACUUCUCUUCCCACCACAG	22	54.55	-0.667	-0.2	0.182	0.455	0.091	0.273	Mus musculus	https://mirbase.org/mature/MIMAT0027735
125	Sequence 1	UGUCACUUGCUCUUUGGUC	miRNA	UGGGUGGAUAAGUCACUUUACUUU	24	37.5	0.333	-0.333	0.208	0.125	0.25	0.417	Monodelphis domestica	https://mirbase.org/mature/MIMAT0028864
126	Sequence 1	UGUCACUUGCUCUUUGGUC	miRNA	UGCGAUACGUCACUUCACCGAAAU	24	45.83	-0.273	0.077	0.292	0.292	0.167	0.25	Brachypodium distachyon	https://mirbase.org/mature/MIMAT0030219
127	Sequence 1	UGUCACUUGCUCUUUGGUC	miRNA	CUGGGUCACUUCUACGGGUCCUUC	24	58.33	-0.143	-0.6	0.083	0.333	0.25	0.333	Solanum tuberosum	https://mirbase.org/mature/MIMAT0031189
128	Sequence 1	UGUCACUUGCUCUUUGGUC	miRNA	UGUGUCACUUGUGGAUGUGUAGAGU	25	44	0.636	-0.429	0.16	0.08	0.36	0.4	Eptesicus fuscus	https://mirbase.org/mature/MIMAT0034847
129	Sequence 1	UGUCACUUGCUCUUUGGUC	miRNA	GUCACUUUCAAUGCGUUGAUC	21	42.86	-0.111	-0.333	0.19	0.238	0.19	0.381	Brassica rapa	https://mirbase.org/mature/MIMAT0035879
130	Sequence 1	UGUCACUUGCUCUUUGGUC	miRNA	CUGUCACUUCUGUAGGCCAAUA	22	45.45	-0.2	-0.167	0.227	0.273	0.182	0.318	Chrysemys picta	https://mirbase.org/mature/MIMAT0037842
131	Sequence 1	UGUCACUUGCUCUUUGGUC	miRNA	AAGGAGAAUCUUUGUCACUUAG	22	36.36	0.25	0	0.318	0.136	0.227	0.318	Pteropus alecto	https://mirbase.org/mature/MIMAT0040180
132	Sequence 1	UGUCACUUGCUCUUUGGUC	miRNA	UAAUGUCACUUUGACAGCAAACC	23	39.13	-0.333	0.143	0.348	0.261	0.13	0.261	Spodoptera frugiperda	https://mirbase.org/mature/MIMAT0041854
133	Sequence 1	UGUCACUUGCUCUUUGGUC	miRNA	AGUCACUUUGAUGAUUGUCAAACA	24	33.33	0	0	0.333	0.167	0.167	0.333	Solanum lycopersicum	https://mirbase.org/mature/MIMAT0042035
134	Sequence 1	UGUCACUUGCUCUUUGGUC	miRNA	CAAAGAUCUGCUGACGUCACUU	22	45.45	-0.2	0	0.273	0.273	0.182	0.273	Neolamprologus brichardi	https://mirbase.org/mature/MIMAT0042463
135	Sequence 1	UGUCACUUGCUCUUUGGUC	miRNA	AGAGAGCUCUAAUGUCACUUA	21	38.1	0	0.077	0.333	0.19	0.19	0.286	Oreochromis niloticus	https://mirbase.org/mature/MIMAT0043473
136	Sequence 1	UGUCACUUGCUCUUUGGUC	miRNA	AGCUAGAUACCUUCGUCACUUUGU	24	41.67	-0.2	-0.286	0.208	0.25	0.167	0.375	Lotus japonicus	https://mirbase.org/mature/MIMAT0043960
137	Sequence 1	UGUCACUUGCUCUUUGGUC	miRNA	UAAAACGUCACUUUAUUGCUCGU	23	34.78	-0.25	-0.2	0.261	0.217	0.13	0.391	Dinoponera quadriceps	https://mirbase.org/mature/MIMAT0045820
138	Sequence 1	UGUCACUUGCUCUUUGGUC	miRNA	UCGAAUCUGUCACUUCGGUAAUU	23	39.13	-0.111	-0.286	0.217	0.217	0.174	0.391	Parasteatoda tepidariorum	https://mirbase.org/mature/MIMAT0046178
139	Sequence 1	UGUCACUUGCUCUUUGGUC	miRNA	UCUGUCACUUCUGUAGGCCAACA	23	47.83	-0.273	-0.167	0.217	0.304	0.174	0.304	Cavia porcellus	https://mirbase.org/mature/MIMAT0047118
140	Sequence 1	UGUCACUUGCUCUUUGGUC	miRNA	AAGGAGAAUCUUUGUCACUUCAU	23	34.78	0	-0.067	0.304	0.174	0.174	0.348	Cavia porcellus	https://mirbase.org/mature/MIMAT0047388
141	Sequence 1	UGUCACUUGCUCUUUGGUC	miRNA	CAAGUCACUUGUCUACCAUUAGU	23	39.13	-0.333	-0.143	0.261	0.261	0.13	0.348	Dasypus novemcinctus	https://mirbase.org/mature/MIMAT0047781
142	Sequence 1	UGUCACUUGCUCUUUGGUC	miRNA	AAGGAGAAUCUUUGUCACUUGGU	23	39.13	0.333	-0.143	0.261	0.13	0.261	0.348	Dasypus novemcinctus	https://mirbase.org/mature/MIMAT0048015
143	Sequence 1	UGUCACUUGCUCUUUGGUC	miRNA	AAGGAGAAUCUUUGUCACUUAGU	23	34.78	0.25	-0.067	0.304	0.13	0.217	0.348	Oryctolagus cuniculus	https://mirbase.org/mature/MIMAT0048570
144	Sequence 1	UGUCACUUGCUCUUUGGUC	piRNA	UCUCAUCUGUCACUUCGAGUUGUUGGC	27	48.15	-0.077	-0.571	0.111	0.259	0.222	0.407	Homo sapiens	https://dashr2.lisanwanglab.org/get_bg_smrna.php?coord=chr1:161363764-161363790:pos&rnaID=piR-45558&dataSource=DASHR2_GEO_hg38
145	Sequence 1	UGUCACUUGCUCUUUGGUC	piRNA	UCAGUCACUUGGGGAAGUUCAGGAGGCUGC	30	56.67	0.294	-0.077	0.2	0.2	0.367	0.233	Homo sapiens	https://dashr2.lisanwanglab.org/get_bg_smrna.php?coord=chr10:28791752-28791781:neg&rnaID=piR-41775&dataSource=DASHR2_GEO_hg38
146	Sequence 1	UGUCACUUGCUCUUUGGUC	piRNA	UCAGUCACUUGGGGAAGUUCAGGAGGCUG	29	55.17	0.375	-0.077	0.207	0.172	0.379	0.241	Homo sapiens	https://dashr2.lisanwanglab.org/get_bg_smrna.php?coord=chr10:28791753-28791781:neg&rnaID=piR-41774&dataSource=DASHR2_GEO_hg38;https://dashr2.lisanwanglab.org/get_bg_smrna.php?coord=chr12:54819857-54819885:pos&rnaID=piR-41774&dataSource=DASHR2_GEO_hg38
147	Sequence 1	UGUCACUUGCUCUUUGGUC	piRNA	CAGGAUGUGCUUCACAUCAGUCACUUGGGGA	31	51.61	0.125	-0.067	0.226	0.226	0.29	0.258	Homo sapiens	https://dashr2.lisanwanglab.org/get_bg_smrna.php?coord=chr10:28791767-28791797:neg&rnaID=piR-32520&dataSource=DASHR2_GEO_hg38
148	Sequence 1	UGUCACUUGCUCUUUGGUC	piRNA	UGCAGGAUGUGCUUCACAUCAGUCACUUGGG	31	51.61	0.125	-0.2	0.194	0.226	0.29	0.29	Homo sapiens	https://dashr2.lisanwanglab.org/get_bg_smrna.php?coord=chr10:28791769-28791799:neg&rnaID=piR-50861&dataSource=DASHR2_GEO_hg38
149	Sequence 1	UGUCACUUGCUCUUUGGUC	piRNA	UGAGAGUUCAUGUCACUUAACGGAGC	26	46.15	0.167	0	0.269	0.192	0.269	0.269	Homo sapiens	https://dashr2.lisanwanglab.org/get_bg_smrna.php?coord=chr11:7020240-7020265:pos&rnaID=piR-48528&dataSource=DASHR2_GEO_hg38
150	Sequence 1	UGUCACUUGCUCUUUGGUC	piRNA	UAAUUGGUAAAGAAGCUGCAGUCACUUGU	29	37.93	0.273	0	0.31	0.138	0.241	0.31	Homo sapiens	https://dashr2.lisanwanglab.org/get_bg_smrna.php?coord=chr11:45688111-45688139:neg&rnaID=piR-37736&dataSource=DASHR2_GEO_hg38
151	Sequence 1	UGUCACUUGCUCUUUGGUC	piRNA	UGUCAGGUCACUUGAGUCGUUGAUGGA	27	48.15	0.385	-0.286	0.185	0.148	0.333	0.333	Homo sapiens	https://dashr2.lisanwanglab.org/get_bg_smrna.php?coord=chr11:45716595-45716621:neg&rnaID=piR-58083&dataSource=DASHR2_GEO_hg38
152	Sequence 1	UGUCACUUGCUCUUUGGUC	piRNA	GUCACUUUGCUCCUUGGGUAGUUGUGUUA	29	44.83	0.231	-0.625	0.103	0.172	0.276	0.448	Homo sapiens	https://dashr2.lisanwanglab.org/get_bg_smrna.php?coord=chr11:45718244-45718272:neg&rnaID=piR-36471&dataSource=DASHR2_GEO_hg38
153	Sequence 1	UGUCACUUGCUCUUUGGUC	piRNA	UGAGGUUGAAGAGGUGUCUCUGUGUCACUU	30	46.67	0.429	-0.375	0.167	0.133	0.333	0.367	Homo sapiens	https://dashr2.lisanwanglab.org/get_bg_smrna.php?coord=chr11:45718266-45718295:neg&rnaID=piR-49232&dataSource=DASHR2_GEO_hg38
154	Sequence 1	UGUCACUUGCUCUUUGGUC	piRNA	UGUCACUUCAAUGGCGUGCCAACCUCC	27	55.56	-0.333	-0.167	0.185	0.37	0.185	0.259	Homo sapiens	https://dashr2.lisanwanglab.org/get_bg_smrna.php?coord=chr14:88164413-88164439:pos&rnaID=piR-58056&dataSource=DASHR2_GEO_hg38
155	Sequence 1	UGUCACUUGCUCUUUGGUC	piRNA	GCCACAUUUGACUGUGUCACUUCUCUCUUU	30	43.33	-0.385	-0.529	0.133	0.3	0.133	0.433	Homo sapiens	https://dashr2.lisanwanglab.org/get_bg_smrna.php?coord=chr15:51295867-51295896:neg&rnaID=piR-35324&dataSource=DASHR2_GEO_hg38
156	Sequence 1	UGUCACUUGCUCUUUGGUC	piRNA	UGGUCUGGCCACAUUUGACUGUGUCACUUC	30	50	-0.067	-0.467	0.133	0.267	0.233	0.367	Homo sapiens	https://dashr2.lisanwanglab.org/get_bg_smrna.php?coord=chr15:51295874-51295903:neg&rnaID=piR-57157&dataSource=DASHR2_GEO_hg38
157	Sequence 1	UGUCACUUGCUCUUUGGUC	piRNA	UGAGGUGUCACUUGACGUGUGAGGAA	26	50	0.538	-0.077	0.231	0.115	0.385	0.269	Homo sapiens	https://dashr2.lisanwanglab.org/get_bg_smrna.php?coord=chr17:18005052-18005077:neg&rnaID=piR-49211&dataSource=DASHR2_GEO_hg38
158	Sequence 1	UGUCACUUGCUCUUUGGUC	piRNA	UGGCACAGGUCACUUGCACAUAGGUCUCGGA	31	54.84	0.059	0	0.226	0.258	0.29	0.226	Homo sapiens	https://dashr2.lisanwanglab.org/get_bg_smrna.php?coord=chr17:68126638-68126668:pos&rnaID=piR-54664&dataSource=DASHR2_GEO_hg38
159	Sequence 1	UGUCACUUGCUCUUUGGUC	piRNA	UGCUGCCAUCUGGUGGUGAUUUCGGUCACUU	31	51.61	0.125	-0.6	0.097	0.226	0.29	0.387	Homo sapiens	https://dashr2.lisanwanglab.org/get_bg_smrna.php?coord=chr17:78993319-78993349:neg&rnaID=piR-53089&dataSource=DASHR2_GEO_hg38
160	Sequence 1	UGUCACUUGCUCUUUGGUC	piRNA	UGGUCACUUGCCAGAACUAUUGGAUGAAUGUC	32	43.75	0.143	-0.111	0.25	0.188	0.25	0.313	Homo sapiens	https://dashr2.lisanwanglab.org/get_bg_smrna.php?coord=chr18:11662799-11662830:neg&rnaID=piR-57052&dataSource=DASHR2_GEO_hg38
161	Sequence 1	UGUCACUUGCUCUUUGGUC	piRNA	UGUGGUCACUUGCCAGAACUAUUGGAUGAAU	31	41.94	0.231	-0.111	0.258	0.161	0.258	0.323	Homo sapiens	https://dashr2.lisanwanglab.org/get_bg_smrna.php?coord=chr18:11662802-11662832:neg&rnaID=piR-59054&dataSource=DASHR2_GEO_hg38

	Sequence ID	siRNA Sequence	Type	Sequence	Length	GC %	GC skew	AT/AU Skew	MNC A	MNC C	MNC G	MNC U	Species	Link
162	Sequence 1	UGUCACUUGCUCUUUGGUC	piRNA	CUUGUGGUCACUUCCUUCCAGUACAGUC	28	50	-0.286	-0.429	0.143	0.321	0.179	0.357	Homo sapiens	https://dashr2.lisanwanglab.org/get_bg_smrna.php?coord=chr19:34838416-34838443:neg&rnaID=piR-34058&dataSource=DASHR2_GEO_hg38
163	Sequence 1	UGUCACUUGCUCUUUGGUC	piRNA	UGAGCAGGGAUUUCUGUCACUUGCGGCAGAAA	32	50	0.25	0	0.25	0.188	0.313	0.25	Homo sapiens	https://dashr2.lisanwanglab.org/get_bg_smrna.php?coord=chr22:37355185-37355216:neg&rnaID=piR-48705&dataSource=DASHR2_GEO_hg38
164	Sequence 1	UGUCACUUGCUCUUUGGUC	piRNA	UGGCGUCACUUGGGCCCAGGAGUUGAGUGG	30	63.33	0.368	-0.273	0.133	0.2	0.433	0.233	Homo sapiens	https://dashr2.lisanwanglab.org/get_bg_smrna.php?coord=chr22:37361418-37361447:neg&rnaID=piR-55171&dataSource=DASHR2_GEO_hg38
165	Sequence 1	UGUCACUUGCUCUUUGGUC	piRNA	CUGUGUCACUUUACAGGAUUGGUUGGU	27	44.44	0.333	-0.467	0.148	0.148	0.296	0.407	Homo sapiens	https://dashr2.lisanwanglab.org/get_bg_smrna.php?coord=chr3:32893344-32893370:pos&rnaID=piR-34005&dataSource=DASHR2_GEO_hg38
166	Sequence 1	UGUCACUUGCUCUUUGGUC	piRNA	GCCGUGUCUGCAGCUGAAAGUCACUUAGGA	30	53.33	0.125	0	0.233	0.233	0.3	0.233	Homo sapiens	https://dashr2.lisanwanglab.org/get_bg_smrna.php?coord=chr6:33877181-33877210:neg&rnaID=piR-35438&dataSource=DASHR2_GEO_hg38
167	Sequence 1	UGUCACUUGCUCUUUGGUC	piRNA	UCCAGGGUCUGUCACUUUGGAGAAUGAGCCC	31	54.84	0.059	-0.143	0.194	0.258	0.29	0.258	Homo sapiens	https://dashr2.lisanwanglab.org/get_bg_smrna.php?coord=chr6:33909550-33909580:pos&rnaID=piR-42813&dataSource=DASHR2_GEO_hg38
168	Sequence 1	UGUCACUUGCUCUUUGGUC	piRNA	UAGCCACCAAGUCUAGUCACUUGAAC	26	46.15	-0.333	0.143	0.308	0.308	0.154	0.231	Homo sapiens	https://dashr2.lisanwanglab.org/get_bg_smrna.php?coord=chr6:64330615-64330640:neg&rnaID=piR-39284&dataSource=DASHR2_GEO_hg38
169	Sequence 1	UGUCACUUGCUCUUUGGUC	piRNA	UGUCACUUUCCUGUCAGCCAUAGAAUCUGC	30	46.67	-0.286	-0.25	0.2	0.3	0.167	0.333	Homo sapiens	https://dashr2.lisanwanglab.org/get_bg_smrna.php?coord=chr6:166085106-166085135:neg&rnaID=piR-58058&dataSource=DASHR2_GEO_hg38
170	Sequence 1	UGUCACUUGCUCUUUGGUC	piRNA	UCGUCACUUGCUAUGUCCUCACUGGC	26	53.85	-0.286	-0.5	0.115	0.346	0.192	0.346	Homo sapiens	https://dashr2.lisanwanglab.org/get_bg_smrna.php?coord=chr7:128657536-128657561:pos&rnaID=piR-45199&dataSource=DASHR2_GEO_hg38
171	Sequence 1	UGUCACUUGCUCUUUGGUC	piRNA	UGGUCACUUUGAGGUCCCUGGAGGGAUGGGU	31	58.06	0.444	-0.385	0.129	0.161	0.419	0.29	Homo sapiens	https://dashr2.lisanwanglab.org/get_bg_smrna.php?coord=chr8:43512858-43512888:pos&rnaID=piR-57053&dataSource=DASHR2_GEO_hg38
172	Sequence 1	UGUCACUUGCUCUUUGGUC	piRNA	UGGGAUGUCAAAGCACGAGGGUCACUUG	28	53.57	0.333	0.077	0.25	0.179	0.357	0.214	Homo sapiens	https://dashr2.lisanwanglab.org/get_bg_smrna.php?coord=chr8:60375648-60375675:pos&rnaID=piR-56019&dataSource=DASHR2_GEO_hg38
173	Sequence 1	UGUCACUUGCUCUUUGGUC	piRNA	UAGGGUCGCAGGUGACAUGGUCACUU	26	53.85	0.286	-0.167	0.192	0.192	0.346	0.269	Homo sapiens	https://dashr2.lisanwanglab.org/get_bg_smrna.php?coord=chrX:26199697-26199722:neg&rnaID=piR-39764&dataSource=DASHR2_GEO_hg38
174	Sequence 2	ACACCCGUCACCACUGCUC	siRNA	UUCCGCACCCGUUCAGUCC	19	63.16	-0.5	-0.429	0.105	0.474	0.158	0.263	Homo Sapiens	
175	Sequence 2	ACACCCGUCACCACUGCUC	siRNA	UUCAUCUCCACCCGUUUGA	19	47.37	-0.556	-0.4	0.158	0.368	0.105	0.368	Homo Sapiens	
176	Sequence 2	ACACCCGUCACCACUGCUC	siRNA	AUUCAUAUCACCCGUGCCC	19	52.63	-0.6	-0.111	0.211	0.421	0.105	0.263	Homo Sapiens	
177	Sequence 2	ACACCCGUCACCACUGCUC	siRNA	UCACCCGUUUGCCUUCUCC	19	57.89	-0.636	-0.75	0.053	0.474	0.105	0.368	Homo Sapiens	
178	Sequence 2	ACACCCGUCACCACUGCUC	siRNA	UACUUCCACACACCCGUGA	19	52.63	-0.6	0.111	0.263	0.421	0.105	0.211	Homo Sapiens	
179	Sequence 2	ACACCCGUCACCACUGCUC	siRNA	AUCUUCGCCACCCGUCACC	19	63.16	-0.667	-0.143	0.158	0.526	0.105	0.211	Homo Sapiens	
180	Sequence 2	ACACCCGUCACCACUGCUC	siRNA	UACCUGGGCACCCGUAGUC	19	63.16	-0.167	-0.143	0.158	0.368	0.263	0.211	Homo Sapiens	
181	Sequence 2	ACACCCGUCACCACUGCUC	siRNA	UUCCACAACACCCGUAAUC	19	47.37	-0.778	0.2	0.316	0.421	0.053	0.211	Homo Sapiens	
182	Sequence 2	ACACCCGUCACCACUGCUC	siRNA	UCAGAUUCCACCCGUUAGC	19	52.63	-0.4	-0.111	0.211	0.368	0.158	0.263	Homo Sapiens	
183	Sequence 2	ACACCCGUCACCACUGCUC	siRNA	ACACCCGUUAGUUUGUCAA	19	42.11	-0.25	-0.091	0.263	0.263	0.158	0.316	Homo Sapiens	
184	Sequence 2	ACACCCGUCACCACUGCUC	siRNA	UUUCCGCCAACACCCGUGC	19	63.16	-0.5	-0.143	0.158	0.474	0.158	0.211	Homo Sapiens	
185	Sequence 2	ACACCCGUCACCACUGCUC	siRNA	AUAUCUUCACCCGUCGACG	19	52.63	-0.4	-0.111	0.211	0.368	0.158	0.263	Homo Sapiens	
186	Sequence 2	ACACCCGUCACCACUGCUC	siRNA	ACACCCGUGGCCUCUUCAC	19	63.16	-0.5	-0.143	0.158	0.474	0.158	0.211	Homo Sapiens	
187	Sequence 2	ACACCCGUCACCACUGCUC	siRNA	ACACCCGUCACCUUUGUUG	19	52.63	-0.4	-0.333	0.158	0.368	0.158	0.316	Homo Sapiens	
188	Sequence 2	ACACCCGUCACCACUGCUC	siRNA	UUCUUCAGCACCCGUAUUC	19	47.37	-0.556	-0.4	0.158	0.368	0.105	0.368	Homo Sapiens	
189	Sequence 2	ACACCCGUCACCACUGCUC	siRNA	AUAGCCAGCACCCGUGUUC	19	57.89	-0.273	0	0.211	0.368	0.211	0.211	Homo Sapiens	
190	Sequence 2	ACACCCGUCACCACUGCUC	siRNA	AUAAUCACCCGUCCAGUGC	19	52.63	-0.4	0.111	0.263	0.368	0.158	0.211	Homo Sapiens	
191	Sequence 2	ACACCCGUCACCACUGCUC	siRNA	UAAUCGGCCACCCGUUCCA	19	57.89	-0.455	0	0.211	0.421	0.158	0.211	Homo Sapiens	
192	Sequence 2	ACACCCGUCACCACUGCUC	siRNA	UUAGGCAGCACACCCGUGG	19	63.16	0	0.143	0.211	0.316	0.316	0.158	Homo Sapiens	
193	Sequence 2	ACACCCGUCACCACUGCUC	siRNA	UACGUACCACCCGUUCUUC	19	52.63	-0.6	-0.333	0.158	0.421	0.105	0.316	Homo Sapiens	
194	Sequence 2	ACACCCGUCACCACUGCUC	siRNA	UUGCCACCCGUUGACUUGC	19	57.89	-0.273	-0.5	0.105	0.368	0.211	0.316	Homo Sapiens	
195	Sequence 2	ACACCCGUCACCACUGCUC	siRNA	UCACCGGCCACCCGUUUAG	19	63.16	-0.333	-0.143	0.158	0.421	0.211	0.211	Homo Sapiens	
196	Sequence 2	ACACCCGUCACCACUGCUC	siRNA	AGACGCAGCACCCGUUACC	19	63.16	-0.333	0.429	0.263	0.421	0.211	0.105	Homo Sapiens	
197	Sequence 2	ACACCCGUCACCACUGCUC	siRNA	UCACCCGUUAACUCCAUUA	19	42.11	-0.75	-0.091	0.263	0.368	0.053	0.316	Homo Sapiens	
198	Sequence 2	ACACCCGUCACCACUGCUC	siRNA	UAGGUCAGCACCCGUAUUG	19	52.63	0	-0.111	0.211	0.263	0.263	0.263	Homo Sapiens	
199	Sequence 2	ACACCCGUCACCACUGCUC	siRNA	UUCCUCACCCGUGUUAUGC	19	52.63	-0.4	-0.556	0.105	0.368	0.158	0.368	Homo Sapiens	
200	Sequence 2	ACACCCGUCACCACUGCUC	siRNA	ACACCCGUAAGAUAAUCCG	19	47.37	-0.333	0.4	0.368	0.316	0.158	0.158	Homo Sapiens	
201	Sequence 2	ACACCCGUCACCACUGCUC	siRNA	UCUAGCACCCGUUCAUUAC	19	47.37	-0.556	-0.2	0.211	0.368	0.105	0.316	Homo Sapiens	
202	Sequence 2	ACACCCGUCACCACUGCUC	siRNA	UCACCCGUCACUCUUCCAU	19	52.63	-0.8	-0.333	0.158	0.474	0.053	0.316	Homo Sapiens	
203	Sequence 2	ACACCCGUCACCACUGCUC	miRNA	CACCCGUACAUAUGUUUCCGUGCU	24	50	-0.333	-0.333	0.167	0.333	0.167	0.333	Caenorhabditis elegans;Caenorhabditis briggsae;Caenorhabditis remanei;Caenorhabditis brenneri	https://mirbase.org/mature/MIMAT0000023;https://mirbase.org/mature/MIMAT0000476;https://mirbase.org/mature/MIMAT0011549;https://mirbase.org/mature/MIMAT0029686
204	Sequence 2	ACACCCGUCACCACUGCUC	miRNA	CACCCGUACAUUUGUUUCCGUGCU	24	50	-0.333	-0.5	0.125	0.333	0.167	0.375	Caenorhabditis elegans	https://mirbase.org/mature/MIMAT0000024
205	Sequence 2	ACACCCGUCACCACUGCUC	miRNA	CACCCGUAGAACCGACCUUGCG	22	63.64	-0.286	0.25	0.227	0.409	0.227	0.136	Homo sapiens;Mus musculus;Rattus norvegicus;Bos taurus;Sus scrofa;Macaca mulatta;Canis familiaris;Pan troglodytes;Equus caballus;Capra hircus;Tupaia chinensis;Pteropus alecto;Cavia porcellus;Otolemur garnettii	https://mirbase.org/mature/MIMAT0000689;https://mirbase.org/mature/MIMAT0000132;https://mirbase.org/mature/MIMAT0000821;https://mirbase.org/mature/MIMAT0004345;https://mirbase.org/mature/MIMAT0006018;https://mirbase.org/mature/MIMAT0006179;https://mirbase.org/mature/MIMAT0006607;https://mirbase.org/mature/MIMAT0008351;https://mirbase.org/mature/MIMAT0013018;https://mirbase.org/mature/MIMAT0036314;https://mirbase.org/mature/MIMAT0036582;https://mirbase.org/mature/MIMAT0039834;https://mirbase.org/mature/MIMAT0046943;https://mirbase.org/mature/MIMAT0049653
206	Sequence 2	ACACCCGUCACCACUGCUC	miRNA	UCUCACACAGAAAUCGCACCCGU	23	52.17	-0.5	0.273	0.304	0.391	0.13	0.174	Homo sapiens;Rattus norvegicus;Mus musculus;Macaca mulatta;Canis familiaris;Pan troglodytes;Equus caballus;Pongo pygmaeus;Tupaia chinensis;Pteropus alecto	https://mirbase.org/mature/MIMAT0000753;https://mirbase.org/mature/MIMAT0000589;https://mirbase.org/mature/MIMAT0000590;https://mirbase.org/mature/MIMAT0006283;https://mirbase.org/mature/MIMAT0006709;https://mirbase.org/mature/MIMAT0008110;https://mirbase.org/mature/MIMAT0013137;https://mirbase.org/mature/MIMAT0015845;https://mirbase.org/mature/MIMAT0036487;https://mirbase.org/mature/MIMAT0040086
207	Sequence 2	ACACCCGUCACCACUGCUC	miRNA	UAGGUCACCCGUUUUACUAUC	21	42.86	-0.333	-0.333	0.19	0.286	0.143	0.381	Mus musculus	https://mirbase.org/mature/MIMAT0005851
208	Sequence 2	ACACCCGUCACCACUGCUC	miRNA	UAGGUCACCCGUUUGACUAUC	21	47.62	-0.2	-0.273	0.19	0.286	0.19	0.333	Bos taurus;Equus caballus;Callithrix jacchus	https://mirbase.org/mature/MIMAT0009965;https://mirbase.org/mature/MIMAT0013124;https://mirbase.org/mature/MIMAT0039620
209	Sequence 2	ACACCCGUCACCACUGCUC	miRNA	AAUUGCACCCGUCCCGGCCUGA	22	63.64	-0.286	0	0.182	0.409	0.227	0.182	Apis mellifera	https://mirbase.org/mature/MIMAT0010117
210	Sequence 2	ACACCCGUCACCACUGCUC	miRNA	CACCCGUAUUGCUUCCUCUGAG	22	54.55	-0.333	-0.4	0.136	0.364	0.182	0.318	Caenorhabditis briggsae	https://mirbase.org/mature/MIMAT0011476
211	Sequence 2	ACACCCGUCACCACUGCUC	miRNA	CACCCGUAGUCCGUUAUCGUCU	22	54.55	-0.333	-0.4	0.136	0.364	0.182	0.318	Pristionchus pacificus	https://mirbase.org/mature/MIMAT0011734
212	Sequence 2	ACACCCGUCACCACUGCUC	miRNA	UCUCACACAGAAAUCGCACCCGUCA	25	52	-0.538	0.333	0.32	0.4	0.12	0.16	Sus scrofa;Eptesicus fuscus;Cavia porcellus;Dasypus novemcinctus	https://mirbase.org/mature/MIMAT0013944;https://mirbase.org/mature/MIMAT0034966;https://mirbase.org/mature/MIMAT0047181;https://mirbase.org/mature/MIMAT0047826
213	Sequence 2	ACACCCGUCACCACUGCUC	miRNA	UAGGUCACCCGUUUUACUAUCC	22	45.45	-0.4	-0.333	0.182	0.318	0.136	0.364	Rattus norvegicus	https://mirbase.org/mature/MIMAT0017859
214	Sequence 2	ACACCCGUCACCACUGCUC	miRNA	UAGGUCACCCGUUUGACUAUCC	22	50	-0.273	-0.273	0.182	0.318	0.182	0.318	Ovis aries	https://mirbase.org/mature/MIMAT0019270
215	Sequence 2	ACACCCGUCACCACUGCUC	miRNA	UCUCACACAGAAAUCGCACCCGUC	24	54.17	-0.538	0.273	0.292	0.417	0.125	0.167	Cricetulus griseus;Callithrix jacchus;Microcebus murinus;Otolemur garnettii	https://mirbase.org/mature/MIMAT0023923;https://mirbase.org/mature/MIMAT0039473;https://mirbase.org/mature/MIMAT0049166;https://mirbase.org/mature/MIMAT0049680
216	Sequence 2	ACACCCGUCACCACUGCUC	miRNA	ACACCCGUACGAGUGACAGAAUA	23	47.83	-0.091	0.5	0.391	0.261	0.217	0.13	Tetranychus urticae	https://mirbase.org/mature/MIMAT0026469
217	Sequence 2	ACACCCGUCACCACUGCUC	miRNA	UCACCCGUGUCCUUUCUCUGCCUAG	25	56	-0.429	-0.636	0.08	0.4	0.16	0.36	Equus caballus	https://mirbase.org/mature/MIMAT0034640
218	Sequence 2	ACACCCGUCACCACUGCUC	miRNA	CACCCGUAGAACCGACCUUGCGGGG	25	68	-0.059	0.25	0.2	0.36	0.32	0.12	Eptesicus fuscus	https://mirbase.org/mature/MIMAT0035185
219	Sequence 2	ACACCCGUCACCACUGCUC	miRNA	CGCGCACCCGUUCAUCUUCGC	21	66.67	-0.429	-0.429	0.095	0.476	0.19	0.238	Fragaria vesca	https://mirbase.org/mature/MIMAT0044596
220	Sequence 2	ACACCCGUCACCACUGCUC	miRNA	UGCACCACCCGUGGUCACUCCU	22	63.64	-0.429	-0.25	0.136	0.455	0.182	0.227	Bos taurus	https://mirbase.org/mature/MIMAT0046367
221	Sequence 2	ACACCCGUCACCACUGCUC	miRNA	UGUAGGUCACCCGUUUCACUAUC	23	47.83	-0.273	-0.333	0.174	0.304	0.174	0.348	Cavia porcellus	https://mirbase.org/mature/MIMAT0047402
222	Sequence 2	ACACCCGUCACCACUGCUC	piRNA	GCUAAAAGAGCACACCCGUCUAUGUAGCAAA	31	45.16	-0.143	0.412	0.387	0.258	0.194	0.161	Homo sapiens	https://dashr2.lisanwanglab.org/get_bg_smrna.php?coord=chr11:10508897-10508927:neg&rnaID=piR-35548&dataSource=DASHR2_GEO_hg38;https://dashr2.lisanwanglab.org/get_bg_smrna.php?coord=chr3:160947661-160947691:pos&rnaID=piR-35548&dataSource=DASHR2_GEO_hg38;https://dashr2.lisanwanglab.org/get_bg_smrna.php?coord=chr3:96617944-96617974:neg&rnaID=piR-35548&dataSource=DASHR2_GEO_hg38;https://dashr2.lisanwanglab.org/get_bg_smrna.php?coord=chrM:1932-1962:pos&rnaID=piR-35548&dataSource=DASHR2_GEO_hg38
223	Sequence 2	ACACCCGUCACCACUGCUC	piRNA	UCCUGUAGAAGAUCCCACCCGUACCAGCC	29	58.62	-0.412	0.167	0.241	0.414	0.172	0.172	Homo sapiens	https://dashr2.lisanwanglab.org/get_bg_smrna.php?coord=chr15:20183783-20183811:pos&rnaID=piR-44652&dataSource=DASHR2_GEO_hg38;https://dashr2.lisanwanglab.org/get_bg_smrna.php?coord=chr15:21190333-21190361:pos&rnaID=piR-44652&dataSource=DASHR2_GEO_hg38
224	Sequence 2	ACACCCGUCACCACUGCUC	piRNA	UCCACCCGUACAGCGACUUCAGGUUC	26	57.69	-0.333	-0.091	0.192	0.385	0.192	0.231	Homo sapiens	https://dashr2.lisanwanglab.org/get_bg_smrna.php?coord=chr15:30223517-30223542:pos&rnaID=piR-42482&dataSource=DASHR2_GEO_hg38;https://dashr2.lisanwanglab.org/get_bg_smrna.php?coord=chr15:32315147-32315172:neg&rnaID=piR-42482&dataSource=DASHR2_GEO_hg38
225	Sequence 2	ACACCCGUCACCACUGCUC	piRNA	UCUGAACAUCUAGAGAGACCCACCCGUCAC	30	53.33	-0.375	0.286	0.3	0.367	0.167	0.167	Homo sapiens	https://dashr2.lisanwanglab.org/get_bg_smrna.php?coord=chr15:92602068-92602097:pos&rnaID=piR-45789&dataSource=DASHR2_GEO_hg38
226	Sequence 2	ACACCCGUCACCACUGCUC	piRNA	UACUUACUCUGAGAUCACCCGUUUGC	26	46.15	-0.333	-0.286	0.192	0.308	0.154	0.346	Homo sapiens	https://dashr2.lisanwanglab.org/get_bg_smrna.php?coord=chr2:131408015-131408040:pos&rnaID=piR-38708&dataSource=DASHR2_GEO_hg38
227	Sequence 2	ACACCCGUCACCACUGCUC	piRNA	UGAGAUUUUGGUGCACCCGUCAUGUGAGCA	30	50	0.2	-0.2	0.2	0.2	0.3	0.3	Homo sapiens	https://dashr2.lisanwanglab.org/get_bg_smrna.php?coord=chr9:90560383-90560412:neg&rnaID=piR-48620&dataSource=DASHR2_GEO_hg38
228	Sequence 2	ACACCCGUCACCACUGCUC	piRNA	GGCGUGUAAAGCAGGUGGUCACCCGUC	27	62.96	0.176	0	0.185	0.259	0.37	0.185	Homo sapiens	https://dashr2.lisanwanglab.org/get_bg_smrna.php?coord=chr9:136120493-136120519:pos&rnaID=piR-36066&dataSource=DASHR2_GEO_hg38
229	Sequence 3	UUAGGCUUCAGGUUCGUAG	siRNA	CUAAUAGGCUUACAUUUAC	19	31.58	-0.333	-0.077	0.316	0.211	0.105	0.368	Homo Sapiens	
230	Sequence 3	UUAGGCUUCAGGUUCGUAG	siRNA	UAAUAUAGGCUUUCCUCGG	19	42.11	0	-0.273	0.211	0.211	0.211	0.368	Homo Sapiens	
231	Sequence 3	UUAGGCUUCAGGUUCGUAG	siRNA	UGUUGUUUAGGCUUAGCGU	19	42.11	0.5	-0.636	0.105	0.105	0.316	0.474	Homo Sapiens	
232	Sequence 3	UUAGGCUUCAGGUUCGUAG	siRNA	UACUCAUAGGCUUCCGUCC	19	52.63	-0.4	-0.333	0.158	0.368	0.158	0.316	Homo Sapiens	
233	Sequence 3	UUAGGCUUCAGGUUCGUAG	siRNA	UUUAGGAUUAGGCUUUAAC	19	31.58	0.333	-0.231	0.263	0.105	0.211	0.421	Homo Sapiens	
234	Sequence 3	UUAGGCUUCAGGUUCGUAG	siRNA	AUAGGCUUCAUACCUGCUC	19	47.37	-0.333	-0.2	0.211	0.316	0.158	0.316	Homo Sapiens	
235	Sequence 3	UUAGGCUUCAGGUUCGUAG	siRNA	UUCACAAUAGGCUUGGAGC	19	47.37	0.111	0	0.263	0.211	0.263	0.263	Homo Sapiens	
236	Sequence 3	UUAGGCUUCAGGUUCGUAG	siRNA	UCUGUUUAUAGGCUUUAGC	19	36.84	0.143	-0.5	0.158	0.158	0.211	0.474	Homo Sapiens	
237	Sequence 3	UUAGGCUUCAGGUUCGUAG	siRNA	AGAUCCGCUAGGCUUCCAG	19	57.89	-0.091	0	0.211	0.316	0.263	0.211	Homo Sapiens	
238	Sequence 3	UUAGGCUUCAGGUUCGUAG	siRNA	UUAGGCUUGAUAAGUGUUC	19	36.84	0.429	-0.333	0.211	0.105	0.263	0.421	Homo Sapiens	
239	Sequence 3	UUAGGCUUCAGGUUCGUAG	siRNA	ACCUAGGCUUGAUGAUUUC	19	42.11	0	-0.273	0.211	0.211	0.211	0.368	Homo Sapiens	
240	Sequence 3	UUAGGCUUCAGGUUCGUAG	siRNA	AUAGGCUUUGAUAACCUCC	19	42.11	-0.25	-0.091	0.263	0.263	0.158	0.316	Homo Sapiens	
241	Sequence 3	UUAGGCUUCAGGUUCGUAG	siRNA	UGUAGUUAGGCUUCCGAUU	19	42.11	0.25	-0.455	0.158	0.158	0.263	0.421	Homo Sapiens	
242	Sequence 3	UUAGGCUUCAGGUUCGUAG	siRNA	UAGGCUUAGAGGUCCCAGC	19	57.89	0.091	0	0.211	0.263	0.316	0.211	Homo Sapiens	
243	Sequence 3	UUAGGCUUCAGGUUCGUAG	siRNA	UAACUUUGUAGGCUUUAGA	19	31.58	0.333	-0.231	0.263	0.105	0.211	0.421	Homo Sapiens	
244	Sequence 3	UUAGGCUUCAGGUUCGUAG	siRNA	AAUCCUAGGCUUCUUUACC	19	42.11	-0.5	-0.273	0.211	0.316	0.105	0.368	Homo Sapiens	
245	Sequence 3	UUAGGCUUCAGGUUCGUAG	siRNA	UCUUCGUAGGCUUCAUAUC	19	42.11	-0.25	-0.455	0.158	0.263	0.158	0.421	Homo Sapiens	
246	Sequence 3	UUAGGCUUCAGGUUCGUAG	siRNA	UAGGCUUGCCCUGAAUUUC	19	47.37	-0.111	-0.4	0.158	0.263	0.211	0.368	Homo Sapiens	
247	Sequence 3	UUAGGCUUCAGGUUCGUAG	siRNA	UAGUCGUAGGCUUGGAUGC	19	52.63	0.4	-0.333	0.158	0.158	0.368	0.316	Homo Sapiens	
248	Sequence 3	UUAGGCUUCAGGUUCGUAG	siRNA	AUAGGCUUUGUGUCAGCAC	19	47.37	0.111	-0.2	0.211	0.211	0.263	0.316	Homo Sapiens	
249	Sequence 3	UUAGGCUUCAGGUUCGUAG	siRNA	UUAGGCUUAACUUCAGGUA	19	36.84	0.143	-0.167	0.263	0.158	0.211	0.368	Homo Sapiens	
250	Sequence 3	UUAGGCUUCAGGUUCGUAG	siRNA	UAGGCUUAAGGAACUUUAC	19	36.84	0.143	0	0.316	0.158	0.211	0.316	Homo Sapiens	
251	Sequence 3	UUAGGCUUCAGGUUCGUAG	siRNA	GUAGGCUUCAGGCAGGUAC	19	57.89	0.273	0	0.211	0.211	0.368	0.211	Homo Sapiens	
252	Sequence 3	UUAGGCUUCAGGUUCGUAG	siRNA	GUUCAGUCUAGGCUUAUCC	19	47.37	-0.111	-0.4	0.158	0.263	0.211	0.368	Homo Sapiens	
253	Sequence 3	UUAGGCUUCAGGUUCGUAG	siRNA	UUAGGCUUCAGUGUAUUGC	19	42.11	0.25	-0.455	0.158	0.158	0.263	0.421	Homo Sapiens	
254	Sequence 3	UUAGGCUUCAGGUUCGUAG	siRNA	AUAGGCUUGAGAGAUAUCC	19	42.11	0.25	0.091	0.316	0.158	0.263	0.263	Homo Sapiens	
255	Sequence 3	UUAGGCUUCAGGUUCGUAG	siRNA	AUACUUGUUAGGCUUCAUG	19	36.84	0.143	-0.333	0.211	0.158	0.211	0.421	Homo Sapiens	
256	Sequence 3	UUAGGCUUCAGGUUCGUAG	siRNA	UUAGGCUUCUCACCUGUAC	19	47.37	-0.333	-0.4	0.158	0.316	0.158	0.368	Homo Sapiens	
257	Sequence 3	UUAGGCUUCAGGUUCGUAG	siRNA	AAUCUUACAAUCUAGGCUU	19	31.58	-0.333	-0.077	0.316	0.211	0.105	0.368	Homo Sapiens	
258	Sequence 3	UUAGGCUUCAGGUUCGUAG	siRNA	UGAUUCUAGGCUUUACUCG	19	42.11	0	-0.455	0.158	0.211	0.211	0.421	Homo Sapiens	
259	Sequence 3	UUAGGCUUCAGGUUCGUAG	siRNA	AUAGGCUUGCACUCCAUUG	19	47.37	-0.111	-0.2	0.211	0.263	0.211	0.316	Homo Sapiens	
260	Sequence 3	UUAGGCUUCAGGUUCGUAG	siRNA	AACAUCGGUAGGCUUAAUU	19	36.84	0.143	0	0.316	0.158	0.211	0.316	Homo Sapiens	
261	Sequence 3	UUAGGCUUCAGGUUCGUAG	siRNA	AGAGUUUAGGCUUAUGAUC	19	36.84	0.429	-0.167	0.263	0.105	0.263	0.368	Homo Sapiens	
262	Sequence 3	UUAGGCUUCAGGUUCGUAG	siRNA	AGUUACUAGGCUUCGAUCC	19	47.37	-0.111	-0.2	0.211	0.263	0.211	0.316	Homo Sapiens	
263	Sequence 3	UUAGGCUUCAGGUUCGUAG	siRNA	UCGAACAUAGGCUUUGGCG	19	52.63	0.2	-0.111	0.211	0.211	0.316	0.263	Homo Sapiens	
264	Sequence 3	UUAGGCUUCAGGUUCGUAG	siRNA	CUUGUAGUAGGCUUUGGCG	19	52.63	0.4	-0.556	0.105	0.158	0.368	0.368	Homo Sapiens	
265	Sequence 3	UUAGGCUUCAGGUUCGUAG	siRNA	UUAGGCUUCUGAUUUGCUG	19	42.11	0.25	-0.636	0.105	0.158	0.263	0.474	Homo Sapiens	
266	Sequence 3	UUAGGCUUCAGGUUCGUAG	siRNA	UGUUACGUAGGCUUGGAUC	19	47.37	0.333	-0.4	0.158	0.158	0.316	0.368	Homo Sapiens	
267	Sequence 3	UUAGGCUUCAGGUUCGUAG	siRNA	GUACUAGUUAGGCUUGUGG	19	47.37	0.556	-0.4	0.158	0.105	0.368	0.368	Homo Sapiens	
268	Sequence 3	UUAGGCUUCAGGUUCGUAG	siRNA	AAUACGGUAGGCUUUGCCA	19	47.37	0.111	0	0.263	0.211	0.263	0.263	Homo Sapiens	
269	Sequence 3	UUAGGCUUCAGGUUCGUAG	siRNA	ACGUUGUGUAGGCUUUCCC	19	52.63	0	-0.556	0.105	0.263	0.263	0.368	Homo Sapiens	
270	Sequence 3	UUAGGCUUCAGGUUCGUAG	siRNA	UUAGGCUUCUCUUUCGUCA	19	42.11	-0.25	-0.636	0.105	0.263	0.158	0.474	Homo Sapiens	
271	Sequence 3	UUAGGCUUCAGGUUCGUAG	siRNA	AAACUCAUUAGGCUUGAAC	19	36.84	-0.143	0.167	0.368	0.211	0.158	0.263	Homo Sapiens	
272	Sequence 3	UUAGGCUUCAGGUUCGUAG	siRNA	UCGUCUAGUAGGCUUGUCA	19	47.37	0.111	-0.4	0.158	0.211	0.263	0.368	Homo Sapiens	
273	Sequence 3	UUAGGCUUCAGGUUCGUAG	siRNA	UACUAAUAGGCUUAAACGA	19	31.58	0	0.231	0.421	0.158	0.158	0.263	Homo Sapiens	
274	Sequence 3	UUAGGCUUCAGGUUCGUAG	siRNA	CAUACGAUAGGCUUAAUCC	19	42.11	-0.25	0.091	0.316	0.263	0.158	0.263	Homo Sapiens	
275	Sequence 3	UUAGGCUUCAGGUUCGUAG	siRNA	AUAGCAUUAGGCUUUAUCC	19	36.84	-0.143	-0.167	0.263	0.211	0.158	0.368	Homo Sapiens	
276	Sequence 3	UUAGGCUUCAGGUUCGUAG	siRNA	UAGGUCGUUAGGCUUGCGC	19	57.89	0.273	-0.5	0.105	0.211	0.368	0.316	Homo Sapiens	
277	Sequence 3	UUAGGCUUCAGGUUCGUAG	siRNA	AUAUCGGUAGGCUUCUUUC	19	42.11	0	-0.455	0.158	0.211	0.211	0.421	Homo Sapiens	
278	Sequence 3	UUAGGCUUCAGGUUCGUAG	siRNA	UUUUGCGCUUCUUAGGCUU	19	42.11	0	-0.818	0.053	0.211	0.211	0.526	Homo Sapiens	

	Sequence ID	siRNA Sequence	Type	Sequence	Length	GC %	GC skew	AT/AU Skew	MNC A	MNC C	MNC G	MNC U	Species	Link
279	Sequence 3	UUAGGCUUCAGGUUCGUAG	siRNA	AUGAUCAUAGGCUUUCACC	19	42.11	-0.25	-0.091	0.263	0.263	0.158	0.316	Homo Sapiens	
280	Sequence 3	UUAGGCUUCAGGUUCGUAG	siRNA	ACGUGUUAGGCUUUAGCUC	19	47.37	0.111	-0.4	0.158	0.211	0.263	0.368	Homo Sapiens	
281	Sequence 3	UUAGGCUUCAGGUUCGUAG	siRNA	UAGGCUUAGUCCGUCUUCC	19	52.63	-0.2	-0.556	0.105	0.316	0.211	0.368	Homo Sapiens	
282	Sequence 3	UUAGGCUUCAGGUUCGUAG	siRNA	AACAUCGUAGGCUUGGCCC	19	57.89	-0.091	0	0.211	0.316	0.263	0.211	Homo Sapiens	
283	Sequence 3	UUAGGCUUCAGGUUCGUAG	siRNA	GUAGGCUUGAACCUUGUCA	19	47.37	0.111	-0.2	0.211	0.211	0.263	0.316	Homo Sapiens	
284	Sequence 3	UUAGGCUUCAGGUUCGUAG	siRNA	UAUAGCGUAGGCUUUUCUG	19	42.11	0.25	-0.455	0.158	0.158	0.263	0.421	Homo Sapiens	
285	Sequence 3	UUAGGCUUCAGGUUCGUAG	siRNA	UAUGUAGGCUUUUAUUAGG	19	31.58	0.667	-0.385	0.211	0.053	0.263	0.474	Homo Sapiens	
286	Sequence 3	UUAGGCUUCAGGUUCGUAG	siRNA	UGAUCGUAGGCUUCACAUG	19	47.37	0.111	-0.2	0.211	0.211	0.263	0.316	Homo Sapiens	
287	Sequence 3	UUAGGCUUCAGGUUCGUAG	siRNA	UAGGCUUGAAUAGUUUCUG	19	36.84	0.429	-0.333	0.211	0.105	0.263	0.421	Homo Sapiens	
288	Sequence 3	UUAGGCUUCAGGUUCGUAG	siRNA	UAGGACGAUAGGCUUGCCC	19	57.89	0.091	0	0.211	0.263	0.316	0.211	Homo Sapiens	
289	Sequence 3	UUAGGCUUCAGGUUCGUAG	siRNA	UAUGUAGGCUUCAAGGUUC	19	42.11	0.25	-0.273	0.211	0.158	0.263	0.368	Homo Sapiens	
290	Sequence 3	UUAGGCUUCAGGUUCGUAG	siRNA	ACACUCGUAGGCUUCAUAC	19	47.37	-0.333	0	0.263	0.316	0.158	0.263	Homo Sapiens	
291	Sequence 3	UUAGGCUUCAGGUUCGUAG	siRNA	UAGGCUUGCUCAUAUUUAA	19	31.58	0	-0.231	0.263	0.158	0.158	0.421	Homo Sapiens	
292	Sequence 3	UUAGGCUUCAGGUUCGUAG	siRNA	UUGUCAUAGGCUUCCCAUG	19	47.37	-0.111	-0.4	0.158	0.263	0.211	0.368	Homo Sapiens	
293	Sequence 3	UUAGGCUUCAGGUUCGUAG	siRNA	UGUACCUAUAGGCUUCCCG	19	52.63	-0.2	-0.333	0.158	0.316	0.211	0.316	Homo Sapiens	
294	Sequence 3	UUAGGCUUCAGGUUCGUAG	siRNA	AGGCGCGUAGGCUUUGCAG	19	63.16	0.333	-0.143	0.158	0.211	0.421	0.211	Homo Sapiens	
295	Sequence 3	UUAGGCUUCAGGUUCGUAG	siRNA	AUAGGCUUUCACUGAGACC	19	47.37	-0.111	0	0.263	0.263	0.211	0.263	Homo Sapiens	
296	Sequence 3	UUAGGCUUCAGGUUCGUAG	siRNA	GUAGAUGUUAGGCUUGUGC	19	47.37	0.556	-0.4	0.158	0.105	0.368	0.368	Homo Sapiens	
297	Sequence 3	UUAGGCUUCAGGUUCGUAG	siRNA	UUAGGCUUCAGGUCCGUGA	19	52.63	0.2	-0.333	0.158	0.211	0.316	0.316	Homo Sapiens	
298	Sequence 3	UUAGGCUUCAGGUUCGUAG	siRNA	UAAUAGGCUUUGGUGCAGC	19	47.37	0.333	-0.2	0.211	0.158	0.316	0.316	Homo Sapiens	
299	Sequence 3	UUAGGCUUCAGGUUCGUAG	siRNA	GACCUCAUAGGCUUCCUUG	19	52.63	-0.2	-0.333	0.158	0.316	0.211	0.316	Homo Sapiens	
300	Sequence 3	UUAGGCUUCAGGUUCGUAG	siRNA	UUAGGCUUGACAACCUUGG	19	47.37	0.111	-0.2	0.211	0.211	0.263	0.316	Homo Sapiens	
301	Sequence 3	UUAGGCUUCAGGUUCGUAG	siRNA	UGACCGUAGGGCUAGGCUU	19	57.89	0.273	-0.25	0.158	0.211	0.368	0.263	Homo Sapiens	
302	Sequence 3	UUAGGCUUCAGGUUCGUAG	siRNA	UAGGCUUAUGCCCCUUAGG	19	52.63	0	-0.333	0.158	0.263	0.263	0.316	Homo Sapiens	
303	Sequence 3	UUAGGCUUCAGGUUCGUAG	siRNA	CAAGACAUAGGCUUUCAAC	19	42.11	-0.25	0.273	0.368	0.263	0.158	0.211	Homo Sapiens	
304	Sequence 3	UUAGGCUUCAGGUUCGUAG	siRNA	UAUCUAUAGGCUUUGAGUC	19	36.84	0.143	-0.333	0.211	0.158	0.211	0.421	Homo Sapiens	
305	Sequence 3	UUAGGCUUCAGGUUCGUAG	siRNA	GUAGGCUUCCUCAAAAUGC	19	47.37	-0.111	0	0.263	0.263	0.211	0.263	Homo Sapiens	
306	Sequence 3	UUAGGCUUCAGGUUCGUAG	siRNA	AUUCGAAUCCCAUAGGCUU	19	42.11	-0.25	-0.091	0.263	0.263	0.158	0.316	Homo Sapiens	
307	Sequence 3	UUAGGCUUCAGGUUCGUAG	siRNA	GGGUAGGCUUCCUCAAAAU	19	47.37	0.111	0	0.263	0.211	0.263	0.263	Homo Sapiens	
308	Sequence 3	UUAGGCUUCAGGUUCGUAG	siRNA	CUUCCAGUAGGCUUCCAAG	19	52.63	-0.2	-0.111	0.211	0.316	0.211	0.263	Homo Sapiens	
309	Sequence 3	UUAGGCUUCAGGUUCGUAG	miRNA	UAGCCUCUACUAGGCUUUGUCUGU	24	45.83	-0.091	-0.538	0.125	0.25	0.208	0.417	Drosophila melanogaster	https://mirbase.org/mature/MIMAT0000346
310	Sequence 3	UUAGGCUUCAGGUUCGUAG	miRNA	AAUCUAGCCUCUACUAGGCUUUGUCUGU	28	42.86	-0.167	-0.375	0.179	0.25	0.179	0.393	Drosophila pseudoobscura;Drosophila erecta;Drosophila grimshawi;Drosophila mojavensis;Drosophila persimilis;Drosophila sechellia;Drosophila simulans;Drosophila willistoni;Drosophila yakuba	https://mirbase.org/mature/MIMAT0001246;https://mirbase.org/mature/MIMAT0008509;https://mirbase.org/mature/MIMAT0008614;https://mirbase.org/mature/MIMAT0008645;https://mirbase.org/mature/MIMAT0008716;https://mirbase.org/mature/MIMAT0008792;https://mirbase.org/mature/MIMAT0008883;https://mirbase.org/mature/MIMAT0009026;https://mirbase.org/mature/MIMAT0009071
311	Sequence 3	UUAGGCUUCAGGUUCGUAG	miRNA	GAUUUAGCCUCUCCUAGGCUUUGUCUGU	28	46.43	-0.077	-0.6	0.107	0.25	0.214	0.429	Apis mellifera;Nasonia vitripennis;Nasonia longicornis	https://mirbase.org/mature/MIMAT0001484;https://mirbase.org/mature/MIMAT0015693;https://mirbase.org/mature/MIMAT0018418
312	Sequence 3	UUAGGCUUCAGGUUCGUAG	miRNA	AAUCUAGCCUCUUCUAGGCUUUGUCUGU	28	42.86	-0.167	-0.5	0.143	0.25	0.179	0.429	Anopheles gambiae	https://mirbase.org/mature/MIMAT0001514
313	Sequence 3	UUAGGCUUCAGGUUCGUAG	miRNA	UUGUACAUGGUAGGCUUUCAUU	22	36.36	0.25	-0.429	0.182	0.136	0.227	0.455	Homo sapiens;Rattus norvegicus;Macaca mulatta;Ovis aries;Sus scrofa;Capra hircus;Callithrix jacchus;Pteropus alecto;Pan paniscus	https://mirbase.org/mature/MIMAT0002813;https://mirbase.org/mature/MIMAT0017217;https://mirbase.org/mature/MIMAT0026884;https://mirbase.org/mature/MIMAT0019237;https://mirbase.org/mature/MIMAT0025377;https://mirbase.org/mature/MIMAT0036253;https://mirbase.org/mature/MIMAT0039530;https://mirbase.org/mature/MIMAT0039943;https://mirbase.org/mature/MIMAT0049052
314	Sequence 3	UUAGGCUUCAGGUUCGUAG	miRNA	UAGCCUCUCCUAGGCUUUGUCU	22	50	-0.273	-0.636	0.091	0.318	0.182	0.409	Tribolium castaneum;Triops cancriformis;Dinoponera quadriceps;Polistes canadensis	https://mirbase.org/mature/MIMAT0008386;https://mirbase.org/mature/MIMAT0041399;https://mirbase.org/mature/MIMAT0045725;https://mirbase.org/mature/MIMAT0045895
315	Sequence 3	UUAGGCUUCAGGUUCGUAG	miRNA	GAUCUAGCCUCUACUAGGCUUUGUCUGU	28	46.43	-0.077	-0.467	0.143	0.25	0.214	0.393	Drosophila ananassae	https://mirbase.org/mature/MIMAT0008445
316	Sequence 3	UUAGGCUUCAGGUUCGUAG	miRNA	CUCUACUAGGCUUUGUCU	18	44.44	-0.25	-0.6	0.111	0.278	0.167	0.444	Drosophila virilis	https://mirbase.org/mature/MIMAT0008932
317	Sequence 3	UUAGGCUUCAGGUUCGUAG	miRNA	UGUGUCACUGGGGAUAGGCUUUG	23	52.17	0.5	-0.455	0.13	0.13	0.391	0.348	Mus musculus	https://mirbase.org/mature/MIMAT0009444
318	Sequence 3	UUAGGCUUCAGGUUCGUAG	miRNA	CAUACAUUGCCUCCUAGGCUUG	22	50	-0.273	-0.273	0.182	0.318	0.182	0.318	Oryza sativa	https://mirbase.org/mature/MIMAT0010047
319	Sequence 3	UUAGGCUUCAGGUUCGUAG	miRNA	UCAGGAUUCUAGGCUUAGAGGU	22	45.45	0.4	-0.167	0.227	0.136	0.318	0.318	Bos taurus	https://mirbase.org/mature/MIMAT0012004
320	Sequence 3	UUAGGCUUCAGGUUCGUAG	miRNA	GUGGAUUAGGCUUUGGCUU	19	47.37	0.556	-0.6	0.105	0.105	0.368	0.421	Equus caballus	https://mirbase.org/mature/MIMAT0012924
321	Sequence 3	UUAGGCUUCAGGUUCGUAG	miRNA	UACACUGUAGAAUAGGCUUGU	21	38.1	0.25	-0.077	0.286	0.143	0.238	0.333	Capitella teleta	https://mirbase.org/mature/MIMAT0013534
322	Sequence 3	UUAGGCUUCAGGUUCGUAG	miRNA	AAGAUGUUCUCAAUGUAGGCUU	22	36.36	0.25	-0.143	0.273	0.136	0.227	0.364	Capitella teleta	https://mirbase.org/mature/MIMAT0013536
323	Sequence 3	UUAGGCUUCAGGUUCGUAG	miRNA	AAUCUAGCCUCUCCUAGGCUUUGUCUG	27	48.15	-0.231	-0.429	0.148	0.296	0.185	0.37	Aedes aegypti	https://mirbase.org/mature/MIMAT0014311
324	Sequence 3	UUAGGCUUCAGGUUCGUAG	miRNA	UUGUACAUGGUAGGCUUUC	19	42.11	0.25	-0.455	0.158	0.158	0.263	0.421	Mus musculus	https://mirbase.org/mature/MIMAT0017276
325	Sequence 3	UUAGGCUUCAGGUUCGUAG	miRNA	GCUUCGUCUGAGGCUUAGGCUUAGGC	26	57.69	0.2	-0.455	0.115	0.231	0.346	0.308	Haemonchus contortus	https://mirbase.org/mature/MIMAT0023479
326	Sequence 3	UUAGGCUUCAGGUUCGUAG	miRNA	UUGUACAUGGUAGGCUUUCAU	21	38.1	0.25	-0.385	0.19	0.143	0.238	0.429	Gorilla gorilla	https://mirbase.org/mature/MIMAT0024249
327	Sequence 3	UUAGGCUUCAGGUUCGUAG	miRNA	UAAGUAGAUACUUCUAGGCUUU	22	31.82	0.143	-0.2	0.273	0.136	0.182	0.409	Manduca sexta	https://mirbase.org/mature/MIMAT0024500
328	Sequence 3	UUAGGCUUCAGGUUCGUAG	miRNA	UAGGCUUUAGGAGAGCUGA	19	47.37	0.556	0	0.263	0.105	0.368	0.263	Equus caballus	https://mirbase.org/mature/MIMAT0034471
329	Sequence 3	UUAGGCUUCAGGUUCGUAG	miRNA	UUGUACAUGGUAGGCUUUCAUUCAU	25	36	0.111	-0.375	0.2	0.16	0.2	0.44	Eptesicus fuscus	https://mirbase.org/mature/MIMAT0034929
330	Sequence 3	UUAGGCUUCAGGUUCGUAG	miRNA	UGGUAGACUAUGGAACGUAGGCUUU	25	44	0.455	-0.143	0.24	0.12	0.32	0.32	Eptesicus fuscus	https://mirbase.org/mature/MIMAT0035032
331	Sequence 3	UUAGGCUUCAGGUUCGUAG	miRNA	UGAGGGCUAGGCUUUUAUGUAA	22	40.91	0.556	-0.231	0.227	0.091	0.318	0.364	Brachypodium distachyon	https://mirbase.org/mature/MIMAT0035495
332	Sequence 3	UUAGGCUUCAGGUUCGUAG	miRNA	AGGAUUCUAGGCUUAGAGGU	20	45	0.556	-0.091	0.25	0.1	0.35	0.3	Capra hircus	https://mirbase.org/mature/MIMAT0036095
333	Sequence 3	UUAGGCUUCAGGUUCGUAG	miRNA	UGAGAACUGAAUUCCAUAGGCUUU	24	37.5	0.111	-0.067	0.292	0.167	0.208	0.333	Chrysemys picta;Ophiophagus hannah	https://mirbase.org/mature/MIMAT0037776;https://mirbase.org/mature/MIMAT0036729
334	Sequence 3	UUAGGCUUCAGGUUCGUAG	miRNA	AAUCAUAGGCUUGGAGAAUGGCA	23	43.48	0.4	0.231	0.348	0.13	0.304	0.217	Alligator mississippiensis	https://mirbase.org/mature/MIMAT0038361
335	Sequence 3	UUAGGCUUCAGGUUCGUAG	miRNA	UGAGAACUGAAUUCCAUAGGCUU	23	39.13	0.111	0	0.304	0.174	0.217	0.304	Python bivittatus	https://mirbase.org/mature/MIMAT0038961
336	Sequence 3	UUAGGCUUCAGGUUCGUAG	miRNA	UUAGGCUUUGUGGGACGUGAUU	22	45.45	0.6	-0.5	0.136	0.091	0.364	0.409	Glycine max	https://mirbase.org/mature/MIMAT0041729
337	Sequence 3	UUAGGCUUCAGGUUCGUAG	miRNA	UAGGCUUUAUCCAUGGUUGGAA	22	40.91	0.333	-0.231	0.227	0.136	0.273	0.364	Oreochromis niloticus	https://mirbase.org/mature/MIMAT0043475
338	Sequence 3	UUAGGCUUCAGGUUCGUAG	miRNA	CUUGUGGGUAAUAGGCUUUCCUUCCUUG	28	46.43	0.077	-0.6	0.107	0.214	0.25	0.429	Cucumis sativus	https://mirbase.org/mature/MIMAT0044622
339	Sequence 3	UUAGGCUUCAGGUUCGUAG	miRNA	UUGUACAUAGUAGGCUUUCAUU	22	31.82	0.143	-0.333	0.227	0.136	0.182	0.455	Cavia porcellus;Otolemur garnettii	https://mirbase.org/mature/MIMAT0047287;https://mirbase.org/mature/MIMAT0049679
340	Sequence 3	UUAGGCUUCAGGUUCGUAG	miRNA	UUGUACAUGGUAGGCUUUCACC	22	45.45	0	-0.333	0.182	0.227	0.227	0.364	Dasypus novemcinctus	https://mirbase.org/mature/MIMAT0047926
341	Sequence 3	UUAGGCUUCAGGUUCGUAG	miRNA	UUAGGCUUCCCCCUCCUCCUGC	22	63.64	-0.571	-0.75	0.045	0.5	0.136	0.318	Homo sapiens	https://mirbase.org/mature/MIMAT0049010
342	Sequence 3	UUAGGCUUCAGGUUCGUAG	piRNA	UGCUCUGGAUUAGGCUUUGGCUUUAGGG	28	50	0.429	-0.571	0.107	0.143	0.357	0.393	Homo sapiens	https://dashr2.lisanwanglab.org/get_bg_smrna.php?coord=chr1:24228250-24228277:pos&rnaID=piR-52914&dataSource=DASHR2_GEO_hg38;https://dashr2.lisanwanglab.org/get_bg_smrna.php?coord=chr3:153942746-153942773:pos&rnaID=piR-52914&dataSource=DASHR2_GEO_hg38
343	Sequence 3	UUAGGCUUCAGGUUCGUAG	piRNA	UAGGCUUUGUACCUGGGUGAUGAAAUAAUC	30	40	0.333	-0.111	0.267	0.133	0.267	0.333	Homo sapiens	https://dashr2.lisanwanglab.org/get_bg_smrna.php?coord=chr1:24795082-24795111:pos&rnaID=piR-39706&dataSource=DASHR2_GEO_hg38;https://dashr2.lisanwanglab.org/get_bg_smrna.php?coord=chr7:33104357-33104386:pos&rnaID=piR-39706&dataSource=DASHR2_GEO_hg38
344	Sequence 3	UUAGGCUUCAGGUUCGUAG	piRNA	UAGGCUUAGUACCUGGUGACAAAAUAAUCU	30	36.67	0.091	0.053	0.333	0.167	0.2	0.3	Homo sapiens	https://dashr2.lisanwanglab.org/get_bg_smrna.php?coord=chr10:104150819-104150848:neg&rnaID=piR-39701&dataSource=DASHR2_GEO_hg38;https://dashr2.lisanwanglab.org/get_bg_smrna.php?coord=chr13:25087081-25087110:neg&rnaID=piR-39701&dataSource=DASHR2_GEO_hg38;https://dashr2.lisanwanglab.org/get_bg_smrna.php?coord=chr1:173264031-173264060:pos&rnaID=piR-39701&dataSource=DASHR2_GEO_hg38;https://dashr2.lisanwanglab.org/get_bg_smrna.php?coord=chr6:16530654-16530683:pos&rnaID=piR-39701&dataSource=DASHR2_GEO_hg38;https://dashr2.lisanwanglab.org/get_bg_smrna.php?coord=chr8:74472026-74472055:neg&rnaID=piR-39701&dataSource=DASHR2_GEO_hg38
345	Sequence 3	UUAGGCUUCAGGUUCGUAG	piRNA	UGGAUAGGCUUAACACCCAGUCCUGUGC	28	53.57	-0.067	-0.077	0.214	0.286	0.25	0.25	Homo sapiens	https://dashr2.lisanwanglab.org/get_bg_smrna.php?coord=chr12:3748083-3748110:neg&rnaID=piR-54377&dataSource=DASHR2_GEO_hg38
346	Sequence 3	UUAGGCUUCAGGUUCGUAG	piRNA	UAGGCUUAGUACCUGGGUAAUGAAAUAGUC	30	40	0.333	0	0.3	0.133	0.267	0.3	Homo sapiens	https://dashr2.lisanwanglab.org/get_bg_smrna.php?coord=chr12:9329795-9329824:neg&rnaID=piR-39700&dataSource=DASHR2_GEO_hg38
347	Sequence 3	UUAGGCUUCAGGUUCGUAG	piRNA	UAGGCUUCCUGGACGUGACUGUGCUCU	27	55.56	0.067	-0.5	0.111	0.259	0.296	0.333	Homo sapiens	https://dashr2.lisanwanglab.org/get_bg_smrna.php?coord=chr12:122847250-122847276:neg&rnaID=piR-39703&dataSource=DASHR2_GEO_hg38
348	Sequence 3	UUAGGCUUCAGGUUCGUAG	piRNA	UUGGUAGGCUUGUUGUGGUCAAGGAAC	27	48.15	0.538	-0.286	0.185	0.111	0.37	0.333	Homo sapiens	https://dashr2.lisanwanglab.org/get_bg_smrna.php?coord=chr15:92610196-92610222:pos&rnaID=piR-61584&dataSource=DASHR2_GEO_hg38
349	Sequence 3	UUAGGCUUCAGGUUCGUAG	piRNA	UCGUAGGCUUACUUCACUCCAUAUCGAUCCC	31	48.39	-0.467	-0.25	0.194	0.355	0.129	0.323	Homo sapiens	https://dashr2.lisanwanglab.org/get_bg_smrna.php?coord=chr17:18396657-18396687:neg&rnaID=piR-45195&dataSource=DASHR2_GEO_hg38;https://dashr2.lisanwanglab.org/get_bg_smrna.php?coord=chr17:20547756-20547786:pos&rnaID=piR-45195&dataSource=DASHR2_GEO_hg38
350	Sequence 3	UUAGGCUUCAGGUUCGUAG	piRNA	GAGGUGCUGUAGGCUUGCAGGAACCCCGGU	30	63.33	0.263	-0.091	0.167	0.233	0.4	0.2	Homo sapiens	https://dashr2.lisanwanglab.org/get_bg_smrna.php?coord=chr19:34815795-34815824:neg&rnaID=piR-34838&dataSource=DASHR2_GEO_hg38
351	Sequence 3	UUAGGCUUCAGGUUCGUAG	piRNA	UACCAGAGGUGCUGUAGGCUUGCAGGAAC	29	55.17	0.25	0.077	0.241	0.207	0.345	0.207	Homo sapiens	https://dashr2.lisanwanglab.org/get_bg_smrna.php?coord=chr19:34815801-34815829:neg&rnaID=piR-38183&dataSource=DASHR2_GEO_hg38
352	Sequence 3	UUAGGCUUCAGGUUCGUAG	piRNA	UGUACCAGAGGUGCUGUAGGCUUGCAGGAA	30	53.33	0.375	0	0.233	0.167	0.367	0.233	Homo sapiens	https://dashr2.lisanwanglab.org/get_bg_smrna.php?coord=chr19:34815802-34815831:neg&rnaID=piR-57862&dataSource=DASHR2_GEO_hg38
353	Sequence 3	UUAGGCUUCAGGUUCGUAG	piRNA	UAGGCUUGCCAACUUGUUGAAUGAGAU	27	40.74	0.273	-0.125	0.259	0.148	0.259	0.333	Homo sapiens	https://dashr2.lisanwanglab.org/get_bg_smrna.php?coord=chr2:130044150-130044176:neg&rnaID=piR-39705&dataSource=DASHR2_GEO_hg38;https://dashr2.lisanwanglab.org/get_bg_smrna.php?coord=chr2:130434951-130434977:pos&rnaID=piR-39705&dataSource=DASHR2_GEO_hg38;https://dashr2.lisanwanglab.org/get_bg_smrna.php?coord=chr2:130686537-130686563:pos&rnaID=piR-39705&dataSource=DASHR2_GEO_hg38
354	Sequence 3	UUAGGCUUCAGGUUCGUAG	piRNA	UAGGCUUGCCAACUUGUUGAAUGAGA	26	42.31	0.273	-0.067	0.269	0.154	0.269	0.308	Homo sapiens	https://dashr2.lisanwanglab.org/get_bg_smrna.php?coord=chr2:130044151-130044176:neg&rnaID=piR-39704&dataSource=DASHR2_GEO_hg38;https://dashr2.lisanwanglab.org/get_bg_smrna.php?coord=chr2:130434952-130434977:pos&rnaID=piR-39704&dataSource=DASHR2_GEO_hg38;https://dashr2.lisanwanglab.org/get_bg_smrna.php?coord=chr2:130686537-130686562:pos&rnaID=piR-39704&dataSource=DASHR2_GEO_hg38;https://dashr2.lisanwanglab.org/get_bg_smrna.php?coord=chr2:131293232-131293257:pos&rnaID=piR-39704&dataSource=DASHR2_GEO_hg38
355	Sequence 3	UUAGGCUUCAGGUUCGUAG	piRNA	CCGCUGGGAAUACGGGUGCUGUAGGCUUA	31	61.29	0.158	-0.167	0.161	0.258	0.355	0.226	Homo sapiens	https://dashr2.lisanwanglab.org/get_bg_smrna.php?coord=chr6:4428053-4428083:pos&rnaID=piR-33151&dataSource=DASHR2_GEO_hg38
356	Sequence 3	UUAGGCUUCAGGUUCGUAG	piRNA	UCCCAGGAGGUAGCAACUAGGCUUUGGUUC	31	54.84	0.059	-0.143	0.194	0.258	0.29	0.258	Homo sapiens	https://dashr2.lisanwanglab.org/get_bg_smrna.php?coord=chr6:33917896-33917926:pos&rnaID=piR-43341&dataSource=DASHR2_GEO_hg38
357	Sequence 3	UUAGGCUUCAGGUUCGUAG	piRNA	UAGGCUUAGUACCUGGGUAAUGAAUGAGUC	30	46.67	0.429	-0.125	0.233	0.133	0.333	0.3	Homo sapiens	https://dashr2.lisanwanglab.org/get_bg_smrna.php?coord=chr5:32393784-32393813:pos&rnaID=piR-39698&dataSource=DASHR2_GEO_hg38
358	Sequence 3	UUAGGCUUCAGGUUCGUAG	piRNA	UGGUGGUCUAGGCUUCAGUGGAGUUG	26	53.85	0.571	-0.5	0.115	0.115	0.423	0.346	Homo sapiens	https://dashr2.lisanwanglab.org/get_bg_smrna.php?coord=chr3:39516753-39516778:pos&rnaID=piR-57406&dataSource=DASHR2_GEO_hg38;https://dashr2.lisanwanglab.org/get_bg_smrna.php?coord=chr5:62272109-62272134:pos&rnaID=piR-57406&dataSource=DASHR2_GEO_hg38;https://dashr2.lisanwanglab.org/get_bg_smrna.php?coord=chr6:86848557-86848582:pos&rnaID=piR-57406&dataSource=DASHR2_GEO_hg38
359	Sequence 4	UUCUCAGCCACUGUGCCA	siRNA	UUCUCUGUCAAUUCUCAGCC	19	47.37	-0.556	-0.4	0.158	0.368	0.105	0.368	Homo Sapiens	
360	Sequence 4	UUCUCAGCCACUGUGCCA	siRNA	UCUCAGCCGGCUGUCUCAGC	19	63.16	0	-0.714	0.053	0.316	0.316	0.316	Homo Sapiens	
361	Sequence 4	UUCUCAGCCACUGUGCCA	siRNA	UAGUCUCAGCCUCGGGUGA	19	52.63	0.2	-0.333	0.158	0.211	0.316	0.316	Homo Sapiens	
362	Sequence 4	UUCUCAGCCACUGUGCCA	siRNA	UUUCGAUUGAUCUCAGCCA	19	42.11	0	-0.273	0.211	0.211	0.211	0.368	Homo Sapiens	
363	Sequence 4	UUCUCAGCCACUGUGCCA	siRNA	AACAUCUCAGCCUGGGGUG	19	52.63	0	-0.111	0.211	0.263	0.263	0.263	Homo Sapiens	
364	Sequence 4	UUCUCAGCCACUGUGCCA	siRNA	UCUCAGCCAGCAUUGACUC	19	42.11	-0.5	-0.273	0.211	0.316	0.105	0.368	Homo Sapiens	
365	Sequence 4	UUCUCAGCCACUGUGCCA	siRNA	UUAUCUCAGCAUCAUACCC	19	42.11	-0.75	-0.091	0.263	0.368	0.053	0.316	Homo Sapiens	

	Sequence ID	siRNA Sequence	Type	Sequence	Length	GC %	GC skew	AT/AU Skew	MNC A	MNC C	MNC G	MNC U	Species	Link
366	Sequence 4	UUCUCAGCCACUGUUGCCA	siRNA	UCUGUCUCAGUCUCAGCCA	19	57.89	-0.273	-0.25	0.158	0.368	0.211	0.263	Homo Sapiens	
367	Sequence 4	UUCUCAGCCACUGUUGCCA	siRNA	UAUCUCAGCCUCCUUGCCG	19	57.89	-0.455	-0.5	0.105	0.421	0.158	0.316	Homo Sapiens	
368	Sequence 4	UUCUCAGCCACUGUUGCCA	siRNA	UCGUUCUCAGCUGAUCACC	19	52.63	-0.4	-0.333	0.158	0.368	0.158	0.316	Homo Sapiens	
369	Sequence 4	UUCUCAGCCACUGUUGCCA	siRNA	AUUCUCAGCCUCAUUUACCG	19	42.11	-0.5	-0.273	0.211	0.316	0.105	0.368	Homo Sapiens	
370	Sequence 4	UUCUCAGCCACUGUUGCCA	siRNA	UUUGGGUCAAUCUCAGCCA	19	47.37	-0.111	-0.2	0.211	0.263	0.211	0.316	Homo Sapiens	
371	Sequence 4	UUCUCAGCCACUGUUGCCA	siRNA	UUGGAUUCAGCAUUAAUGCG	19	42.11	0	0.091	0.316	0.211	0.211	0.263	Homo Sapiens	
372	Sequence 4	UUCUCAGCCACUGUUGCCA	siRNA	AAUCUCAGCUACUCAAUCG	19	42.11	-0.5	0.091	0.316	0.316	0.105	0.263	Homo Sapiens	
373	Sequence 4	UUCUCAGCCACUGUUGCCA	siRNA	UGACGUGACACUUCUCAGC	19	52.63	-0.2	-0.111	0.211	0.316	0.211	0.263	Homo Sapiens	
374	Sequence 4	UUCUCAGCCACUGUUGCCA	siRNA	CUAUUAUGGACUCUCAGC	19	47.37	-0.333	-0.2	0.211	0.316	0.158	0.316	Homo Sapiens	
375	Sequence 4	UUCUCAGCCACUGUUGCCA	siRNA	UAAAUCUCAGCAACAUCGA	19	36.84	-0.429	0.333	0.421	0.263	0.105	0.211	Homo Sapiens	
376	Sequence 4	UUCUCAGCCACUGUUGCCA	siRNA	UCUCUCAGCAUUUCCAGUU	19	42.11	-0.5	-0.455	0.158	0.316	0.105	0.421	Homo Sapiens	
377	Sequence 4	UUCUCAGCCACUGUUGCCA	siRNA	GUGAUAUUAACUCUCAGC	19	36.84	-0.143	-0.167	0.263	0.158	0.211	0.368	Homo Sapiens	
378	Sequence 4	UUCUCAGCCACUGUUGCCA	siRNA	AAGUAAUCCAUCUCAGCCG	19	47.37	-0.333	0.2	0.316	0.316	0.158	0.211	Homo Sapiens	
379	Sequence 4	UUCUCAGCCACUGUUGCCA	siRNA	UAUCGUUCCUUAUCUCAGC	19	42.11	-0.5	-0.455	0.158	0.316	0.105	0.421	Homo Sapiens	
380	Sequence 4	UUCUCAGCCACUGUUGCCA	siRNA	UAACUGCUAAGCUCUCAGC	19	47.37	-0.333	0	0.263	0.316	0.158	0.263	Homo Sapiens	
381	Sequence 4	UUCUCAGCCACUGUUGCCA	siRNA	ACAUAAGGGACCUCUCAGC	19	52.63	-0.2	0.333	0.316	0.316	0.211	0.158	Homo Sapiens	
382	Sequence 4	UUCUCAGCCACUGUUGCCA	siRNA	AUGCCGUGUAGCUCUCAGC	19	57.89	-0.091	-0.25	0.158	0.316	0.263	0.263	Homo Sapiens	
383	Sequence 4	UUCUCAGCCACUGUUGCCA	siRNA	UCUCAGCCAAUCUCCGAUC	19	52.63	-0.6	-0.111	0.211	0.421	0.105	0.263	Homo Sapiens	
384	Sequence 4	UUCUCAGCCACUGUUGCCA	siRNA	UUACAUCUCAGCGAUCACA	19	42.11	-0.5	0.091	0.316	0.316	0.105	0.263	Homo Sapiens	
385	Sequence 4	UUCUCAGCCACUGUUGCCA	siRNA	UUUCGUCUCAGCACUGCAC	19	52.63	-0.4	-0.333	0.158	0.368	0.158	0.316	Homo Sapiens	
386	Sequence 4	UUCUCAGCCACUGUUGCCA	siRNA	UCUCUCAGCCGGUAAAUUC	19	47.37	-0.333	-0.2	0.211	0.316	0.158	0.316	Homo Sapiens	
387	Sequence 4	UUCUCAGCCACUGUUGCCA	siRNA	UCGUUCUUCAGGUCUCAGC	19	52.63	-0.2	-0.556	0.105	0.316	0.211	0.368	Homo Sapiens	
388	Sequence 4	UUCUCAGCCACUGUUGCCA	siRNA	UAUCUCAGCUUCUCUGGAC	19	47.37	-0.333	-0.4	0.158	0.316	0.158	0.368	Homo Sapiens	
389	Sequence 4	UUCUCAGCCACUGUUGCCA	siRNA	UCACUUGAACUCUCAGCUG	19	47.37	-0.333	-0.2	0.211	0.316	0.158	0.316	Homo Sapiens	
390	Sequence 4	UUCUCAGCCACUGUUGCCA	siRNA	AUGUCUCUCAGCUAUGAUU	19	36.84	-0.143	-0.333	0.211	0.211	0.158	0.421	Homo Sapiens	
391	Sequence 4	UUCUCAGCCACUGUUGCCA	siRNA	UAAGUAUUGUUCUCAGCUG	19	36.84	0.143	-0.333	0.211	0.158	0.211	0.421	Homo Sapiens	
392	Sequence 4	UUCUCAGCCACUGUUGCCA	siRNA	UUCGGCACCAAGUCUCAGC	19	57.89	-0.273	0	0.211	0.368	0.211	0.211	Homo Sapiens	
393	Sequence 4	UUCUCAGCCACUGUUGCCA	siRNA	UCUCUCAGCAUAAUCCCAC	19	47.37	-0.778	0	0.263	0.421	0.053	0.263	Homo Sapiens	
394	Sequence 4	UUCUCAGCCACUGUUGCCA	siRNA	UGAUGCAGCACUUCUCAGC	19	52.63	-0.2	-0.111	0.211	0.316	0.211	0.263	Homo Sapiens	
395	Sequence 4	UUCUCAGCCACUGUUGCCA	siRNA	GAAGGUCUCAGCGUUGGUC	19	57.89	0.273	-0.25	0.158	0.211	0.368	0.263	Homo Sapiens	
396	Sequence 4	UUCUCAGCCACUGUUGCCA	siRNA	UGUGUGACGGCCUCUCAGC	19	63.16	0	-0.429	0.105	0.316	0.316	0.263	Homo Sapiens	
397	Sequence 4	UUCUCAGCCACUGUUGCCA	siRNA	UUUGAAGUCAUCUCAGCUC	19	42.11	-0.25	-0.273	0.211	0.263	0.158	0.368	Homo Sapiens	
398	Sequence 4	UUCUCAGCCACUGUUGCCA	siRNA	GUCGUCUCAGCUCAUCUGC	19	57.89	-0.273	-0.5	0.105	0.368	0.211	0.316	Homo Sapiens	
399	Sequence 4	UUCUCAGCCACUGUUGCCA	siRNA	UAAGGUCUCAGCUAGUUUC	19	42.11	0	-0.273	0.211	0.211	0.211	0.368	Homo Sapiens	
400	Sequence 4	UUCUCAGCCACUGUUGCCA	siRNA	GAAUUCUCAGCCCUCUUCA	19	47.37	-0.556	-0.2	0.211	0.368	0.105	0.316	Homo Sapiens	
401	Sequence 4	UUCUCAGCCACUGUUGCCA	siRNA	UUCCAAACAGGGUCUCAGC	19	52.63	-0.2	0.111	0.263	0.316	0.211	0.211	Homo Sapiens	
402	Sequence 4	UUCUCAGCCACUGUUGCCA	siRNA	AGUCGCCAAACUUCUCAGC	19	52.63	-0.4	0.111	0.263	0.368	0.158	0.211	Homo Sapiens	
403	Sequence 4	UUCUCAGCCACUGUUGCCA	siRNA	UGGCACAGGAUCUCAGCGG	19	63.16	0.167	0.143	0.211	0.263	0.368	0.158	Homo Sapiens	
404	Sequence 4	UUCUCAGCCACUGUUGCCA	siRNA	UCGUCCACAGUCUCAGCCU	19	57.89	-0.455	-0.25	0.158	0.421	0.158	0.263	Homo Sapiens	
405	Sequence 4	UUCUCAGCCACUGUUGCCA	siRNA	UGUCUCAGCAGCACUUCUG	19	52.63	-0.2	-0.333	0.158	0.316	0.211	0.316	Homo Sapiens	
406	Sequence 4	UUCUCAGCCACUGUUGCCA	siRNA	AAGUUGAACAUCUCAGCUU	19	36.84	-0.143	0	0.316	0.211	0.158	0.316	Homo Sapiens	
407	Sequence 4	UUCUCAGCCACUGUUGCCA	siRNA	AAAGUGUGGUAUUCUCAGC	19	42.11	0.25	-0.091	0.263	0.158	0.263	0.316	Homo Sapiens	
408	Sequence 4	UUCUCAGCCACUGUUGCCA	siRNA	AGAUCUCUCAGCGUGUUGC	19	52.63	0	-0.333	0.158	0.263	0.263	0.316	Homo Sapiens	
409	Sequence 4	UUCUCAGCCACUGUUGCCA	siRNA	UGUGAUCUCAGCAGCGUUC	19	52.63	0	-0.333	0.158	0.263	0.263	0.316	Homo Sapiens	
410	Sequence 4	UUCUCAGCCACUGUUGCCA	siRNA	AAGUCGACACUCUCAGCUC	19	52.63	-0.4	0.111	0.263	0.368	0.158	0.211	Homo Sapiens	
411	Sequence 4	UUCUCAGCCACUGUUGCCA	siRNA	UUCAUCUCAGCCUUUACCG	19	47.37	-0.556	-0.4	0.158	0.368	0.105	0.368	Homo Sapiens	
412	Sequence 4	UUCUCAGCCACUGUUGCCA	siRNA	AAUCUCAGCAUCCAGCUUA	19	42.11	-0.5	0.091	0.316	0.316	0.105	0.263	Homo Sapiens	
413	Sequence 4	UUCUCAGCCACUGUUGCCA	siRNA	UUAGCCGUCUCAGCUUCUG	19	52.63	-0.2	-0.556	0.105	0.316	0.211	0.368	Homo Sapiens	
414	Sequence 4	UUCUCAGCCACUGUUGCCA	siRNA	AUGUUCUCAGCGUGCUCCC	19	57.89	-0.273	-0.5	0.105	0.368	0.211	0.316	Homo Sapiens	
415	Sequence 4	UUCUCAGCCACUGUUGCCA	siRNA	AUCUCAGCCACUUUCUUGU	19	42.11	-0.5	-0.455	0.158	0.316	0.105	0.421	Homo Sapiens	
416	Sequence 4	UUCUCAGCCACUGUUGCCA	siRNA	UCUCAGCAUACUUAUACCC	19	42.11	-0.75	-0.091	0.263	0.368	0.053	0.316	Homo Sapiens	
417	Sequence 4	UUCUCAGCCACUGUUGCCA	siRNA	AAGCGUUGAAGAUCUCAGC	19	47.37	0.111	0.2	0.316	0.211	0.263	0.211	Homo Sapiens	
418	Sequence 4	UUCUCAGCCACUGUUGCCA	siRNA	AGGUUCUCAGCCGAUGUGU	19	52.63	0.2	-0.333	0.158	0.211	0.316	0.316	Homo Sapiens	
419	Sequence 4	UUCUCAGCCACUGUUGCCA	siRNA	AAACCCGUCAGUCUCAGCC	19	57.89	-0.455	0.25	0.263	0.421	0.158	0.158	Homo Sapiens	
420	Sequence 4	UUCUCAGCCACUGUUGCCA	siRNA	UUGGUCUCAGCUUUAUCAG	19	42.11	0	-0.455	0.158	0.211	0.211	0.421	Homo Sapiens	
421	Sequence 4	UUCUCAGCCACUGUUGCCA	siRNA	AUUCGUUGGUCCUCUCAGC	19	52.63	-0.2	-0.556	0.105	0.316	0.211	0.368	Homo Sapiens	
422	Sequence 4	UUCUCAGCCACUGUUGCCA	siRNA	UAUCAGCCCAGUUCUCAGC	19	52.63	-0.4	-0.111	0.211	0.368	0.158	0.263	Homo Sapiens	
423	Sequence 4	UUCUCAGCCACUGUUGCCA	siRNA	GUAUCGGGCAGUUCUCAGC	19	57.89	0.091	-0.25	0.158	0.263	0.316	0.263	Homo Sapiens	
424	Sequence 4	UUCUCAGCCACUGUUGCCA	siRNA	UUGCCGUCAUAUUCUCAGC	19	47.37	-0.333	-0.4	0.158	0.316	0.158	0.368	Homo Sapiens	
425	Sequence 4	UUCUCAGCCACUGUUGCCA	siRNA	UCCGCCACGAUCUCAGCUC	19	63.16	-0.5	-0.143	0.158	0.474	0.158	0.211	Homo Sapiens	
426	Sequence 4	UUCUCAGCCACUGUUGCCA	siRNA	UAGAUCUCAGCAUUAGCGU	19	42.11	0	-0.091	0.263	0.211	0.211	0.316	Homo Sapiens	
427	Sequence 4	UUCUCAGCCACUGUUGCCA	siRNA	CUUCUCAGCUUCUAUCUUC	19	42.11	-0.75	-0.636	0.105	0.368	0.053	0.474	Homo Sapiens	
428	Sequence 4	UUCUCAGCCACUGUUGCCA	siRNA	AACCUGUCGAAUCUCAGCC	19	52.63	-0.4	0.111	0.263	0.368	0.158	0.211	Homo Sapiens	
429	Sequence 4	UUCUCAGCCACUGUUGCCA	siRNA	AUGUCGUCCAUCUCAGCCC	19	57.89	-0.455	-0.25	0.158	0.421	0.158	0.263	Homo Sapiens	
430	Sequence 4	UUCUCAGCCACUGUUGCCA	siRNA	UAUUGGUUCUCAGCAUCGC	19	47.37	-0.111	-0.4	0.158	0.263	0.211	0.368	Homo Sapiens	
431	Sequence 4	UUCUCAGCCACUGUUGCCA	siRNA	UAAACCGGACUCUCAGCCC	19	57.89	-0.455	0.25	0.263	0.421	0.158	0.158	Homo Sapiens	
432	Sequence 4	UUCUCAGCCACUGUUGCCA	siRNA	UCAUCCGUGGUCUCAGCGG	19	63.16	0	-0.429	0.105	0.316	0.316	0.263	Homo Sapiens	
433	Sequence 4	UUCUCAGCCACUGUUGCCA	siRNA	UGGCUCUCAGCAUAUUACA	19	42.11	-0.25	-0.091	0.263	0.263	0.158	0.316	Homo Sapiens	
434	Sequence 4	UUCUCAGCCACUGUUGCCA	siRNA	UUGAUCUCAGCAAUCUCCU	19	42.11	-0.5	-0.273	0.211	0.316	0.105	0.368	Homo Sapiens	
435	Sequence 4	UUCUCAGCCACUGUUGCCA	siRNA	AAUCUCUCAGCCUUAGUUC	19	42.11	-0.5	-0.273	0.211	0.316	0.105	0.368	Homo Sapiens	
436	Sequence 4	UUCUCAGCCACUGUUGCCA	siRNA	UAAUGUCUCAGCGGGUCCG	19	57.89	0.091	-0.25	0.158	0.263	0.316	0.263	Homo Sapiens	
437	Sequence 4	UUCUCAGCCACUGUUGCCA	siRNA	UCGAUCUCAGCCAUACCAG	19	52.63	-0.4	0.111	0.263	0.368	0.158	0.211	Homo Sapiens	
438	Sequence 4	UUCUCAGCCACUGUUGCCA	siRNA	CUUGCGAAGAGUUCUCAGC	19	52.63	0	-0.111	0.211	0.263	0.263	0.263	Homo Sapiens	
439	Sequence 4	UUCUCAGCCACUGUUGCCA	siRNA	AUAAUCUCUAUCUCAGCAC	19	36.84	-0.714	0	0.316	0.316	0.053	0.316	Homo Sapiens	
440	Sequence 4	UUCUCAGCCACUGUUGCCA	siRNA	GAGUCCGCGUGUUCUCAGC	19	63.16	0	-0.429	0.105	0.316	0.316	0.263	Homo Sapiens	
441	Sequence 4	UUCUCAGCCACUGUUGCCA	siRNA	UUCUUCGAAGUCUCUCAGC	19	47.37	-0.333	-0.4	0.158	0.316	0.158	0.368	Homo Sapiens	
442	Sequence 4	UUCUCAGCCACUGUUGCCA	siRNA	UUGAACUCUUACUCUCAGC	19	42.11	-0.5	-0.273	0.211	0.316	0.105	0.368	Homo Sapiens	
443	Sequence 4	UUCUCAGCCACUGUUGCCA	siRNA	UUAUCUCUCAGCUCCACGC	19	52.63	-0.6	-0.333	0.158	0.421	0.105	0.316	Homo Sapiens	
444	Sequence 4	UUCUCAGCCACUGUUGCCA	siRNA	UCACUCUCAGCCCCUUUGA	19	52.63	-0.6	-0.333	0.158	0.421	0.105	0.316	Homo Sapiens	
445	Sequence 4	UUCUCAGCCACUGUUGCCA	siRNA	UCUCUCAGCUGUUCGUAGC	19	52.63	-0.2	-0.556	0.105	0.316	0.211	0.368	Homo Sapiens	
446	Sequence 4	UUCUCAGCCACUGUUGCCA	siRNA	UAUAUCGGCUCCUCUCAGC	19	52.63	-0.4	-0.333	0.158	0.368	0.158	0.316	Homo Sapiens	
447	Sequence 4	UUCUCAGCCACUGUUGCCA	siRNA	AACCUCUCAGCUAUUCAGG	19	47.37	-0.333	0	0.263	0.316	0.158	0.263	Homo Sapiens	
448	Sequence 4	UUCUCAGCCACUGUUGCCA	siRNA	UAGUUGUAAAUCUCAGCGU	19	36.84	0.143	-0.167	0.263	0.158	0.211	0.368	Homo Sapiens	
449	Sequence 4	UUCUCAGCCACUGUUGCCA	siRNA	UGAAUCACAAUCUCAGCUA	19	36.84	-0.429	0.167	0.368	0.263	0.105	0.263	Homo Sapiens	
450	Sequence 4	UUCUCAGCCACUGUUGCCA	siRNA	UUUGGUCUCAGCAGUCUCC	19	52.63	-0.2	-0.556	0.105	0.316	0.211	0.368	Homo Sapiens	
451	Sequence 4	UUCUCAGCCACUGUUGCCA	siRNA	UUGAUCUCAGCCCACAAGC	19	52.63	-0.4	0.111	0.263	0.368	0.158	0.211	Homo Sapiens	
452	Sequence 4	UUCUCAGCCACUGUUGCCA	siRNA	UAUUUCUCAGCCACUUCGA	19	42.11	-0.5	-0.273	0.211	0.316	0.105	0.368	Homo Sapiens	
453	Sequence 4	UUCUCAGCCACUGUUGCCA	siRNA	AAUUUGCGCAUCUCAGCAG	19	47.37	-0.111	0	0.263	0.263	0.211	0.263	Homo Sapiens	
454	Sequence 4	UUCUCAGCCACUGUUGCCA	siRNA	AACAUCAUAGCCUCUCAGC	19	47.37	-0.556	0.2	0.316	0.368	0.105	0.211	Homo Sapiens	
455	Sequence 4	UUCUCAGCCACUGUUGCCA	siRNA	AUCUCAGCAAGGUCUAAGG	19	47.37	0.111	0.2	0.316	0.211	0.263	0.211	Homo Sapiens	
456	Sequence 4	UUCUCAGCCACUGUUGCCA	siRNA	UAUACAGCUUUCUCUCAGC	19	42.11	-0.5	-0.273	0.211	0.316	0.105	0.368	Homo Sapiens	
457	Sequence 4	UUCUCAGCCACUGUUGCCA	siRNA	UAAGAUACCACUUCUCAGC	19	42.11	-0.5	0.091	0.316	0.316	0.105	0.263	Homo Sapiens	
458	Sequence 4	UUCUCAGCCACUGUUGCCA	siRNA	CCUAUUCUCAGCUCGAUCC	19	52.63	-0.6	-0.333	0.158	0.421	0.105	0.316	Homo Sapiens	
459	Sequence 4	UUCUCAGCCACUGUUGCCA	siRNA	UCUCAGCAAGCUUAUUACA	19	36.84	-0.429	0	0.316	0.263	0.105	0.316	Homo Sapiens	
460	Sequence 4	UUCUCAGCCACUGUUGCCA	siRNA	AGACGCUUCUAAUCUCAGC	19	47.37	-0.333	0	0.263	0.316	0.158	0.263	Homo Sapiens	
461	Sequence 4	UUCUCAGCCACUGUUGCCA	siRNA	UAAAUCUCAGCAGAAGUCG	19	42.11	0	0.273	0.368	0.211	0.211	0.211	Homo Sapiens	
462	Sequence 4	UUCUCAGCCACUGUUGCCA	siRNA	UUUCUCAGCAGCAAUGGUG	19	47.37	0.111	-0.2	0.211	0.211	0.263	0.316	Homo Sapiens	
463	Sequence 4	UUCUCAGCCACUGUUGCCA	siRNA	UAUUCCAGGAUCUCAGCUU	19	42.11	-0.25	-0.273	0.211	0.263	0.158	0.368	Homo Sapiens	
464	Sequence 4	UUCUCAGCCACUGUUGCCA	siRNA	GAUCUCAGCAUAGACGCGC	19	57.89	-0.091	0.25	0.263	0.316	0.263	0.158	Homo Sapiens	
465	Sequence 4	UUCUCAGCCACUGUUGCCA	siRNA	GAUUUCUUGAUCUCAGCUU	19	36.84	-0.143	-0.5	0.158	0.211	0.158	0.474	Homo Sapiens	
466	Sequence 4	UUCUCAGCCACUGUUGCCA	siRNA	UUACUCUCAGCACAUUGGA	19	42.11	-0.25	-0.091	0.263	0.263	0.158	0.316	Homo Sapiens	
467	Sequence 4	UUCUCAGCCACUGUUGCCA	siRNA	CGAUCUCUCAGCAAACUCA	19	47.37	-0.556	0.2	0.316	0.368	0.105	0.211	Homo Sapiens	
468	Sequence 4	UUCUCAGCCACUGUUGCCA	siRNA	UACUUCUCAGCCAGUGUGC	19	52.63	-0.2	-0.333	0.158	0.316	0.211	0.316	Homo Sapiens	
469	Sequence 4	UUCUCAGCCACUGUUGCCA	siRNA	UGUCAUCUCAGCUUUAAUG	19	36.84	-0.143	-0.333	0.211	0.211	0.158	0.421	Homo Sapiens	
470	Sequence 4	UUCUCAGCCACUGUUGCCA	siRNA	UUAUUCUCAGCCGCAGUGC	19	52.63	-0.2	-0.333	0.158	0.316	0.211	0.316	Homo Sapiens	
471	Sequence 4	UUCUCAGCCACUGUUGCCA	siRNA	CAAAGCGUAACAUCUCAGC	19	47.37	-0.333	0.4	0.368	0.316	0.158	0.158	Homo Sapiens	
472	Sequence 4	UUCUCAGCCACUGUUGCCA	siRNA	UUGGAGUGAACUUCUCAGC	19	47.37	0.111	-0.2	0.211	0.211	0.263	0.316	Homo Sapiens	
473	Sequence 4	UUCUCAGCCACUGUUGCCA	siRNA	AUUUGCGCGAUCUCAGCUC	19	52.63	-0.2	-0.333	0.158	0.316	0.211	0.316	Homo Sapiens	
474	Sequence 4	UUCUCAGCCACUGUUGCCA	siRNA	AAUCUCAGCCUCCGUCUUC	19	52.63	-0.6	-0.333	0.158	0.421	0.105	0.316	Homo Sapiens	
475	Sequence 4	UUCUCAGCCACUGUUGCCA	siRNA	CAAGCCGCGAAUUCUCAGC	19	57.89	-0.273	0.25	0.263	0.368	0.211	0.158	Homo Sapiens	
476	Sequence 4	UUCUCAGCCACUGUUGCCA	siRNA	UAGUCUUUGAUCUCAGCAG	19	42.11	0	-0.273	0.211	0.211	0.211	0.368	Homo Sapiens	
477	Sequence 4	UUCUCAGCCACUGUUGCCA	siRNA	AAUCUAUCAUCUCAGCUCG	19	42.11	-0.5	-0.091	0.263	0.316	0.105	0.316	Homo Sapiens	
478	Sequence 4	UUCUCAGCCACUGUUGCCA	siRNA	UUCCACGCAGCUUCUCAGC	19	57.89	-0.455	-0.25	0.158	0.421	0.158	0.263	Homo Sapiens	
479	Sequence 4	UUCUCAGCCACUGUUGCCA	siRNA	ACGAACUUUCUCAGCCCCG	19	57.89	-0.455	0	0.211	0.421	0.158	0.211	Homo Sapiens	
480	Sequence 4	UUCUCAGCCACUGUUGCCA	siRNA	GAACUUUCUCAGCCCCGGG	19	63.16	-0.167	-0.143	0.158	0.368	0.263	0.211	Homo Sapiens	
481	Sequence 4	UUCUCAGCCACUGUUGCCA	siRNA	UCUCAGCCCCGGGAUCACU	19	63.16	-0.333	-0.143	0.158	0.421	0.211	0.211	Homo Sapiens	
482	Sequence 4	UUCUCAGCCACUGUUGCCA	siRNA	UAGUGAAGUUCUCAGCUUC	19	42.11	0	-0.273	0.211	0.211	0.211	0.368	Homo Sapiens	
483	Sequence 4	UUCUCAGCCACUGUUGCCA	siRNA	CUGCCUCUCAGCGGAUGUA	19	57.89	-0.091	-0.25	0.158	0.316	0.263	0.263	Homo Sapiens	
484	Sequence 4	UUCUCAGCCACUGUUGCCA	miRNA	UGUAAACAUCCUACACUCUCAGC	23	43.48	-0.6	0.077	0.304	0.348	0.087	0.261	Homo sapiens;Mus musculus;Rattus norvegicus;Sus scrofa;Pan troglodytes;Lagothrix lagotricha;Macaca nemestrina;Xenopus tropicalis;Bos taurus;Ornithorhynchus anatinus;Macaca mulatta;Equus caballus;Pongo pygmaeus;Anolis carolinensis;Oryzias latipes;Cricetulus griseus;Gorilla gorilla;Monodelphis domestica;Salmo salar;Alligator mississippiensis;Capra hircus;Tupaia chinensis;Astatotilapia burtoni;Microcebus murinus;Papio hamadryas;Otolemur garnettii	https://mirbase.org/mature/MIMAT0000244;https://mirbase.org/mature/MIMAT0000514;https://mirbase.org/mature/MIMAT0000804;https://mirbase.org/mature/MIMAT0002167;https://mirbase.org/mature/MIMAT0002614;https://mirbase.org/mature/MIMAT0002615;https://mirbase.org/mature/MIMAT0002616;https://mirbase.org/mature/MIMAT0003577;https://mirbase.org/mature/MIMAT0003850;https://mirbase.org/mature/MIMAT0006911;https://mirbase.org/mature/MIMAT0006170;https://mirbase.org/mature/MIMAT0012915;https://mirbase.org/mature/MIMAT0015737;https://mirbase.org/mature/MIMAT0021924;https://mirbase.org/mature/MIMAT0022598;https://mirbase.org/mature/MIMAT0037458;https://mirbase.org/mature/MIMAT0024111;https://mirbase.org/mature/MIMAT0028502;https://mirbase.org/mature/MIMAT0032600;https://mirbase.org/mature/MIMAT0038081;https://mirbase.org/mature/MIMAT0036125;https://mirbase.org/mature/MIMAT0036585;https://mirbase.org/mature/MIMAT0042071;https://mirbase.org/
485	Sequence 4	UUCUCAGCCACUGUUGCCA	miRNA	UAAUCUCAGCUGGCAACUGUGA	22	45.45	0	0	0.273	0.227	0.227	0.273	Homo sapiens;Mus musculus;Rattus norvegicus;Danio rerio;Ornithorhynchus anatinus;Macaca mulatta;Bos taurus;Equus caballus;Taeniopygia guttata;Anolis carolinensis;Ictalurus punctatus;Salmo salar;Chrysemys picta;Columba livia;Python bivittatus;Callithrix jacchus	https://mirbase.org/mature/MIMAT0000273;https://mirbase.org/mature/MIMAT0000662;https://mirbase.org/mature/MIMAT0000886;https://mirbase.org/mature/MIMAT0001284;https://mirbase.org/mature/MIMAT0007223;https://mirbase.org/mature/MIMAT0006242;https://mirbase.org/mature/MIMAT0009265;https://mirbase.org/mature/MIMAT0013072;https://mirbase.org/mature/MIMAT0014576;https://mirbase.org/mature/MIMAT0021875;https://mirbase.org/mature/MIMAT0029467;https://mirbase.org/mature/MIMAT0032518;https://mirbase.org/mature/MIMAT0037843;https://mirbase.org/mature/MIMAT0038596;https://mirbase.org/mature/MIMAT0039022;https://mirbase.org/mature/MIMAT0039428
486	Sequence 4	UUCUCAGCCACUGUUGCCA	miRNA	UAAUCUCAGCUGGCAACUGUG	21	47.62	0	-0.091	0.238	0.238	0.238	0.286	Gallus gallus;Lemur catta;Gorilla gorilla;Pongo pygmaeus;Pan paniscus;Xenopus tropicalis;Monodelphis domestica;Canis familiaris;Sarcophilus harrisii;Alligator mississippiensis	https://mirbase.org/mature/MIMAT0001131;https://mirbase.org/mature/MIMAT0002558;https://mirbase.org/mature/MIMAT0002560;https://mirbase.org/mature/MIMAT0002561;https://mirbase.org/mature/MIMAT0002562;https://mirbase.org/mature/MIMAT0003629;https://mirbase.org/mature/MIMAT0004131;https://mirbase.org/mature/MIMAT0009849;https://mirbase.org/mature/MIMAT0022777;https://mirbase.org/mature/MIMAT0038242
487	Sequence 4	UUCUCAGCCACUGUUGCCA	miRNA	UGUAAACAUCCUACACUCUCAGCU	24	41.67	-0.6	0	0.292	0.333	0.083	0.292	Gallus gallus;Canis familiaris;Taeniopygia guttata;Sarcophilus harrisii;Ictalurus punctatus;Chrysemys picta;Columba livia;Python bivittatus;Ophiophagus hannah;Pteropus alecto;Metriaclima zebra;Neolamprologus brichardi;Oreochromis niloticus;Pundamilia nyererei;Gadus morhua;Cavia porcellus;Dasypus novemcinctus;Oryctolagus cuniculus;Callithrix jacchus;Nomascus leucogenys	https://mirbase.org/mature/MIMAT0001137;https://mirbase.org/mature/MIMAT0006605;https://mirbase.org/mature/MIMAT0014521;https://mirbase.org/mature/MIMAT0022808;https://mirbase.org/mature/MIMAT0029498;https://mirbase.org/mature/MIMAT0037678;https://mirbase.org/mature/MIMAT0038453;https://mirbase.org/mature/MIMAT0038873;https://mirbase.org/mature/MIMAT0036892;https://mirbase.org/mature/MIMAT0039880;https://mirbase.org/mature/MIMAT0042314;https://mirbase.org/mature/MIMAT0042507;https://mirbase.org/mature/MIMAT0042674;https://mirbase.org/mature/MIMAT0042878;https://mirbase.org/mature/MIMAT0044096;https://mirbase.org/mature/MIMAT0046907;https://mirbase.org/mature/MIMAT0047560;https://mirbase.org/mature/MIMAT0048154;https://mirbase.org/mature/MIMAT0049309;https://mirbase.org/mature/MIMAT0049402
488	Sequence 4	UUCUCAGCCACUGUUGCCA	miRNA	UAAUCUCAGCUGGCAACUGUGAG	23	47.83	0.091	0	0.261	0.217	0.261	0.261	Sus scrofa;Branchiostoma floridae;Branchiostoma belcheri;Ophiophagus hannah;Xenopus laevis;Cavia porcellus;Dasypus novemcinctus;Oryctolagus cuniculus	https://mirbase.org/mature/MIMAT0002130;https://mirbase.org/mature/MIMAT0009495;https://mirbase.org/mature/MIMAT0031646;https://mirbase.org/mature/MIMAT0036833;https://mirbase.org/mature/MIMAT0046536;https://mirbase.org/mature/MIMAT0047119;https://mirbase.org/mature/MIMAT0047762;https://mirbase.org/mature/MIMAT0048341
489	Sequence 4	UUCUCAGCCACUGUUGCCA	miRNA	UUAAUCUCAGCUGGCAACUGU	21	42.86	-0.111	-0.167	0.238	0.238	0.19	0.333	Pan troglodytes	https://mirbase.org/mature/MIMAT0002559
490	Sequence 4	UUCUCAGCCACUGUUGCCA	miRNA	AAAUCUCAGCUGGCAACUGUGA	22	45.45	0	0.167	0.318	0.227	0.227	0.227	Fugu rubripes;Tetraodon nigroviridis;Astatotilapia burtoni;Metriaclima zebra;Neolamprologus brichardi;Oreochromis niloticus;Pundamilia nyererei;Gadus morhua	https://mirbase.org/mature/MIMAT0002973;https://mirbase.org/mature/MIMAT0002974;https://mirbase.org/mature/MIMAT0042191;https://mirbase.org/mature/MIMAT0042410;https://mirbase.org/mature/MIMAT0042596;https://mirbase.org/mature/MIMAT0042796;https://mirbase.org/mature/MIMAT0042982;https://mirbase.org/mature/MIMAT0044329
491	Sequence 4	UUCUCAGCCACUGUUGCCA	miRNA	UAAUCUCAGCUGGCAAUCUGUGA	23	43.48	0	-0.077	0.261	0.217	0.217	0.304	Ciona intestinalis;Ciona savignyi	https://mirbase.org/mature/MIMAT0006111;https://mirbase.org/mature/MIMAT0006134
492	Sequence 4	UUCUCAGCCACUGUUGCCA	miRNA	UCUCAGCUACAUCGGUGUAAAUC	23	43.48	-0.2	-0.077	0.261	0.261	0.174	0.304	Gallus gallus	https://mirbase.org/mature/MIMAT0007345
493	Sequence 4	UUCUCAGCCACUGUUGCCA	miRNA	UGUAAACACCCUACACUCUCAGCU	24	45.83	-0.636	0.077	0.292	0.375	0.083	0.25	Bos taurus	https://mirbase.org/mature/MIMAT0009282
494	Sequence 4	UUCUCAGCCACUGUUGCCA	miRNA	UAAUCUCAGCUGGUAAUUCUGAG	23	39.13	0.111	-0.143	0.261	0.174	0.217	0.348	Lottia gigantea;Melibe leonina	https://mirbase.org/mature/MIMAT0009588;https://mirbase.org/mature/MIMAT0048769
495	Sequence 4	UUCUCAGCCACUGUUGCCA	miRNA	CAAUCUCAGCCUGUUAAUAGGAGUU	25	40	0	-0.067	0.28	0.2	0.2	0.32	Schmidtea mediterranea	https://mirbase.org/mature/MIMAT0011245
496	Sequence 4	UUCUCAGCCACUGUUGCCA	miRNA	AUUGGUUUCUAGAUCUCAGCA	21	38.1	0	-0.231	0.238	0.19	0.19	0.381	Acyrthosiphon pisum	https://mirbase.org/mature/MIMAT0014773
497	Sequence 4	UUCUCAGCCACUGUUGCCA	miRNA	GAGGAACUAGCCUUCUCUCAGCUU	24	50	-0.167	-0.167	0.208	0.292	0.208	0.292	Mus musculus	https://mirbase.org/mature/MIMAT0017007
498	Sequence 4	UUCUCAGCCACUGUUGCCA	miRNA	CGCAUUUUCUCAGCAGCCAAG	21	52.38	-0.273	0	0.238	0.333	0.19	0.238	Vitis vinifera	https://mirbase.org/mature/MIMAT0018021
499	Sequence 4	UUCUCAGCCACUGUUGCCA	miRNA	GGCUUUCUAGUCUCAGCUCUCC	22	54.55	-0.333	-0.6	0.091	0.364	0.182	0.364	Homo sapiens	https://mirbase.org/mature/MIMAT0019212
500	Sequence 4	UUCUCAGCCACUGUUGCCA	miRNA	UGUAAACACCCUACACUCUCAGC	23	47.83	-0.636	0.167	0.304	0.391	0.087	0.217	Petromyzon marinus;Capra hircus	https://mirbase.org/mature/MIMAT0019430;https://mirbase.org/mature/MIMAT0036130
501	Sequence 4	UUCUCAGCCACUGUUGCCA	miRNA	UGUAAACAUCCUUCACUCUCAGC	23	43.48	-0.6	-0.077	0.261	0.348	0.087	0.304	Petromyzon marinus	https://mirbase.org/mature/MIMAT0019431
502	Sequence 4	UUCUCAGCCACUGUUGCCA	miRNA	CAAUCUCAGCUGGCAACUGUGAG	23	52.17	0	0.091	0.261	0.261	0.261	0.217	Petromyzon marinus	https://mirbase.org/mature/MIMAT0019525
503	Sequence 4	UUCUCAGCCACUGUUGCCA	miRNA	AUUGUCUCUCAGCUGCUUCAUC	22	45.45	-0.4	-0.5	0.136	0.318	0.136	0.409	Caenorhabditis elegans	https://mirbase.org/mature/MIMAT0020335
504	Sequence 4	UUCUCAGCCACUGUUGCCA	miRNA	UCAUGGAAUCUCAGCUGCUGCA	22	50	-0.091	-0.091	0.227	0.273	0.227	0.273	Medicago truncatula	https://mirbase.org/mature/MIMAT0021177
505	Sequence 4	UUCUCAGCCACUGUUGCCA	miRNA	UCUCAGCCAGGGCAGUAACAG	21	57.14	0	0.333	0.286	0.286	0.286	0.143	Oryza sativa	https://mirbase.org/mature/MIMAT0022162
506	Sequence 4	UUCUCAGCCACUGUUGCCA	miRNA	UGAACAUGCUUGUAAUCUCAGCA	23	39.13	-0.111	0	0.304	0.217	0.174	0.304	Rattus norvegicus	https://mirbase.org/mature/MIMAT0025058
507	Sequence 4	UUCUCAGCCACUGUUGCCA	miRNA	UUCUCAGCUACAUCGGUGUAAAUC	24	41.67	-0.2	-0.143	0.25	0.25	0.167	0.333	Taeniopygia guttata	https://mirbase.org/mature/MIMAT0025399
508	Sequence 4	UUCUCAGCCACUGUUGCCA	miRNA	UUGGAAUAGGGGAUAUCUCAGC	22	45.45	0.4	0	0.273	0.136	0.318	0.273	Homo sapiens;Macaca mulatta	https://mirbase.org/mature/MIMAT0025466;https://mirbase.org/mature/MIMAT0028248

	Sequence ID	siRNA Sequence	Type	Sequence	Length	GC %	GC skew	AT/AU Skew	MNC A	MNC C	MNC G	MNC U	Species	Link
509	Sequence 4	UUCUCAGCCACUGUUGCCA	miRNA	CAGGCAUCUUCUCAGCCUACAUGUG	25	52	-0.231	-0.167	0.2	0.32	0.2	0.28	Gallus gallus	https://mirbase.org/mature/MIMAT0026645
510	Sequence 4	UUCUCAGCCACUGUUGCCA	miRNA	GCAGGCAUCUUCUCAGCCUACA	22	54.55	-0.333	0	0.227	0.364	0.182	0.227	Taeniopygia guttata	https://mirbase.org/mature/MIMAT0026964
511	Sequence 4	UUCUCAGCCACUGUUGCCA	miRNA	CUCUGAUCGCCCUCUCAGCUC	21	61.9	-0.538	-0.5	0.095	0.476	0.143	0.286	Homo sapiens	https://mirbase.org/mature/MIMAT0027027
512	Sequence 4	UUCUCAGCCACUGUUGCCA	miRNA	UCUCAGCUGCUGCCCUCUCCAG	22	63.64	-0.429	-0.5	0.091	0.455	0.182	0.273	Homo sapiens	https://mirbase.org/mature/MIMAT0027475
513	Sequence 4	UUCUCAGCCACUGUUGCCA	miRNA	UCUCAGCCUUUAUCUCCCCAG	21	52.38	-0.636	-0.4	0.143	0.429	0.095	0.333	Mus musculus	https://mirbase.org/mature/MIMAT0028005
514	Sequence 4	UUCUCAGCCACUGUUGCCA	miRNA	UGCUUCCUUUCUCAGCUG	18	50	-0.333	-0.778	0.056	0.333	0.167	0.444	Homo sapiens	https://mirbase.org/mature/MIMAT0028234
515	Sequence 4	UUCUCAGCCACUGUUGCCA	miRNA	GCUCUCAGCCAGGUCUGAGGU	21	61.9	0.077	-0.25	0.143	0.286	0.333	0.238	Macaca mulatta	https://mirbase.org/mature/MIMAT0028341
516	Sequence 4	UUCUCAGCCACUGUUGCCA	miRNA	CACUGGGAUCAUCUCAGCAGAG	22	54.55	0	0.2	0.273	0.273	0.273	0.182	Gallus gallus	https://mirbase.org/mature/MIMAT0029071
517	Sequence 4	UUCUCAGCCACUGUUGCCA	miRNA	UGCAUUCUUAUCCUGUCUCAGC	22	45.45	-0.4	-0.5	0.136	0.318	0.136	0.409	Pristionchus pacificus	https://mirbase.org/mature/MIMAT0033040
518	Sequence 4	UUCUCAGCCACUGUUGCCA	miRNA	CCUAUCUCAGCUAUGUCACUA	21	42.86	-0.556	-0.167	0.238	0.333	0.095	0.333	Plutella xylostella	https://mirbase.org/mature/MIMAT0033790
519	Sequence 4	UUCUCAGCCACUGUUGCCA	miRNA	UGUAAACAUCCUACACUCUCAGCUG	25	44	-0.455	0	0.28	0.32	0.12	0.28	Eptesicus fuscus;Xenopus laevis	https://mirbase.org/mature/MIMAT0035192;https://mirbase.org/mature/MIMAT0046572
520	Sequence 4	UUCUCAGCCACUGUUGCCA	miRNA	UCUCAGCUACGUCGGUGUAAAUC	23	47.83	-0.091	-0.167	0.217	0.261	0.217	0.304	Columba livia	https://mirbase.org/mature/MIMAT0038680
521	Sequence 4	UUCUCAGCCACUGUUGCCA	miRNA	GCAGGCAUCUUCUCAGCCUAC	21	57.14	-0.333	-0.111	0.19	0.381	0.19	0.238	Python bivittatus	https://mirbase.org/mature/MIMAT0039059
522	Sequence 4	UUCUCAGCCACUGUUGCCA	miRNA	AGGGAGGCGCUCACUCUCAGCU	22	63.64	0	0	0.182	0.318	0.318	0.182	Callithrix jacchus	https://mirbase.org/mature/MIMAT0039659
523	Sequence 4	UUCUCAGCCACUGUUGCCA	miRNA	CUCAGGCUCUCAGCCCGAGCC	21	71.43	-0.333	0	0.143	0.476	0.238	0.143	Pteropus alecto	https://mirbase.org/mature/MIMAT0040267
524	Sequence 4	UUCUCAGCCACUGUUGCCA	miRNA	GGUGGAAUGGUCUCAGCGAGC	21	61.9	0.385	0	0.19	0.19	0.429	0.19	Heligmosomoides polygyrus	https://mirbase.org/mature/MIMAT0040805
525	Sequence 4	UUCUCAGCCACUGUUGCCA	miRNA	UACCAUCUCUCAGCUUCAUU	20	40	-0.75	-0.333	0.2	0.35	0.05	0.4	Echinococcus granulosus	https://mirbase.org/mature/MIMAT0041296
526	Sequence 4	UUCUCAGCCACUGUUGCCA	miRNA	CCAGGGAGCCCGUCUCAGCGU	21	71.43	-0.067	0	0.143	0.381	0.333	0.143	Gadus morhua	https://mirbase.org/mature/MIMAT0044420
527	Sequence 4	UUCUCAGCCACUGUUGCCA	miRNA	UGAGAAUGAUGUGGAUCUCAGC	22	45.45	0.4	0	0.273	0.136	0.318	0.273	Fragaria vesca	https://mirbase.org/mature/MIMAT0044594
528	Sequence 4	UUCUCAGCCACUGUUGCCA	miRNA	UCUCAGCAGAUUUAAUCCCCGA	22	45.45	-0.4	0	0.273	0.318	0.136	0.273	Picea abies	https://mirbase.org/mature/MIMAT0045093
529	Sequence 4	UUCUCAGCCACUGUUGCCA	miRNA	GAACCUGAAAUUUUGUCUCAGCC	23	43.48	-0.2	-0.077	0.261	0.261	0.174	0.304	Parasteatoda tepidariorum	https://mirbase.org/mature/MIMAT0046229
530	Sequence 4	UUCUCAGCCACUGUUGCCA	miRNA	UUGUCCUACUUCUCAGCUGUCU	22	45.45	-0.4	-0.667	0.091	0.318	0.136	0.455	Bos taurus	https://mirbase.org/mature/MIMAT0046648
531	Sequence 4	UUCUCAGCCACUGUUGCCA	miRNA	UCAGGCUCUCAGCCCGAGCC	20	70	-0.286	0	0.15	0.45	0.25	0.15	Cavia porcellus	https://mirbase.org/mature/MIMAT0047423
532	Sequence 4	UUCUCAGCCACUGUUGCCA	miRNA	CAUCUCAGCAACCAAUUGAACU	22	40.91	-0.556	0.231	0.364	0.318	0.091	0.227	Dasypus novemcinctus	https://mirbase.org/mature/MIMAT0048042
533	Sequence 4	UUCUCAGCCACUGUUGCCA	miRNA	CGUUGGAAGAUUGGAUCUCAGCA	23	47.83	0.273	0	0.261	0.174	0.304	0.261	Melibe leonina	https://mirbase.org/mature/MIMAT0048795
534	Sequence 4	UUCUCAGCCACUGUUGCCA	miRNA	AUUUCACUCUCAGCAGGCUCC	21	52.38	-0.455	-0.2	0.19	0.381	0.143	0.286	Gallus gallus	https://mirbase.org/mature/MIMAT0049895
535	Sequence 4	UUCUCAGCCACUGUUGCCA	piRNA	UGGUGCAUGCCUGUGGUCUCAGCUACUCA	29	55.17	0	-0.385	0.138	0.276	0.276	0.31	Homo sapiens	https://dashr2.lisanwanglab.org/get_bg_smrna.php?coord=chr10:17893280-17893308:neg&rnaID=piR-57387&dataSource=DASHR2_GEO_hg38;https://dashr2.lisanwanglab.org/get_bg_smrna.php?coord=chr10:43187818-43187846:neg&rnaID=piR-57387&dataSource=DASHR2_GEO_hg38;https://dashr2.lisanwanglab.org/get_bg_smrna.php?coord=chr10:58185360-58185388:neg&rnaID=piR-57387&dataSource=DASHR2_GEO_hg38;https://dashr2.lisanwanglab.org/get_bg_smrna.php?coord=chr11:17318139-[illegible] … php?coord=chr16:72709145-72709173:pos&rnaID=piR-57387&dataSource=DASHR2
536	Sequence 4	UUCUCAGCCACUGUUGCCA	piRNA	UUUCAAGUGAUUCUCCUGUCUCAGCCUCC	29	48.28	-0.429	-0.467	0.138	0.345	0.138	0.379	Homo sapiens	https://dashr2.lisanwanglab.org/get_bg_smrna.php?coord=chr10:[illegible]&rnaID=piR-61861&dataSource=DASHR2 … [illegible]
537	Sequence 4	UUCUCAGCCACUGUUGCCA	piRNA	UCUCAGCAAGGCAAUCUUUACUUUCUGCAGA	31	41.94	-0.231	-0.111	0.258	0.258	0.161	0.323	Homo sapiens	https://dashr2.lisanwanglab.org/get_bg_smrna.php?coord=chr10:45070663-[illegible]&rnaID=piR-45517&dataSource=DASHR2 … [illegible]
538	Sequence 4	UUCUCAGCCACUGUUGCCA	piRNA	UGAGACAAAGGAUCUCUCAGCAAGGCUAGU	30	46.67	0.143	0.25	0.333	0.2	0.267	0.2	Homo sapiens	https://dashr2.lisanwanglab.org/get_bg_smrna.php?coord=chr11:70038501-[illegible]&rnaID=piR-48348&dataSource=DASHR2 … [illegible]
539	Sequence 4	UUCUCAGCCACUGUUGCCA	piRNA	UGCUCUCCGUUGAUCUUCUCAGCUAAGAUG	30	46.67	-0.143	-0.375	0.167	0.267	0.2	0.367	Homo sapiens	https://dashr2.lisanwanglab.org/get_bg_smrna.php?coord=chr10:79702172-[illegible]&rnaID=piR-52896&dataSource=DASHR2_GEO_hg38 … [illegible]
540	Sequence 4	UUCUCAGCCACUGUUGCCA	piRNA	UGGGUGUUUGAAAUCAGGAUGGUCUCAGCC	30	50	0.333	-0.2	0.2	0.167	0.333	0.3	Homo sapiens	https://dashr2.lisanwanglab.org/get_bg_smrna.php?coord=chr10:[illegible]&rnaID=piR-56760&dataSource=DASHR2_GEO_hg38 … [illegible]
541	Sequence 4	UUCUCAGCCACUGUUGCCA	piRNA	UGGAGUCUAGUGGUCUCAGCAGGCAUC	27	55.56	0.2	-0.167	0.185	0.222	0.333	0.259	Homo sapiens	https://dashr2.lisanwanglab.org/get_bg_smrna.php?coord=chr11:45716993-[illegible]&rnaID=piR-54292&dataSource=DASHR2_GEO_hg38
542	Sequence 4	UUCUCAGCCACUGUUGCCA	piRNA	UGGAUGUUCCGGCCACCCCACUCUCAGC	28	64.29	-0.333	-0.2	0.143	0.429	0.214	0.214	Homo sapiens	https://dashr2.lisanwanglab.org/get_bg_smrna.php?coord=chr11:78226704-[illegible]&rnaID=piR-54489&dataSource=DASHR2_GEO_hg38
543	Sequence 4	UUCUCAGCCACUGUUGCCA	piRNA	GACCUUGGCACAGACUCUCAGCGCCUCC	28	64.29	-0.333	0	0.179	0.429	0.214	0.179	Homo sapiens	https://dashr2.lisanwanglab.org/get_bg_smrna.php?coord=chr13:25018014-[illegible]&rnaID=piR-34432&dataSource=DASHR2_GEO_hg38
544	Sequence 4	UUCUCAGCCACUGUUGCCA	piRNA	UGAAACCAUCUCAGCCUGGACUUCAUUGGUC	31	48.39	-0.2	-0.125	0.226	0.29	0.194	0.29	Homo sapiens	https://dashr2.lisanwanglab.org/get_bg_smrna.php?coord=chr13:31315306-[illegible]&rnaID=piR-46668&dataSource=DASHR2_GEO_hg38
545	Sequence 4	UUCUCAGCCACUGUUGCCA	piRNA	UGAUGAGGUGACAAAGAGAUUCUCAGC	27	44.44	0.333	0.2	0.333	0.148	0.296	0.222	Homo sapiens	https://dashr2.lisanwanglab.org/get_bg_smrna.php?coord=chr14:25430017-[illegible]&rnaID=piR-49834&dataSource=DASHR2_GEO_hg38 … [illegible]
546	Sequence 4	UUCUCAGCCACUGUUGCCA	piRNA	UGCCACUCUUUUGCCAGUUCUCAGCCU	27	51.85	-0.429	-0.538	0.111	0.37	0.148	0.37	Homo sapiens	https://dashr2.lisanwanglab.org/get_bg_smrna.php?coord=chr14:54778175-[illegible]&rnaID=piR-51416&dataSource=DASHR2_GEO_hg38
547	Sequence 4	UUCUCAGCCACUGUUGCCA	piRNA	UAUUAAGUGGAAGUAGCUCUCAGCAGAUGG	30	43.33	0.385	0.059	0.3	0.133	0.3	0.267	Homo sapiens	https://dashr2.lisanwanglab.org/get_bg_smrna.php?coord=chr14:88146452-[illegible]&rnaID=piR-40511&dataSource=DASHR2_GEO_hg38 … [illegible]
548	Sequence 4	UUCUCAGCCACUGUUGCCA	piRNA	UAAGUGGAAGUAGCUCUCAGCAGAUG	26	46.15	0.333	0.143	0.308	0.154	0.308	0.231	Homo sapiens	https://dashr2.lisanwanglab.org/get_bg_smrna.php?coord=chr14:88146453-[illegible]&rnaID=piR-37557&dataSource=DASHR2_GEO_hg38 … [illegible]
549	Sequence 4	UUCUCAGCCACUGUUGCCA	piRNA	UCUCUCCUGAUCUCAGCAGAGAGGGUACCU	30	53.33	-0.125	-0.143	0.2	0.3	0.233	0.267	Homo sapiens	https://dashr2.lisanwanglab.org/get_bg_smrna.php?coord=chr14:88147654-[illegible]&rnaID=piR-45703&dataSource=DASHR2_GEO_hg38
550	Sequence 4	UUCUCAGCCACUGUUGCCA	piRNA	UGGUUGUCCUGUGGAGUGUUCAGCUCUCAGC	31	54.84	0.176	-0.571	0.097	0.226	0.323	0.355	Homo sapiens	https://dashr2.lisanwanglab.org/get_bg_smrna.php?coord=chr14:88147738-[illegible]&rnaID=piR-57709&dataSource=DASHR2_GEO_hg38
551	Sequence 4	UUCUCAGCCACUGUUGCCA	piRNA	UCUCAGCCCCAUGAUUCUAUGAGUCUGUGA	30	46.67	-0.143	-0.25	0.2	0.267	0.2	0.333	Homo sapiens	https://dashr2.lisanwanglab.org/get_bg_smrna.php?coord=chr15:51302392-[illegible]&rnaID=piR-45523&dataSource=DASHR2_GEO_hg38

	Sequence ID	siRNA Sequence	Type	Sequence	Length	GC %	GC skew	AT/AU Skew	MNC A	MNC C	MNC G	MNC U	Species	Link
552	Sequence 4	UUCUCAGCCACUGUUGCCA	piRNA	UUCUCAGCCCUCAUGCUAUGUAGAAAAGGA	30	43.33	-0.077	0.059	0.3	0.233	0.2	0.267	Homo sapiens	https://dashr2.lisanwanglab.org/get_bg_smrna.php?coord=chr15:62221473-62221502:neg&rnaID=piR-60763&dataSource=DASHR2_GEO_hg38
553	Sequence 4	UUCUCAGCCACUGUUGCCA	piRNA	UGCCCAAAUAAACUCUCAGCCAUGCUUAGCAA	32	43.75	-0.429	0.222	0.344	0.313	0.125	0.219	Homo sapiens	https://dashr2.lisanwanglab.org/get_bg_smrna.php?coord=chr15:62240400-62240431:pos&rnaID=piR-51716&dataSource=DASHR2_GEO_hg38
554	Sequence 4	UUCUCAGCCACUGUUGCCA	piRNA	UCUCAGCCAUGCUUAGCAAGAAGCUGCAUA	30	46.67	-0.143	0.125	0.3	0.267	0.2	0.233	Homo sapiens	https://dashr2.lisanwanglab.org/get_bg_smrna.php?coord=chr15:62240413-62240442:pos&rnaID=piR-45522&dataSource=DASHR2_GEO_hg38
555	Sequence 4	UUCUCAGCCACUGUUGCCA	piRNA	UCGUCUUGUUCCAACAAUCUCAGCAACCUCU	31	45.16	-0.571	-0.176	0.226	0.355	0.097	0.323	Homo sapiens	https://dashr2.lisanwanglab.org/get_bg_smrna.php?coord=chr15:62246144-62246174:pos&rnaID=piR-45218&dataSource=DASHR2_GEO_hg38
556	Sequence 4	UUCUCAGCCACUGUUGCCA	piRNA	UGUUCCAACAAUCUCAGCAACCUCUUCC	28	46.43	-0.692	-0.067	0.25	0.393	0.071	0.286	Homo sapiens	https://dashr2.lisanwanglab.org/get_bg_smrna.php?coord=chr15:62246150-62246177:pos&rnaID=piR-59333&dataSource=DASHR2_GEO_hg38
557	Sequence 4	UUCUCAGCCACUGUUGCCA	piRNA	UCCUUUCAGAUUCUCAGCGUGUUGAUC	27	44.44	-0.167	-0.467	0.148	0.259	0.185	0.407	Homo sapiens	https://dashr2.lisanwanglab.org/get_bg_smrna.php?coord=chr15:62255363-62255389:pos&rnaID=piR-44846&dataSource=DASHR2_GEO_hg38
558	Sequence 4	UUCUCAGCCACUGUUGCCA	piRNA	UGCAGGUGUUCUUGCUCUCAGCUCAGA	27	51.85	0	-0.385	0.148	0.259	0.259	0.333	Homo sapiens	https://dashr2.lisanwanglab.org/get_bg_smrna.php?coord=chr15:98950364-98950390:pos&rnaID=piR-50907&dataSource=DASHR2_GEO_hg38
559	Sequence 4	UUCUCAGCCACUGUUGCCA	piRNA	CCCAGAGUGCACUGGUGGAAUCUCAGC	27	59.26	0	0.091	0.222	0.296	0.296	0.185	Homo sapiens	https://dashr2.lisanwanglab.org/get_bg_smrna.php?coord=chr16:517514-517540:neg&rnaID=piR-32963&dataSource=DASHR2_GEO_hg38
560	Sequence 4	UUCUCAGCCACUGUUGCCA	piRNA	UGGGUCUCACCAUACUUUCUCAGCGUGGG	29	55.17	0	-0.385	0.138	0.276	0.276	0.31	Homo sapiens	https://dashr2.lisanwanglab.org/get_bg_smrna.php?coord=chr16:25070563-25070591:pos&rnaID=piR-56600&dataSource=DASHR2_GEO_hg38
561	Sequence 4	UUCUCAGCCACUGUUGCCA	piRNA	UAGGCCAGAUUGGGAAGACUUUUCUCAGC	29	48.28	0.143	-0.067	0.241	0.207	0.276	0.276	Homo sapiens	https://dashr2.lisanwanglab.org/get_bg_smrna.php?coord=chr17:7366857-7366885:pos&rnaID=piR-39663&dataSource=DASHR2_GEO_hg38;https://dashr2.lisanwanglab.org/get_bg_smrna.php?coord=chr9:112513343-112513371:pos&rnaID=piR-39663&dataSource=DASHR2_GEO_hg38
562	Sequence 4	UUCUCAGCCACUGUUGCCA	piRNA	UGUGCAGACGAAUCUCUCAGCAGAGUUCA	29	48.28	0	0.067	0.276	0.241	0.241	0.241	Homo sapiens	https://dashr2.lisanwanglab.org/get_bg_smrna.php?coord=chr17:28272749-28272777:neg&rnaID=piR-58758&dataSource=DASHR2_GEO_hg38
563	Sequence 4	UUCUCAGCCACUGUUGCCA	piRNA	UGUUGUGCAGACGAAUCUCUCAGCAGAGUU	30	46.67	0.143	-0.125	0.233	0.2	0.267	0.3	Homo sapiens	https://dashr2.lisanwanglab.org/get_bg_smrna.php?coord=chr17:28272751-28272780:neg&rnaID=piR-59492&dataSource=DASHR2_GEO_hg38
564	Sequence 4	UUCUCAGCCACUGUUGCCA	piRNA	UCCCCCAAGAACAGGGAUCACAUUCUCAGC	30	53.33	-0.375	0.286	0.3	0.367	0.167	0.167	Homo sapiens	https://dashr2.lisanwanglab.org/get_bg_smrna.php?coord=chr17:32609398-32609427:neg&rnaID=piR-43518&dataSource=DASHR2_GEO_hg38
565	Sequence 4	UUCUCAGCCACUGUUGCCA	piRNA	UGAGGCUUUGAAACUCUCAGCCAGCUC	27	51.85	-0.143	-0.077	0.222	0.296	0.222	0.259	Homo sapiens	https://dashr2.lisanwanglab.org/get_bg_smrna.php?coord=chr18:13562311-13562337:pos&rnaID=piR-49064&dataSource=DASHR2_GEO_hg38
566	Sequence 4	UUCUCAGCCACUGUUGCCA	piRNA	ACUCAUCUCAGCAGGGAUGUAGCUCCUGGA	30	53.33	0	0	0.233	0.267	0.267	0.233	Homo sapiens	https://dashr2.lisanwanglab.org/get_bg_smrna.php?coord=chr19:19747983-19748012:neg&rnaID=piR-30753&dataSource=DASHR2_GEO_hg38
567	Sequence 4	UUCUCAGCCACUGUUGCCA	piRNA	GACUCAUCUCAGCAGGGAUGUAGCUCCUGG	30	56.67	0.059	-0.077	0.2	0.267	0.3	0.233	Homo sapiens	https://dashr2.lisanwanglab.org/get_bg_smrna.php?coord=chr19:19747984-19748013:neg&rnaID=piR-34487&dataSource=DASHR2_GEO_hg38
568	Sequence 4	UUCUCAGCCACUGUUGCCA	piRNA	UGAUUUGCUCUCAGCAGUGCCGGCUUCUCC	30	56.67	-0.176	-0.538	0.1	0.333	0.233	0.333	Homo sapiens	https://dashr2.lisanwanglab.org/get_bg_smrna.php?coord=chr19:34890794-34890823:neg&rnaID=piR-50167&dataSource=DASHR2_GEO_hg38
569	Sequence 4	UUCUCAGCCACUGUUGCCA	piRNA	UUGACUGGGGCAGGAAGAGAAGGAUCUCAGC	31	54.84	0.412	0.286	0.29	0.161	0.387	0.161	Homo sapiens	https://dashr2.lisanwanglab.org/get_bg_smrna.php?coord=chr19:34891036-34891066:neg&rnaID=piR-61023&dataSource=DASHR2_GEO_hg38
570	Sequence 4	UUCUCAGCCACUGUUGCCA	piRNA	CGAUCUCAGCAAGAGGAAUGCGGUAG	26	53.85	0.286	0.333	0.308	0.192	0.346	0.154	Homo sapiens	https://dashr2.lisanwanglab.org/get_bg_smrna.php?coord=chr2:218096307-218096332:neg&rnaID=piR-33370&dataSource=DASHR2_GEO_hg38
571	Sequence 4	UUCUCAGCCACUGUUGCCA	piRNA	UCCUCUCAGCAUCCAGCAGUUUCUGCC	27	55.56	-0.467	-0.333	0.148	0.407	0.148	0.296	Homo sapiens	https://dashr2.lisanwanglab.org/get_bg_smrna.php?coord=chr21:44485568-44485594:neg&rnaID=piR-44379&dataSource=DASHR2_GEO_hg38
572	Sequence 4	UUCUCAGCCACUGUUGCCA	piRNA	UAUUGAGUGGAAGCAGCUCUCAGCCGACGGA	31	54.84	0.176	0.143	0.258	0.226	0.323	0.194	Homo sapiens	https://dashr2.lisanwanglab.org/get_bg_smrna.php?coord=chr21:44485790-44485820:neg&rnaID=piR-40567&dataSource=DASHR2_GEO_hg38
573	Sequence 4	UUCUCAGCCACUGUUGCCA	piRNA	UAUUGAGUGGAAGCAGCUCUCAGCUGAUGGA	31	48.39	0.333	0	0.258	0.161	0.323	0.258	Homo sapiens	https://dashr2.lisanwanglab.org/get_bg_smrna.php?coord=chr21:44490739-44490769:neg&rnaID=piR-40569&dataSource=DASHR2_GEO_hg38
574	Sequence 4	UUCUCAGCCACUGUUGCCA	piRNA	UUGAGUGGAAGCAGCUCUCAGCUGAU	26	50	0.231	-0.077	0.231	0.192	0.308	0.269	Homo sapiens	https://dashr2.lisanwanglab.org/get_bg_smrna.php?coord=chr21:44490742-44490767:neg&rnaID=piR-61072&dataSource=DASHR2_GEO_hg38
575	Sequence 4	UUCUCAGCCACUGUUGCCA	piRNA	UGUUAUUGAGUGGAAGCAGCUCUCAGCUGA	30	46.67	0.286	-0.125	0.233	0.167	0.3	0.3	Homo sapiens	https://dashr2.lisanwanglab.org/get_bg_smrna.php?coord=chr21:44490743-44490772:neg&rnaID=piR-59309&dataSource=DASHR2_GEO_hg38
576	Sequence 4	UUCUCAGCCACUGUUGCCA	piRNA	UGCAGAGGUCUCAGCAUGAGGUGGAGC	27	59.26	0.375	0.091	0.222	0.185	0.407	0.185	Homo sapiens	https://dashr2.lisanwanglab.org/get_bg_smrna.php?coord=chr22:22630829-22630855:neg&rnaID=piR-50721&dataSource=DASHR2_GEO_hg38;https://dashr2.lisanwanglab.org/get_bg_smrna.php?coord=chr22:23310192-23310218:neg&rnaID=piR-50721&dataSource=DASHR2_GEO_hg38;https://dashr2.lisanwanglab.org/get_bg_smrna.php?coord=chr22:24645504-24645530:neg&rnaID=piR-50721&dataSource=DASHR2_GEO_hg38
577	Sequence 4	UUCUCAGCCACUGUUGCCA	piRNA	UCUCAGCAGAUGACAUAGUACCACCA	26	46.15	-0.333	0.286	0.346	0.308	0.154	0.192	Homo sapiens	https://dashr2.lisanwanglab.org/get_bg_smrna.php?coord=chr22:23897301-23897326:neg&rnaID=piR-45518&dataSource=DASHR2_GEO_hg38
578	Sequence 4	UUCUCAGCCACUGUUGCCA	piRNA	UGAGAUGUUCUCAGCUUGGAAUCCCGUGG	29	51.72	0.2	-0.286	0.172	0.207	0.31	0.31	Homo sapiens	https://dashr2.lisanwanglab.org/get_bg_smrna.php?coord=chr3:12859727-12859755:neg&rnaID=piR-48599&dataSource=DASHR2_GEO_hg38
579	Sequence 4	UUCUCAGCCACUGUUGCCA	piRNA	UAUUCUCAGCCCUAUGUCUUACUCCA	26	42.31	-0.636	-0.333	0.192	0.346	0.077	0.385	Homo sapiens	https://dashr2.lisanwanglab.org/get_bg_smrna.php?coord=chr3:20446802-20446827:pos&rnaID=piR-40547&dataSource=DASHR2_GEO_hg38
580	Sequence 4	UUCUCAGCCACUGUUGCCA	piRNA	AGAGUUGUCUCAGCCCAGUGGAGCA	26	53.85	0.143	0	0.231	0.231	0.308	0.231	Homo sapiens	https://dashr2.lisanwanglab.org/get_bg_smrna.php?coord=chr3:130003803-130003828:pos&rnaID=piR-30962&dataSource=DASHR2_GEO_hg38;https://dashr2.lisanwanglab.org/get_bg_smrna.php?coord=chr3:75580995-75581020:pos&rnaID=piR-30962&dataSource=DASHR2_GEO_hg38;https://dashr2.lisanwanglab.org/get_bg_smrna.php?coord=chr4:4108292-4108317:pos&rnaID=piR-30962&dataSource=DASHR2_GEO_hg38;https://dashr2.lisanwanglab.org/get_bg_smrna.php?coord=chr4:9000020-9000045:pos&rnaID=piR-30962&dataSource=DASHR2_GEO_hg38
581	Sequence 4	UUCUCAGCCACUGUUGCCA	piRNA	UAUCCCUAGAAUCAAUCUCUCAGCAUCA	29	37.93	-0.636	0	0.31	0.31	0.069	0.31	Homo sapiens	https://dashr2.lisanwanglab.org/get_bg_smrna.php?coord=chr5:12028319-12028347:neg&rnaID=piR-60256&dataSource=DASHR2_GEO_hg38
582	Sequence 4	UUCUCAGCCACUGUUGCCA	piRNA	UUGUCUCAGCCACCAUGAGUGGUGUCACCACC	32	56.25	-0.222	-0.143	0.188	0.344	0.219	0.25	Homo sapiens	https://dashr2.lisanwanglab.org/get_bg_smrna.php?coord=chr6:30723745-30723776:neg&rnaID=piR-61749&dataSource=DASHR2_GEO_hg38
583	Sequence 4	UUCUCAGCCACUGUUGCCA	piRNA	UCUCAGCUUCCCGCGACUCCCACCUCCA	28	64.29	-0.667	-0.2	0.143	0.536	0.107	0.214	Homo sapiens	https://dashr2.lisanwanglab.org/get_bg_smrna.php?coord=chr6:33870218-33870246:neg&rnaID=piR-46535&dataSource=DASHR2_GEO_hg38
584	Sequence 4	UUCUCAGCCACUGUUGCCA	piRNA	UGCCGCCUUGCUGGGGACUUGAGCCUCUCAGC	32	65.62	-0.048	-0.455	0.094	0.344	0.313	0.25	Homo sapiens	https://dashr2.lisanwanglab.org/get_bg_smrna.php?coord=chr6:33896222-33896253:neg&rnaID=piR-51098&dataSource=DASHR2_GEO_hg38
585	Sequence 4	UUCUCAGCCACUGUUGCCA	piRNA	UCAUCAGGUCAGACUCUCAGCACACA	26	50	-0.385	0.231	0.308	0.346	0.154	0.192	Homo sapiens	https://dashr2.lisanwanglab.org/get_bg_smrna.php?coord=chr6:70860296-70860321:neg&rnaID=piR-61803&dataSource=DASHR2_GEO_hg38
586	Sequence 4	UUCUCAGCCACUGUUGCCA	piRNA	UCUCAGCCACAAUUCUGGAAUAACAGGA	28	42.86	-0.167	0.25	0.357	0.25	0.179	0.214	Homo sapiens	https://dashr2.lisanwanglab.org/get_bg_smrna.php?coord=chr6:117672608-117672635:neg&rnaID=piR-55525&dataSource=DASHR2_GEO_hg38
587	Sequence 4	UUCUCAGCCACUGUUGCCA	piRNA	UGCAGCUUCAACAGUUUCUCAGCAUGUUCCC	31	48.39	-0.333	-0.25	0.194	0.323	0.161	0.323	Homo sapiens	https://dashr2.lisanwanglab.org/get_bg_smrna.php?coord=chr9:15527463-15527493:pos&rnaID=piR-50811&dataSource=DASHR2_GEO_hg38
588	Sequence 4	UUCUCAGCCACUGUUGCCA	piRNA	UCAGCUCAACUUCUCAGCCAAUGAGUU	27	44.44	-0.333	-0.067	0.259	0.296	0.148	0.296	Homo sapiens	https://dashr2.lisanwanglab.org/get_bg_smrna.php?coord=chr9:33676531-33676557:pos&rnaID=piR-61651&dataSource=DASHR2_GEO_hg38;https://dashr2.lisanwanglab.org/get_bg_smrna.php?coord=chr9:38567669-38567695:pos&rnaID=piR-61651&dataSource=DASHR2_GEO_hg38
589	Sequence 4	UUCUCAGCCACUGUUGCCA	piRNA	UCAACUUCUCAGCCAAUGAGUUGACAUGC	29	44.83	-0.231	0	0.276	0.276	0.172	0.276	Homo sapiens	https://dashr2.lisanwanglab.org/get_bg_smrna.php?coord=chr9:33676536-33676564:pos&rnaID=piR-60025&dataSource=DASHR2_GEO_hg38;https://dashr2.lisanwanglab.org/get_bg_smrna.php?coord=chr9:38567760-38567788:pos&rnaID=piR-60025&dataSource=DASHR2_GEO_hg38
590	Sequence 4	UUCUCAGCCACUGUUGCCA	piRNA	CUCAUAGCCAGAUCUGCAGGUCUCAGC	27	55.56	-0.2	0	0.222	0.333	0.222	0.222	Homo sapiens	https://dashr2.lisanwanglab.org/get_bg_smrna.php?coord=chr9:69067727-69067753:pos&rnaID=piR-33690&dataSource=DASHR2_GEO_hg38
591	Sequence 4	UUCUCAGCCACUGUUGCCA	piRNA	UGGAAGCAAUGGUGUUCUUCUCAGCC	26	50	0.077	-0.231	0.192	0.231	0.269	0.308	Homo sapiens	https://dashr2.lisanwanglab.org/get_bg_smrna.php?coord=chrX:9406812-9406837:neg&rnaID=piR-53704&dataSource=DASHR2_GEO_hg38;https://dashr2.lisanwanglab.org/get_bg_smrna.php?coord=chrX:9407743-9407768:neg&rnaID=piR-53704&dataSource=DASHR2_GEO_hg38;https://dashr2.lisanwanglab.org/get_bg_smrna.php?coord=chrX:9408674-9408699:neg&rnaID=piR-53704&dataSource=DASHR2_GEO_hg38;https://dashr2.lisanwanglab.org/get_bg_smrna.php?coord=chrX:9409605-9409630:neg&rnaID=piR-53704&dataSource=DASHR2_GEO_hg38;https://dashr2.lisanwanglab.org/get_bg_smrna.php?coord=chrX:9411581-9411606:neg&rnaID=piR-53704&dataSource=DASHR2_GEO_hg38;https://dashr2.lisanwanglab.org/get_bg_smrna.php?coord=chrX:9412513-9412538:neg&rnaID=piR-53704&dataSource=DASHR2_GEO_hg38;https://dashr2.lisanwanglab.org/get_bg_smrna.php?coord=chrX:9414603-9414628:neg&rnaID=piR-53704&dataSource=DASHR2
592	Sequence 4	UUCUCAGCCACUGUUGCCA	piRNA	UUCUCAGCCGGAACCCCAUGUGGUCA	26	57.69	-0.2	-0.091	0.192	0.346	0.231	0.231	Homo sapiens	https://dashr2.lisanwanglab.org/get_bg_smrna.php?coord=chrX:9407726-9407751:neg&rnaID=piR-60764&dataSource=DASHR2_GEO_hg38;https://dashr2.lisanwanglab.org/get_bg_smrna.php?coord=chrX:9408657-9408682:neg&rnaID=piR-60764&dataSource=DASHR2_GEO_hg38;https://dashr2.lisanwanglab.org/get_bg_smrna.php?coord=chrX:9409588-9409613:neg&rnaID=piR-60764&dataSource=DASHR2_GEO_hg38
